# Osteology Supports a Stem-Galliform Affinity for the Giant Extinct Flightless Bird *Sylviornis neocaledoniae* (Sylviornithidae, Galloanseres)

**DOI:** 10.1371/journal.pone.0150871

**Published:** 2016-03-30

**Authors:** Trevor H. Worthy, Miyess Mitri, Warren D. Handley, Michael S. Y. Lee, Atholl Anderson, Christophe Sand

**Affiliations:** 1 School of Biological Sciences, Flinders University, Adelaide, South Australia, Australia; 2 Earth Sciences Section, South Australian Museum, North Terrace, Adelaide, South Australia, Australia; 3 School of Biological Sciences, University of Adelaide, South Australia, Australia; 4 Archaeology and Natural History, College of Asia and the Pacific, Australian National University, Canberra, Australian Capital Territory, Australia; 5 Institut d’Archéologie de la Nouvelle-Calédonie et du Pacifique, BP 11423, 98802 Nouméa Cedex, New Caledonia; Monash University, AUSTRALIA

## Abstract

The giant flightless bird *Sylviornis neocaledoniae* (Aves: Sylviornithidae) existed on La Grande Terre and Ile des Pins, New Caledonia, until the late Holocene when it went extinct shortly after human arrival on these islands. The species was generally considered to be a megapode (Megapodiidae) until the family Sylviornithidae was erected for it in 2005 to reflect multiple cranial autapomorphies. However, despite thousands of bones having been reported for this unique and enigmatic taxon, the postcranial anatomy has remained largely unknown. We rectify this deficiency and describe the postcranial skeleton of *S*. *neocaledoniae* based on ~600 fossils and use data from this and its cranial anatomy to make a comprehensive assessment of its phylogenetic affinities. *Sylviornis neocaledoniae* is found to be a stem galliform, distant from megapodiids, and the sister taxon to the extinct flightless *Megavitiornis altirostris* from Fiji, which we transfer to the family Sylviornithidae. These two species form the sister group to extant crown-group galliforms. Several other fossil galloanseres also included in the phylogenetic analysis reveal novel hypotheses of their relationships as follows: *Dromornis planei* (Dromornithidae) is recovered as a stem galliform rather than a stem anseriform; *Presbyornis pervetus* (Presbyornithidae) is the sister group to Anseranatidae, not to Anatidae; *Vegavis iaai* is a crown anseriform but remains unresolved relative to *Presbyornis pervetus*, Anseranatidae and Anatidae. *Sylviornis neocaledoniae* was reconstructed herein to be 0.8 m tall in a resting stance and weigh 27–34 kg. The postcranial anatomy of *S*. *neocaledoniae* shows no indication of the specialised adaptation to digging seen in megapodiids, with for example, its ungual morphology differing little from that of chicken *Gallus gallus*. These observations and its phylogenetic placement as stem galliforms makes it improbable that this species employed ectothermic incubation or was a mound-builder. *Sylviornis neocaledoniae* can therefore be excluded as the constructor of tumuli in New Caledonia.

## Introduction

Avian evolution on islands has resulted in the evolution of large flightless forms in multiple groups [1, 2], such as the nine species of ratite moa (Dinornithiformes) of New Zealand [3], giant waterfowl (Anatidae) including moa-nalos in Hawaii [4] and geese in New Zealand [3], pigeons (Columbidae) in the Mascarenes [5, 6] and Fiji [7], and enigmatic gruiforms (Aptornithidae) in New Zealand (e.g., [8, 9]). Galliforms have also spawned giant flightless forms in the Pacific, with the best known being *Sylviornis neocaledoniae* Poplin, 1980 from New Caledonia.

*Sylviornis neocaledoniae* was originally described as a ratite [10], but its affinities were soon considered to lie with megapode galliforms [11–15]. Despite thousands of bones being known [14], its osteology is incompletely known except for the skull, which was described in detail by Mourer-Chauviré and Balouet [16], who convincingly showed that *S*. *neocaledoniae* was a galliform. However, they considered the shared features with megapodes to be symplesiomorphic and on the basis of its many autapomorphies, they established the monotypic Sylviornithidae for *S*. *neocaledoniae*. Notable features these authors highlighted included: the broad flattened cranium; a massive, dorsoventrally deep, laterally compressed rostrum with a large bony ornament; mandible with an elongated symphysis; and a zona flexoria craniofacialis that forms a synovial joint, hereafter termed a craniofacial hinge, that transects the nasals early in ontogeny. However, such autapomorphies might not preclude this taxon from being embedded within an established family as, for example, some ratites (e.g., *Casuarius* sp.) and galliforms (e.g. Numididae) have bony ornament on their skull, and some galliforms have such on their rostrum (e.g. some, but not all, cracids). Bill shape and size can be remarkably variable within groups, as well shown within Anatidae, for example, with extremes seen in larger flightless taxa, such as the large flightless Hawaiian moa-nalos [4]. Moreover, reduction in pectoral girdle complexity associated with flightlessness over a long time could easily have led to the loss of features. One such feature likely to be so impacted is the cup-like cotyla scapularis observed in stem galliforms [17] but absent in the crown group: its loss in *S*. *neocaledoniae* may not necessarily reflect the derived galliform state, contra Mourer-Chauviré and Balouet [16]. Also the post-cranial skeleton remains incompletely described, as Poplin and Mourer-Chauviré [12] mainly had fragmentary material available, so presacral vertebrae other than the atlas, axis and notarium, are virtually unknown, the humerus was poorly described, and major features of the leg bones including their actual size and proportions were unknown. These observations were augmented by Balouet with a simple skeletal reconstruction and some sketchy details, including that the pelvis had equally developed transverse processes and a large ilioischiadic foramen, the ribs lacked uncinate processes, the clavicles were unfused so there was no furcula, although the clavicle and coracoid were fused, and that there were a large number of synsacral and caudal vertebrae [14].

Knowledge of the biology of this bird also remains very limited. Mourer-Chauviré and Balouet [16] compared the skull of *S*. *neocaledoniae* to the giant gastornithids and dromornithids, the dodo and solitaire pigeons of the Mascarenes, and the moa-nalos of Hawaii, finding significant differences between it and these large herbivores. While noting the possibility that it fed on invertebrates they left open the question of the precise diet of *S*. *neocaledoniae*, other than that it was very specialised. Similarly, nothing is known about its breeding biology. But on the basis of the contemporary understanding that *S*. *neocaledoniae* was a megapode [11–13], several authors assumed it to be a mound builder and thus potentially responsible for constructing the enigmatic large mounds or tumuli on La Grande Terre and Ile des Pins (e.g., [18–20]). Megapodes are the only birds known to employ ectothermic incubation, that is do not brood their eggs and rely on environmental heat to incubate their eggs [21]. Whilst *S*. *neocaledoniae* was considered to be a megapode this idea had merit. If *Sylviornis* is no longer considered a crown or at least stem megapode, then mound building for egg incubation would be unlikely.

A second giant flightless galliform is known from Fiji. *Megavitiornis altirostris* Worthy, 2000 from Vitilevu in Fiji, is slightly smaller than *S*. *neocaledoniae* and was described as megapode [15]. Mourer-Chauviré and Balouet [16] considered the similarities between these two species, which include a remarkably similar craniofacial hinge and a tall, narrow rostrum, to be convergence. However, these morphological features are rare and apparently only distributed among galloanseres, being elsewhere only known in Dromornithidae and Gastornithidae [22–24], so any reappraisal of *S*. *neocaledoniae* thus has to also assess this aberrant Fijian galliform.

In this contribution, we therefore seek to address some of these knowledge gaps by describing the post-cranial skeleton in detail. We have a collection of 600 bones of *S*. *neocaledoniae* representing all skeletal elements made by some of us (THW, CS, AA) in caves on Pindai Peninsula, New Caledonia, in July 2003 [25]. Details of the sites and chronology of the deposits are in Anderson et al. [25]. The material reported previously [13, 14, 16] also derives from caves on Pindai Peninsula, but in the absence of any site descriptions by Balouet et al., it is not known whether it derived from one of the six caves surveyed by Anderson et al. [25], or another. The material described here all derives from separate sites in Cave B [25]. We use these specimens to interpret the morphology of *S*. *neocaledoniae* in a comprehensive phylogenetic analysis of both cranial and post cranial characters to establish the relationships of this strange bird, and thereby test the hypotheses that this species is (1) a galliform and (2) warrants its own family separate from megapodes. In doing so, such a phylogenetic analysis will shed light on whether *S*. *neocaledoniae* built mounds or used ectothermic incubation. We will further test this possibility within the context of the skeletal description by examining the functional capacity of *S*. *neocaledoniae* to actually undertake extensive digging as do megapodes. A cursory examination of some features, such as the unguals, shows that they differ greatly from those of megapodes that construct large mounds, e.g. *Megapodius* spp. Thus in the context of the skeletal description, we have paid particular attention to features that may impact on, or constrain, its digging ability, and compare it to mound—and non mound—building megapodes and chickens *Gallus gallus*.

## Materials and Methods

### Nomenclature

We follow the nomenclature and taxonomic order in Dickinson and Remsen [26]. Names for specific bone landmarks follow Baumel and Witmer [27] unless otherwise indicated. Anatomical landmarks are abbreviated in figure captions. Some common anatomical terms are abbreviated as follows: artic. (articularis); cond. (condylus); lig. (ligamentum); m. (musculus); proc. (processus).

### Fossil Material

The fossils of *S*. *neocaledoniae* reported here was collected by some of us (THW, CS, AA) in caves on Pindai Peninsula, New Caledonia, in July 2003 [25]. Details of the sites, their locations, excavations therein, and chronology of the deposits are given in Anderson et al. [25]. The material described here all derives from separate sites in Cave B at 21° 21' 005" S, 164° 57' 50.5" E [25]. The excavations were conducted with the authorisation of the Northern province President Paul Néaoutyine and the cultural authorities and the agreement of the customary authorities of the tribes of the Poya and Pouembout region. All the *Sylviornis neocaledoniae* material described below is part of the collections of Institut d'Archéologie de la Nouvelle-Calédonie et du Pacifique, Nouméa, New Caledonia. All IANCP catalogue numbers cited in this paper have the prefix ‘IANCP.PN/WNP011.PA/1.2003/’, which is abbreviated to ‘IANCP’ herein. The *Sylviornis* material is catalogued in the range 526–1088 as detailed in the descriptive section.

### Comparative Material

Palaeognathae: Fossil taxa. *Lithornis promiscuus* Houde, 1988 [28]–USNM 391983, 336535, 424072; *Lithornis plebius* Houde, 1988 [28]–USNM 336534 –holotype skeleton; *Paracathartes howardae* Harrison, 1979 [29]–USNM specimens: cranium– 361415; premaxilla– 404758; mandible– 361437–9, 404806; quadrate– 424067; palatine/pterygoid– 391984; vertebrae, C2–404756, C3–361428–9; 404747–8, 404906; 2L coracoids– 361416–417; LR scapula– 361418–9; 2R humerus– 361420, 361421; radius—L 361424, proximal parts– 361422–3, 361441; ulnae—R 361425, L 361426, R 361427; LR carpometacarpus– 361445–6; R femur– 361412; tibiotarsi—L 361407, R 361408, d+pL 361409, distal 361410, pR 361411, R 404749, dL 361409; 5 fibula– 361413–4, 361442, 361444, 404750; tarsometatarsus—R 361402, L 361403, L 361404, R 361405, L 361406, L 404747, L 404748, 361407, ungual phalanges– 404069, 404789, 404797; *Dinornis robustus* Owen, 1846 [30]–NMNZ S.163, 23342, 23654, 28225, 32667. Extant taxa: *Tinamus major* (= *robustus*)–SAM B.31339, USNM 347794, 621694; *Struthio camelus*–SAM B.10941, 11411, 31336; LACM99638; *Dromaius novaehollandiae*–SAM B.6863, 6898, 7068, 31580, 31581.

Fossil Galliformes: *Mwalau walterlinii* Worthy et al., 2015 [31]–see specimens listed in Worthy et al. [31]; *Megavitiornis altirostris* Worthy, 2000 –see NMNZ specimens listed in Worthy [15]; *Progura naracoortensis* van Tets, 1974 –SAM P16700, 17152–17154, 17856–17857, 17876–17879, 18181–18187, 36710–36716, 52473–52502; *Progura gallinacea* De Vis, 1888 –QM F1132, 1134, 1139, 1143, 5553, 5556–5558, 7005, 7033.

Extant Galliformes (alphabetical): *Acryllium vulturinum*–LACM88965F, 90645M, SAM B23933; *Aepypodius arfakianus*–ANWC O26042; *Aepypodius bruijnii*–USNM146767; *Alectura lathami*–SAM B46568, NMV B2209, B4288, B11471, B19290, B23648, B23649, B23650, QM O27218, QM O27843, QM O27844, QM O27852; *Coturnix pectoralis*–SAM B49460; *Crax rubra*–LACM113548, 101626; USNM 288713, 19918; *Eulipoa wallacei*–USNM 558275; *Gallus gallus*–SAM B11484, B46451, NMV B6363, B12748, B25087, QM O29536; *Leipoa ocellata*–SAM B414, B1094, B5039, B11480, B11481, B11482, B47825, B48526, B48765, B49461, B51215, B55458, B55528, B58520, B58560; *Macrocephalon maleo*–AMNH 12013 (by photos taken 2000 by J. Palmer), NHMUK 1891.7.20.97, 1871.7.21.1; USNM 225130; *Megapodius eremita*–NMV B20648, (and B20641, B20642, B20647, B24000, B24947, B24948, B24949, B24950, B24951, B24952, B25389); *Megapodius reinwardt*–ANWC O22869; *Ortalis vetula*–KU13342, USNM 19632, 288721, 288722; *Talegalla fuscirostris–*KU 97007, ANWC O3669; *Talegalla jobiensis*–USNM 146744, ANWC O7567.

Fossil Anseriformes: *Presbyornis pervetus* Wetmore, 1926 [32]. Skull: USNM 299846, 618166, 618202. Premaxilla: USNM 510082, 299845 (6 nose slab). Mandible: USNM 299847, 618169, 618215. Quadrate: USNM 498770. Thoracic vertebrae: USNM 616555 (specimen on small slab), 618205, 618207. Sternum: USNM 618212, 618214. Scapula: USNM 616557–616560–4 specimens, 618223—1L. Coracoid: USNM 618183 –left, 616561–616564–4 sternal parts, 616565–616567–3 omal parts. Humerus—USNM 483163 cast L, USNM 616568 –pt R hum, 618204 –complete L on slab, 618180 –dL. Ulna—USNM 616569–616571—3pL, 616572—1pR, 616573 & 616574—2dR. Carpometacarpus—USNM 616168 –left on slab, 618226—1pL, 618227—1pR. Femur: USNM 618228 –complete R, 618229—1dL, 618230–618232—3dR, 618233–4—2pL, 618235—1pR. Tibiotarsus: USNM 483165 –cast R, 618192–618196—5dL, 618236—1dR. Tarsometatarsus: USNM 483166 cast, USNM 618175 –dR, 618176 –dR, 618177 –R, 618178 –pR (4 parts), 618213 –R; 618237 –proximal. Pelvis: USNM 618167 –R ilioischial complex external view on slab, 618172 –L ilioischial complex internal view on slab, 618198 –synsacrum. *Anatalavis oxfordi* Olson, 1999 –see Olson [33]; *Dromornis planei* Rich, 1979 [34]–see Murray and Megirian [23] and Murray and Vickers-Rich [24].

Extant Anseriformes: *Chauna torquata*–USNM 631124, 614549, 428074; *Anhima cornuta*–MV B.12574; *Anseranas semipalmata*–SAM B36790, B48035; *Dendrocygna eytoni*–SAM B45769; *Cereopsis novaehollandiae*–SAM B39638, 49165; *Anser caerulescens*–SAM B36868; *Malacorhynchus membranaceus*–SAM B39384, B39385, B39639; *Tadorna tadornoides*–SAM B.39583, 39872.

Neoaves: *Burhinus grallarius*–SAM B.49554, B.48793; *Porphyrio melanotus*–SAM B.49644; *Grus rubicunda*–SAM B.49462.

### Measurements

All measurements are made with dial callipers (TESA) and rounded to nearest 0.1 mm. Measurements are either as described in the text or, for the 56 variables used in a PCA, are as given in S1 File.

### Statistical analyses

Summary statistics for measurements were generated in Microsoft Excel. Principal Component Analyses (PCA) were conducted in PAST v3.08 [35] to investigate how the shape of the tarsometatarsus and phalanges varied among the compared species to facilitate a prediction of the digging capability of *Sylviornis neocaledoniae*. We assume that digging capability is a proxy for the potential to build mounds. We therefore assembled measurements from multiple individuals of a range of megapodes with varying mound-building ability, from those not known to build mound (e.g. *Macrocephalon maleo*) to those that build large mounds (e.g. *Megapodius* sp.) and compared these to data for *S*. *neocaledoniae* and *Gallus gallus* (S1 File). Because there is no individual of *Sylviornis neocaledoniae* to compare to these individuals, we constructed an average *S*. *neocaledoniae* ‘individual’ using mean data for all the variables from Tables 1–21. The taxa varied greatly in size, with *S*. *neocaledoniae* being many times larger than all of the extant megapodes, therefore initial PCA plots of untransformed data showed *S*. *neocaledoniae* widely separated on PC1 from all other taxa, which were tightly grouped. As the aim of the analysis was to assess how shape varied among these taxa regardless of size, we standardized the data to size of the bird by dividing all values by femur length. Femur length is tightly correlated with mass (e.g., [36, 37]), therefore this transformation will reduce the measurements to a function of bird size, while preserving differences in shape or relative proportions of the distal leg. Missing data was an issue, especially where rare species were represented by 1 to 3 specimens e.g., *Eulipoa wallacei*, *M*. *maleo* and *Megapodius reinwardt*, and many specimens tended to have the horny cover still on the unguals. We therefore restricted measurements to those most often available and only measured the proximal phalanges and unguals I.2 and III.4. Where missing data were unavoidable, we utilised the iterative imputation method in PAST v3.08 where missing values are at first replaced by their column average, then an initial PCA run is used to compute regression values for the missing data. This procedure is iterated until convergence, see Ilin and Raiko [38]. We added to the size-transformed data a series of ratios designed to capture the relative lengths of phalanges as a proportion of the tarsometatarsus and also a ratio of width and depth of the unguals at mid length to capture the varying degree of dorsoventral flattening evident in taxa. See S1 File for analysis details.

Mass estimates were made using femoral mid-shaft circumference, which for *S*. *neocaledoniae* is the least-shaft shaft circumference, and the algorithms proposed by both Campbell and Marcus [36] and Field et al. [37].

### Phylogenetic analyses

The primary purpose of the phylogenetic analysis employed here was to examine the relationships of *Sylviornis neocaledoniae*. This species was originally described as a ratite [10], but affinities with megapodes were quickly established and widely accepted [11–16] until Mourer-Chauviré and Balouet [16] established a monotypic Sylviornithidae for it. Data matrices have been compiled to examine the phylogenetic relationships of palaeognaths [39–40], anatids [41], galliforms [42–44], but none on their own are suitable to assess those of a potentially basal galloanserine taxon.

We developed a set of 285 characters (S2 File) derived from direct comparison of specimens, and from the literature [mainly from 39, 41, 42, 44–54]. As per Worthy and Scofield [39], we followed two guiding principles that: (1) the characters must as far as possible relate to a single morphological feature or complex that is putatively homologous across all ingroup and outgroup taxa; and (2) character state definition should capture the full range of variation across the taxa analysed. Following these principles and because our taxon sample differed from any previously used, we revised all characters, sometimes substantially from those of previous definitions. The characters used were those identified as potentially relevant to relationships within Galloanseres. Therefore, characters from, for example, Ericson [48] and Mayr and Clarke [49], with states that were constant for crown and stem galloanseres were not used. That Galloanseres is the sister group to Neoaves is well established using both morphological [48, 49, 55] and molecular data (e.g., [56–58]) and so examining that relationship was not the aim of the present work.

We assembled a taxon set that sampled palaeognaths and Neoaves as two successively closer outgroup taxa, and an ingroup encompassing a range of anseriform and galliform taxa (galloanseres) with a focus on more basal taxa, and including key fossils. Because of pervasive problems of homoplasy among ratite palaeognaths related to loss or marked reduction of wings and large size of leg bones, we included three species of lithornithids, which are volant Eocene palaeognaths. Their age (55–48 Ma old: [28]) means they are separated from the common ancestor of crown galloanserines by less branch length (and thus, potentially less morphological evolution) than are any extant palaeognaths or Neoaves. In the phylogenetically furthest (palaeognath) outgroup, we also included a tinamou, and three ratites (*Struthio camelus*, *Dromaius novaehollandiae* and *Dinornis robustus*).

The phylogenetically closest outgroup, and immediate sister group to galloanseres, included three Neoaves (*Burhinus grallarius* (Charadriiformes), and *Porphyrio melanotus* and *Grus rubicunda* (Gruiformes)), chosen for their semi-terrestrial habits to minimise morphological disparity with the galloanseres.

Within Galloanseres, we sampled 27 extant and fossil taxa. The following extant taxa were included: seven species in six genera of Megapodiidae, six other galliforms representing Numididae, Cracidae, and Phasianidae, *Anseranas semipalmata* for Anseranatidae, *Chauna torquata* and *Anhima cornuta* for Anhimidae, and *Cereopsis novaehollandiae*, *Anser caerulescens*, *Dendrocygna eytoni*, *Malacorhynchus membranaceus*, and *Tadorna tadornoides* in Anatidae. To these we added the following fossil taxa: *Sylviornis neocaledoniae*, the giant Fijian *Megavitiornis altirostris*, described by Worthy [15] as a megapode, an extinct megapode from Vanuatu (*Mwalau walterlinii*; [31]), the Eocene anseriform *Presbyornis pervetus* (see [48, 59]), the late Cretaceous *Vegavis iaai* (see [60]), and the Australian dromornithid *Dromornis planei*, which is currently considered an anseriform (see [23, 24]). All character scoring was by direct examination of specimens listed in Comparative Material, except for *V*. *iaai*, which was scored from published descriptions [60, 61]. *Anatalavis oxfordi* was initially also included, scored from Olson [33] and photographs provided by Gareth Dyke, but this taxon acted as a wildcard greatly reducing tree resolution and support, and was therefore excluded.

The taxon-character matrix was analysed with parsimony using PAUP*4.0b10 [62] and Bayesian inference using MrBayes 3.2.5 [63]. The executable data matrix with PAUP and MrBayes commands is appended as S3 File. Trees were rooted between palaeognaths and all other sampled taxa. Both parsimony and Bayesian analyses were performed (1) with multistate characters ordered if they formed morphoclines (see S2 File), or (2) all unordered. The states for 60 characters marked with an asterisk in S2 File were identified as forming morphoclines and so were treated as ordered in some analyses.

With the full taxon set of 27 ingroup and 10 outgroup taxa, the Neoaves taxa and the lithornithids and tinamou were attracted to the stem of the anseriform lineage, inconsistent with the well-supported monophyly of palaeognaths, and the known sister group relationship of Neoaves with galloanseres (e.g., [56–58]). This was not unexpected because of two reasons: 1, the known problems with homoplasy within large flightless ratites [39–40] that was exacerbated by much missing data (e.g. pectoral girdle elements not scorable for many characters); and 2, that the dataset was mainly constructed to differentiate galloanseres, with (as noted above) exclusion of characters invariant among galloanseres, which would potentially help resolve ingroup-outgroup relationships. We consider that a separate characters set is required to tease out higher relationships of birds from those designed to address those of specific groups, as exemplified by Ericson [48] and Clarke et al. [60]. Rather than construct an entirely new analysis and dataset aimed at resolving ingroup-outgroup relationships, we performed the analyses with (1) a molecular backbone enforcing relationships between extant taxa supported by genetic studies and (2) constraints enforcing the well-corroborated ingroup and outgroup relationships discussed above, e.g., (palaeognaths ((Neoaves) (galloanseres)).

The molecular backbone employed below constrains taxa with molecular data (all living taxa plus moa *Dinornis*) to relationships supported by this genetic data, with fossils (*Mwalau walterlinii*, *Dromornis planei*, *Sylviornis neocaledoniae*, *Vegavis iaai*, *Presbyornis pervetus*, and the three lithornithid species) free to move within this backbone to their optimal positions based on morphological data. This approach ensures fossil taxa are placed within a phylogenetic framework of living taxa which is robustly supported by (often large) amounts of available molecular evidence. The following studies were used to construct the molecular backbone: Aves and Neoaves [55–58], Palaeognathae [40–64], Galliformes [65], and Anseriformes [66–68].

Parsimony analyses treated all changes as equal ("unweighted") and used heuristic searches with tree-bisection-reconnection (TBR) branch swapping and other default settings, and 1000 random addition replicates per search. When calculating tree lengths, multistate taxa were treated as polymorphisms rather than ambiguity. Gaps were treated as missing data. Strict consensus trees were computed from the set of most parsimonious trees, and clade support was assessed by bootstrapping [69] using the same settings and 1000 replicates. To prevent the bootstrap analyses from getting stuck on replicates with huge numbers of equally-parsimonious trees, nchuck was set to 2000.

Bayesian analyses used the Markov model of morphological evolution [70] as implemented in MrBayes [62]. Bayes Factors (twice difference in marginal log_n_ likelihoods) as calculated using stepping-stone analyses, strongly favoured including the gamma parameter for accommodating rate variation across characters (BF = 65.48 for the analyses with ordered characters and molecular backbone). The ‘coding = inf’ command was used to correct for under-sampling of invariant and autapomorphic characters. Strength of support was assessed using posterior probability.

## Results

### Description of the post-cranial skeleton of *Sylviornis neocaledoniae*

#### Vertebrae

**Fig 1:**
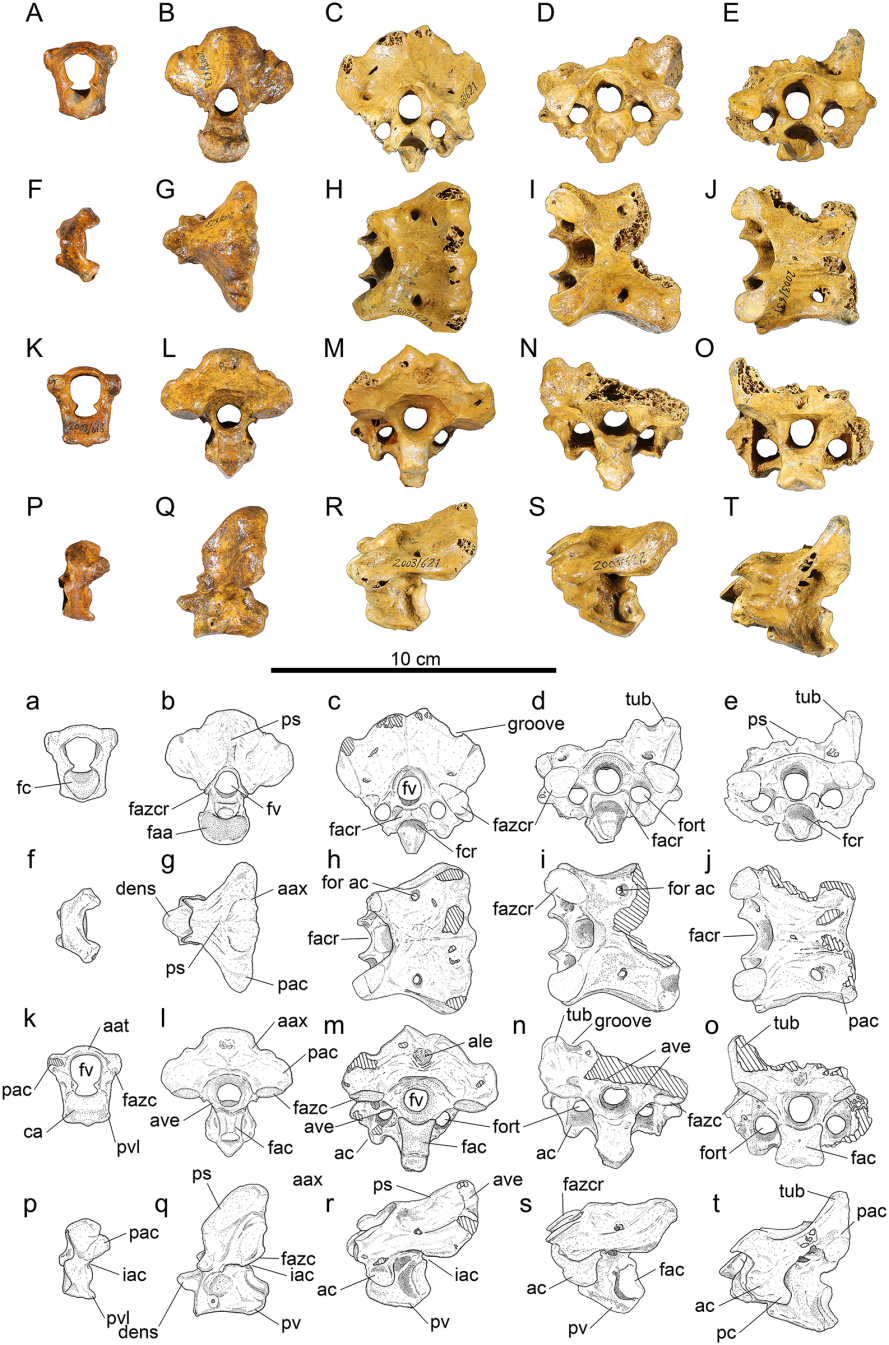
Vertebrae 1–5 of *Sylviornis neocaledoniae*. (A-E, a-e) anterior, (F-J, f-j) dorsal, (K-O, k-o) posterior, and (P-T, p-t) left lateral views. Photographs (A-T) and interpretive drawings (a-t): Vertebra 1, atlas, IANCP618 (A, F, K, P); vertebra 2, atlas, IANCP623 (B, G, L, Q); vertebra 3, IANCP621 (C, H, M, R); vertebra 4, IANCP622 (D, I, N, S); vertebra 5, IANCP631 (E, J, O, T). Abbreviations: aat, arcus atlantis; aax, arcus axialis; ac, ansa costotransversaria; ale, area lig. elastici; ave, arcus vertebrae; ca, corpus atlantis; faa, facies articularis atlantica; fac, facies artic. caudalis; facr, facies artic. cranialis; fazc, facies artic. zygapophysis caudalis; fazcr, facies artic. zygapophysis cranialis; fc, fossa condyloidea; fcr, fovea cranioventralis; for ac, foramen arcocostalis cranialis; fort, foramen transversarium; fv, foramen vertebrale; iac, incisura arcus caudalis; pac, proc. artic. caudalis; pc, proc. costales; ps, proc. spinosus; pv, proc. ventralis; pvl, proc. ventrolaterale; tub, tuberosity. Cross-hatching is missing bone.

*Atlas*, (Fig 1A, 1F, 1K, 1P, 1a, 1f, 1k and 1p):

Material: IANCP617, complete; IANCP618 (Fig 1), complete; IANCP619, near complete, missing the right processus artic. caudalis.

Measurements (mm) in order of specimens listed, with a dash for unmeasurable: length midline of centrum, 7.0, 6.8, 10.9; width across zygapophysis caudalis, 24.8, 25.7, -; maximum width fossa condyloidea, 13.0, 12.7, 12.0; maximum width facies artic. axialis, 17.3, 16.7, 16.2; height at mid-width facies artic. axialis, 10.6, 10.6, 12.3; width foramen vertebrale, 12.0, 10.9, 12.7; maximum height, 25.2, 28.3, 29.6.

The atlas was briefly described by Poplin and Mourer-Chauviré [12]. It is relatively short, being about three times taller than it is craniocaudally long, although the arcus atlantis is only little expanded dorsally over the width of the corpus atlantis (Fig 1a and 1k). The corpus atlantis has subparallel sides in its caudal section that frame the near-circular fossa condyloidea in cranial aspect (Fig 1k). The incisura fossae is broad, but the foramen vertebrale is relatively narrow, being only slightly broader than the fossa condyloidea. The atlas lacks foramina transversaria. The processus artic. caudales are robust, lateromedially thicker than they are caudally projecting, and are rounded caudally in lateral view (Fig 1f and 1p). The facies artic. zygapophyses caudales are distinct, yet small at 5 mm long by 4 mm wide (Fig 1k). The incisura arcus caudalis is shallow (Fig 1p). There is no distinct processus ventralis medially, but in IANCP618, there is a small median point caudally and in IANCP619 the processus ventrolaterales are connected medially creating a slight ventral projection. There is a pair of processus ventrolaterales that are caudoventrally directed and project less caudally than they are wide; they are usually separated by a shallow notch medially (Fig 1k and 1p).

*Axis*, (Fig 1B, 1G, 1L, 1Q, 1b, 1g, 1l and 1q): Material: IANCP620, complete; IANCP623 (Fig 1), complete; IANCP624, near complete, worn cranially. Of these, IANCP620 is of appropriate size and similar preservation to INACP619 (atlas) and the following cervicals, 3 (IANCP621), 4 (IANCP622), and 5 (IANCP631), are likely to be from one individual.

Measurements (mm) in order of specimens listed: Length midline of centrum excluding dens, 28.3, 25.6, -; width across zygapophysis caudalis, 47.5, 44.8, 42.0; maximum width facies artic. atlantica, estimated at 16.0, 17.0, -; maximum width facies artic. caudalis (at dorsal side), 11.4, 8.7, 10.1; height facies artic. caudalis including processus ventralis, 19.8, 16.2, 15.3; total maximum height, 49.8, 46.0, 45.4.

The axis was briefly described and figured by Poplin and Mourer-Chauviré [12]. It is dorsoventrally deeper than it is wide and is characterised by a massive dorsoventrally thickened arcus axialis connecting the processus artic. caudalis (Fig 1b and 1l). The corpus vertebra is caudally narrower than it is deep, lacks both a fovea cranioventralis and a fovea caudoventralis, and laterally has a rounded prominence at mid-depth just caudal to the cranial margin (Fig 1l). The facies articularis atlantica is broader than deep, and shallowly concave (Fig 1b). The dens is broader than deep and narrows cranially (Fig 1g). The facies articularis caudalis is heterocoelus and deeper than wide, but is directed dorsally (Fig 1l and 1q). The processus spinosus is low, forming a robust crest cranially, but merges caudally with a greatly thickened caudal side to the arcus axialis, such that in caudal view the arcus axialis and processus artic. caudalis form an even curve (Fig 1g and 1q). There are no foramina transversaria and processus costales are absent. The foramen vertebrale is laterally compressed cranially, but circular in caudal view. The facies artic. zygapophyses craniales are gracile projections on the arcus vertebrae at mid-depth of the foramen vertebrale and are about 5 mm long by 4 mm high in IANCP623 (Fig 1b and 1q). The facies artic. zygapophyses caudales are near circular facets about 10 mm in width that are directed ventrally from the robust processus artic. caudalis (Fig 1l and 1q). The processus artic. caudalis have a distinct groove aligned craniocaudally on their caudal margin (Fig 1b and 1l). The caudal facies of the arcus axialis dorsal to the facies articularis zygapophyses caudales is near planar and at right angles to the ventral side of the corpus (Fig 1l and 1q). The arcus axialis is narrow where it joins the corpus vertebrae, but widens markedly dorsally. There is a deep incisura arcus caudalis but no incisura arcus cranialis below the zygapophyses cranialis (Fig 1q). The processus ventralis is broad and robust, with greatest projection at the caudal end of the corpus, but it is of variable depth in the three specimens (Fig 1q). The arcus axialis has pneumatic foramina penetrating it cranially at the junction with the corpus as noted by was briefly described by Poplin and Mourer-Chauviré [12], but these are variably present even on each side of an individual, e.g. IANCP623 has a large foramen on the right side but none on the left (Fig 1q).

*Anterior cervicals*, *vertebra #3*, (Fig 1C, 1H, 1M, 1R, 1c, 1h, 1m and 1r): Material: IANCP621 (Fig 1), complete; IANCP625, incomplete, missing part of the left side.

Measurements (mm) in order of specimens listed: Length centrum from cranial-most point to the ventral side of facies artic. caudalis, 31.7, 31.0; width across zygapophysis cranialis, -, 38.5; width across zygapophysis caudalis, 52.1, 46.1; maximum width facies artic. cranialis, 13.7, 12.0; maximum width facies artic. caudalis, 12.7, -; height facies artic. caudalis to ventral side processus ventralis, 15.9, -; maximum height taken parallel to foramen vertebrale, 46.0, -.

Vertebra three, as best exemplified by IANCP621, is wider than high, and in dorsal view widens caudally. The corpus vertebra has a marked fovea cranioventralis for reception of the upturned facies artic. caudalis of the axis vertebra (Fig 1c). It is lateromedially compressed. The facies articularis cranialis is much broader than deep and directed cranioventrally (Fig 1c). The facies artic. caudalis is heterocoelous and directed slightly dorsally from the plane of the foramen vertebrale. The processus spinosus is indistinct and, as for the axis, the arcus vertebrae is very robust and arches between the processus artic. zygapophyses caudales forming a broad and deep near planar caudal facies above them (Fig 1m and 1r). In the centre of this planar caudal facies is a small (9 mm high, 7 mm wide) area ligamenti elastici for the insertion of ligaments linking to vertebrae 4 (Fig 1m). There are strongly enclosed foramina transversaria about 10 mm in length (Fig 1m). The ansa costotransversaria bears a short ventral processus but lacks a processus costalis, so is rounded caudally in lateral view (Fig 1r). Dorsally at mid-length there is a small circular foramen arcocostalis cranialis, which passes through to the caudal side of the facies articularis cranialis (Fig 1h). The foramen vertebrale is slightly lateromedially compressed in cranial view but circular in caudal view. The facies artic. zygapophyses craniales are slightly longer (11–12 mm) than wide (8–10.5 mm), and pointed mediocranially, although their margins are near circular elsewhere (Fig 1c). The facies artic. zygapophyses caudales are near circular and wider than long (Fig 1m). The arcus vertebra, like for the axis, is narrow at the junction with the corpus and broadens dorsally. The incisura arcus caudalis is shallower than in the axis (Fig 1r). The processus ventralis is broad and robust, largest caudally, and extends ventrally a distance equivalent to a third of the depth of the facies artic. caudalis (Fig 1m and 1r). There are small pneumatic foramina penetrating the arcus vertebrae within the foramina transversaria, and variably present larger ones penetrating the caudal side of the ansa costotransversaria.

*Anterior cervical*, *vertebra 4*, (Fig 1D, 1I, 1N, 1S, 1d, 1i, 1n and 1s): Material: IANCP622 (Fig 1), incomplete, missing right processus artic. caudalis and processus spinosus; IANCP626, incomplete, missing left processus artic. cranialis; IANCP627, incomplete, missing left processus artic. cranialis, processus spinosus, and the caudal margin of arcus vertebrae.

Measurements (mm) in order of specimens listed: Length centrum from cranial-most point to ventral side of facies artic. caudalis, 35.4, 35.0, 30.7; width across zygapophysis cranialis, 45.3, -, -; width across zygapophysis caudalis, -, estimated at 49.0, -; maximum width facies artic. cranialis, 14.4, -, -; maximum width facies artic. caudalis, 14.4, 13.7, 10.7; height facies artic. caudalis to ventral side processus ventralis, 18.6, 17.7, -; total height processus spinosus to processus ventralis, -, 46.6, -.

Descriptions are given in so far as vertebra 4 (IANCP 622) differs from vertebra 3 (IANCP621) supplemented by observations from the other specimens. Vertebra 4 is slightly larger than vertebra 3 and of similar form. Dorsally, the groove passing dorsally over the processus artic. caudalis, first noted on the atlas, is now markedly deepened, and lateral to it a distinct rounded tuberosity about 8 mm in diameter and 5 mm high is present (Fig 1d). The processus spinosus (IANCP626) is distinctly bifid on the dorsocaudal margin, about 13 mm wide and 4 mm high. On the caudal facies of this processus, the area ligamenti elastici is more marked and inset than in vertebra 3. The height of the arcus vertebra where it forms a broad flattened area between the facies artic. zygapophyses caudales and above the foramen vertebrale is lower. Laterally, the ansa costotransversaria is relatively larger than in vertebra 3, so that the foramen transversarium is longer, but the ventral processus on the ansa remains similarly short and robust, and there is no processus costales (Fig 1s). The processus ventralis is small. Vertebra 4 has pneumatic foramina in the same areas as vertebra 3.

*Anterior cervicals*, *vertebra 5*, (Fig 1E, 1J, 1O, 1T, 1e, 1j, 1o and 1t): Material: IANCP628, near complete, missing right processus artic. caudalis; IANCP629, incomplete, missing right zygapophysis cranialis and ventrocaudal part of corpus; IANCP630, near complete, missing right side arcus vertebra; IANCP631 (Fig 1), near complete, missing part right side processus artic. caudalis.

Measurements (mm) in order of specimens listed: Length centrum from cranial-most point to ventral side of facies artic. caudalis, -, -, 38.8, 37.6; width across zygapophysis cranialis, 49.2, -, -, 45.9; width across processus artic. caudalis, -, estimated at 48.0, -, -; maximum width facies artic. cranialis, 15.0, -, 14.7, 17.2; maximum dorsal width facies artic. caudalis, -, -, 14.0, 15.2; height facies artic. caudalis to ventral side processus ventralis, -, -, 17.0, 15.0; total height processus spinosus to processus ventralis, -, -, 41.2, 38.6; maximum height foramen vertebrale, 9.8, 9.5, 9.0, 10.4.

Descriptions are given in so far as vertebra 5 (IANCP 631) differs from vertebra 4 of the same individual (IANCP622), supplemented by observations from the other specimens. Vertebra 5 is the first in the vertebral series where the width cranially is greater than it is caudally across the processus artic. caudales. The broad flattened caudal facies forming the arcus vertebrae above the facies artic. zygapophyses caudales in preceding vertebrae is absent, but the dorsal projections on the processus artic. caudales are more greatly developed, rising about 13 mm above the adjacent facies (Fig 1e and 1o). The processus spinosus is bifid and low with the area ligamenti elastici more marked and deeply inset than in vertebra 4 (Fig 1e and 1o). The foramen arcocostalis cranialis is larger and circular (Fig 1j). Laterally, the ansa costotransversaria is relatively larger, the ventral processus is less prominent, and there is a processus costalis for the first time in the vertebral series (Fig 1t). As for vertebra 4, there is a deep fovea cranioventralis for the reception of the broad caudally flattened processus ventrale of the preceding vertebra (Fig 1e). In IANCP631 there is no processus ventralis, although in IANCP630 there is a slight projection. The facies articularis caudalis is wider than deep in IANCP631 and broadest ventrally (Fig 1o), whereas the preceding vertebrae are more lateromedially compressed. The arcus vertebrae is pneumatised in the area between the ansa costotransversaria and the facies artic. zygapophysis caudalis.

**Fig 2:**
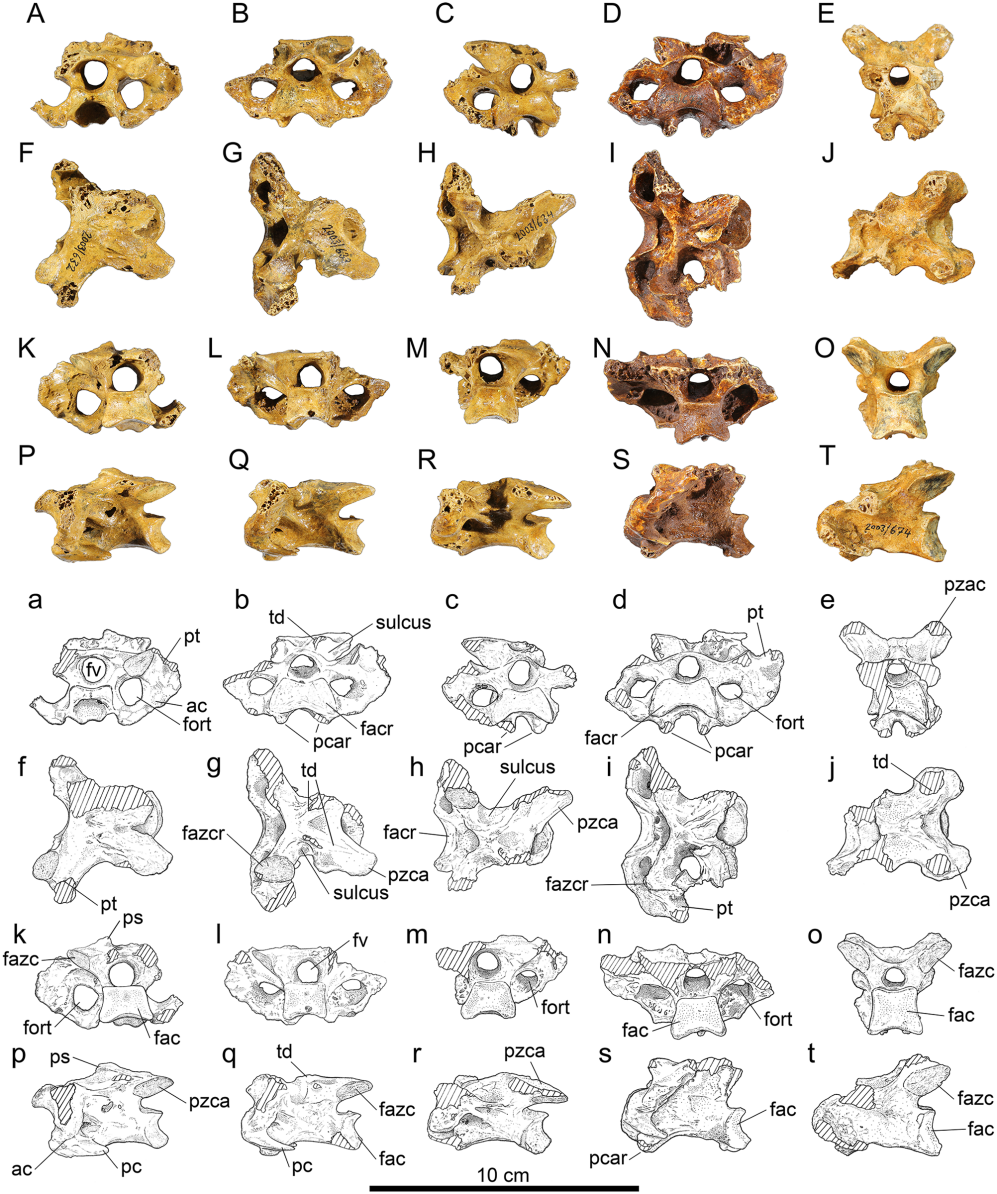
Vertebrae 6–10 of *Sylviornis neocaledoniae*. (A-E) anterior, (F-J) dorsal, (K-O) posterior, and (P-T) left lateral views. Vertebra 6, IANCP632 (A,F,K,P); vertebra 7, IANCP633 (B,G,L,Q); vertebra 8, IANCP634 (C,H,M,R); vertebra 9, IANCP672 (D,I,N,S) with fragment of a rib adhering dorsally; vertebra 10, IANCP674 (E,J,O,T). Abbreviations: ac, ansa costotransversaria; fac, facies artic. caudalis; facr, facies artic. cranialis; fazc, facies artic. zygapophysis caudalis; fazcr, facies artic. zygapophysis cranialis; fort, foramen transversarium; fv, foramen vertebrale; pc, proc. costales; pcar, proc. caroticus; ps, proc. spinosus; pt, proc. transversus; pzca, proc. zygapophysis caudalis; td, torus dorsalis. Cross-hatching is missing bone.

*Anterior cervicals*, *vertebra 6*, (Fig 2A, 2F, 2K, 2P, 2a, 2f, 2k and 2p): Material: IANCP632 (Fig 2), incomplete, missing right ansa costotransversaria and processus transversus; IANCP666, incomplete, missing the right ansa costotransversaria and the processus transversus and the ventral half of the corpus; IANCP677, incomplete, missing much of the arcus vertebrae.

IANCP632 has similar preservation and articulates well with IANCP631 (vertebra 5) and so descriptions are given in so far it differs from it, supplemented by the other specimen.

Measurements (mm) in order of specimens listed: Length centrum from cranial-most point to ventral side of facies artic. caudalis, 40.5, -, 40.3; width across zygapophysis cranialis, -, 42.7, -; maximum width facies artic. cranialis, 16.8, -, -; maximum width facies artic. caudalis, 19.1, -, -; height facies artic. caudalis, 13.1, -, 13.1; maximum width across processus transversus estimated from surviving half, 62, -, -; maximum height foramen vertebrale, 9.8, 9.2, 9.2.

Vertebra 6 is the first in the vertebral series with a processus transversus (Fig 2a and 2f): it is craniocaudally compressed and so deeper than long and projects laterad of the zygapophysis cranialis a distance equivalent to the width of the zygapophysis. Dorsally, the processus spinosus are further reduced compared to on vertebra 5 and are located more cranially at mid-length on the dorsal part of the arcus vertebrae and become torus dorsalis (Fig 2k and 2p). The foramen arcocostalis cranialis is absent, a marked difference from vertebra 5. The three examples of vertebra 6 are each eroded dorsally on the processus artic. caudales precluding knowing the extent of the projections that are large in vertebra 5. However, vertebra 6 is the first where distinct zygopophyses caudales project from the processus artic. caudales enclosing a deep U-shaped notch between them (Fig 2f and 2p). In IANCP666, this notch is broader and deeper than in IANCP632, but they are otherwise similar, especially in that they share a relatively broad flat area caudal to the zygapophyses cranialis, which leads to the processus transversus. This flattened area is absent in vertebra 7. Laterally, the ansa costotransversaria is craniocaudally shorter than it is in vertebra 5, not prominent ventrally and the processus costalis is well developed (Fig 2p). The foramen transversarium is, for the first time in the vertebral series, of greater diameter than the foramen vertebrale (Fig 2k). There is no processus ventralis.

*Anterior cervicals*, *vertebra 7*, (Fig 2B, 2G, 2L, 2Q, 2b, 2g, 2l and 2q): Material: IANCP633 (Fig 2), near complete, missing right zygapophysis caudalis and worn on each processus lateralis; IANCP673, incomplete, missing left zygapophysis cranialis.

Measurements (mm) in order of specimens listed: Length centrum from cranial-most point to ventral side of facies artic. caudalis, 42.0, 42.8; width across zygapophysis cranialis, -, 40.1; width across processus transversus, estimated at 62.0, -; maximum width facies artic. cranialis, 23.5, -; maximum width facies artic. caudalis, 17.0, -; height facies artic. caudalis, 13.2, 13.0; maximum height foramen vertebrale, 7.8, 9.4.

Vertebra 7 (IANCP633) articulates well with IANCP632 (vertebra 6). It is described in so far as it differs from the latter. Dorsally, the torus dorsalis aligns with the axis of the zygapophysis caudalis (Fig 2g). A deeply excavated dorsolaterally-open sulcus extends from the torus dorsalis to the margin of the facies artic. zygapophysis cranialis, contrasting markedly with vertebra 6 where this area is near flat (Fig 2b). The processus transversus is sloped more in the craniodorsal to caudoventral plane. Ventrally, for the first time in the vertebral series, there are two processus caroticus separated by about 14 mm (Fig 2b). Laterally, the ansa costotransversaria has no ventral prominence and the processus costalis is well developed and pointed (Fig 2q). There is no processus ventralis. The corpus vertebrae is pneumatised within the foramen transversarium.

*Anterior cervicals*, *vertebra 8*, (Fig 2C, 2H, 2M, 2R, 2c, 2h, 2m and 2r): Material: IANCP634 (Fig 2), near complete, missing left zygapophysis caudalis, and left ansa costotransversaria.

Measurements (mm): Length centrum from cranial-most point to ventral side of facies artic. caudalis 44.1, maximum width facies artic cranialis 22.0, maximum width facies artic. caudalis 19.4, height facies artic. caudalis 13.6, maximum width across processus transversus estimated from preserved half 56.0, maximum height foramen vertebrale 9.6.

Vertebra 8 differs from vertebra 6 in that the sulcus between the torus dorsalis and the facies artic. zygapophysis cranialis is more broadly open. In caudal view, a fovea cranioventralis is present again (lacking in vertebra 7), and the processus carotici are more narrowly separated. Pneumatic foramina penetrate the corpus vertebrae and the ansa costotransversaria within the foramen transversarium.

*Posterior cervicals*, *vertebra 9*, (Fig 2D, 2I, 2N, 2S, 2d, 2i, 2n and 2s): Material: IANCP635, near complete, missing right zygapophysis caudalis and the caudal half of the corpus; IANCP672 (Fig 2), near complete, missing the ends of the zygapophysis caudales.

Measurements (mm) in order of specimens listed: Length centrum from cranial-most point to ventral side of facies artic. caudalis -, 48.3; width across zygapophysis cranialis 42.8, -; maximum width facies artic. cranialis 23.8, 23.8; maximum width facies artic. caudalis -, 19.6; height facies artic caudalis -, 14.9; maximum width across processus transversus estimated from best half 70.0, 66.0; maximum height foramen vertebrale 8.9, 8.6.

Vertebra 9 differs from vertebra 8 by more enlarged processus transversus (Fig 2d), the sulcus dorsally, being even more broad (Fig 2i), and the processus caroticus being farther apart, enclosing a deep U-shape (Fig 2d), and in cranial view the dorsal side of the articular facies is more deeply U-shaped.

*Posterior cervicals*, *vertebra 10*, (Fig 2E, 2J, 2O, 2T, 2e, 2j, 2o and 2t): Material: IANCP661, fragment, missing left zygapophysis cranialis and right zygapophysis caudalis and processus transversi; IANCP674 (Fig 2), near complete, missing both zygapophyses cranialis and processus transversi.

Measurements (mm) in order of specimens listed: Length centrum from cranial-most point to ventral side of facies artic. caudalis 49.1, 48.8; width across zygapophysis caudalis, -, 41.8; maximum width facies artic. caudalis 19.7, 20.0; height facies artic. caudalis 17.9, 17.1; maximum height foramen vertebrale 9.0, 8.1.

Vertebra 10 is poorly represented, so the form and extent of the processus transversus is unknown. It differs from vertebra 9 by absence of the deep and broad sulcus between the torus dorsalis and the facies artic. zygapophysis cranialis (Fig 2j). The torus is more rounded and located farther caudally. The facies artic. zygapophysis cranialis, judging by IANCP661, is relatively broader. The facies artic. caudalis is larger, and deeper than wide, rather than wider than deep (Fig 2o). The processus caroticus are narrowly separated and ventrally enclose a deep fovea cranioventralis (Fig 2e). The arcus vertebrae and the corpus are not pneumatised.

**Fig 3:**
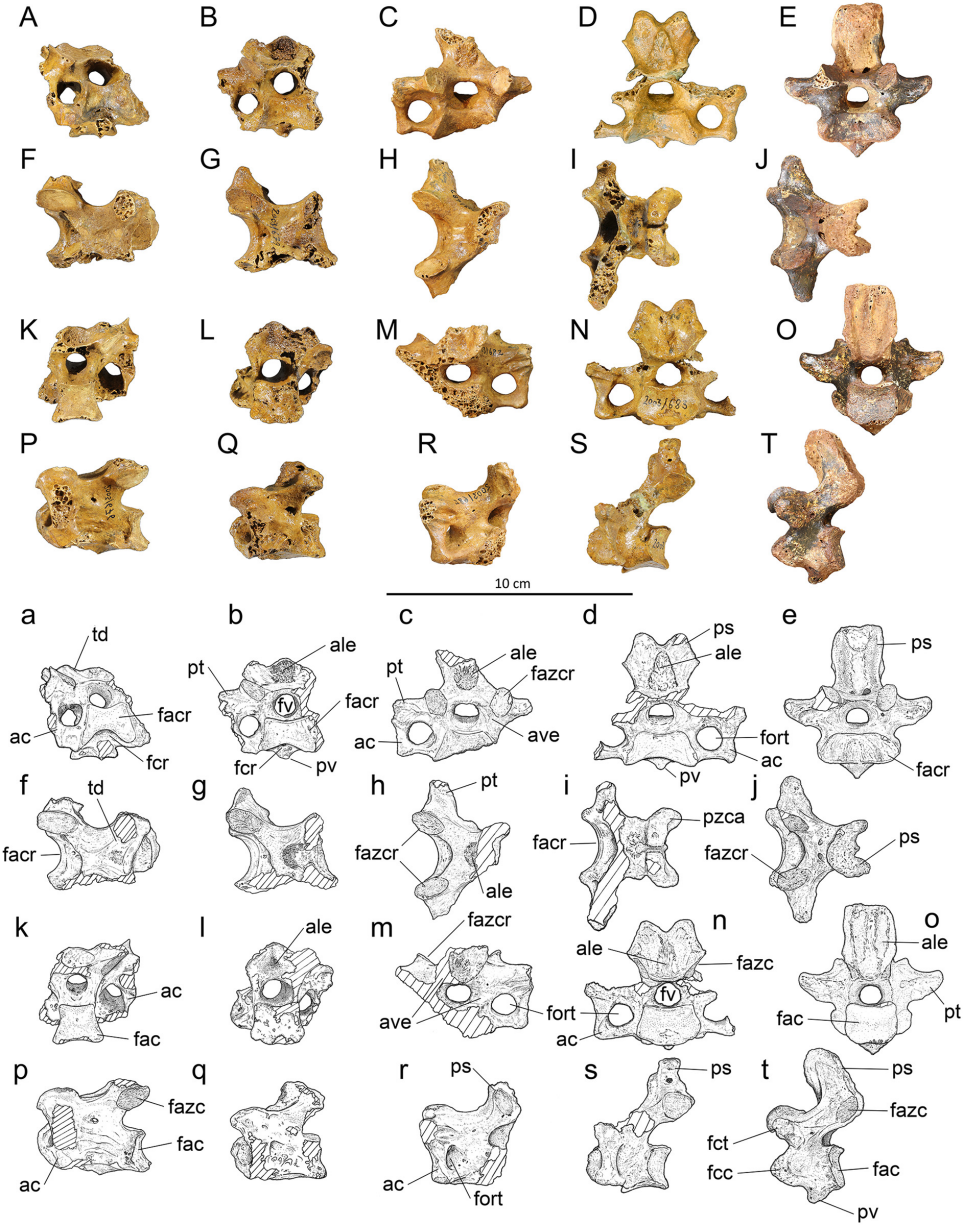
Vertebrae 11–15 of *Sylviornis neocaledoniae*. (A-E) anterior, (F-J) dorsal, (K-O) posterior, and (P-T) left lateral views. Vertebra 11, IANCP676 (A,F,K,P mirrored); vertebra 12, IANCP679 (B,G,L,Q); vertebra 13, IANCP682 (C,H,M,R mirrored); vertebra 14, IANCP683 (D,I,N,S); vertebra 15, IANCP691 (E,J,O,T). Abbreviations: ac, ansa costotransversaria; ale, area lig. elastici; ave, arcus vertebrae; fac, facies artic. caudalis; facr, facies artic. cranialis; fazc, facies artic. zygapophysis caudalis; fazcr, facies artic. zygapophysis cranialis; fcc, fovea costalis capituli; fcr, fovea cranioventralis; fct, fovea costalis tuberculi; fort, foramen transversarium; fv, foramen vertebrale; ps, proc. spinosus; pt, proc. transversus; pv, proc. ventralis; pzca, proc. zygapophysis caudalis.

*Posterior cervicals*, *vertebra 11*, (Fig 3A, 3F, 3K, 3P, 3a, 3f, 3k and 3p): Material: IANCP663, partial, missing left side corpus, left zygapophysis cranialis and both processus transversi; IANCP667, partial, missing left zygapophysis cranialis and left zygapophysis caudalis; IANCP676 (Fig 3), partial, damaged as per last; IANCP678, fragment, retains only right zygapophysis caudalis. IANCP675, partial, is smaller than the others listed here and subadult in its ossification, but is clearly the same vertebra.

Measurements (mm) in order of specimens listed, adults only: Length centrum from cranial-most point to ventral side of facies artic. caudalis, 48.8, 44.5, 46.3, 45.7; width across zygapophysis caudalis, 40.2, -, -, -; maximum width facies artic. cranialis, -, -, 20.6,-; maximum width facies artic. caudalis (ventral side), -, 20.5, 19.5, 17.5; height facies artic. caudalis, 17.5, 17.3, 15.8, 15.7; maximum height foramen vertebrale, 9.8, 8.8, 8.5, 8.9.

Vertebra 11 is very similar to vertebra 10 and differs as follows: the torus dorsalis are linked by a more elevated ridge that bounds a fovea caudodorsalis between the zygapophyses caudales (Fig 3f). The arcus vertebrae is thin and dorsally flattened above the foramen vertebrae cranially. Where this flat area rises caudally to form the ridge linking the zygapophyses caudales, it has a rugose area centrally, which is an area ligamenti elastici marking the most cranial insertions of the major ligaments associated with the thoracic vertebrae. Ventrally, the processus carotici are replaced by a centrally located processus ventralis that extends over the cranial half of the centrum from a shallow fovea cranioventralis (Fig 3a). The centrum is distinctly compressed at midlength compared to the facies articularis caudalis. The arcus vertebra is pneumatised within the foramen transversaria.

*Posterior cervical*, *vertebra 12*, (Fig 3B, 3G, 3L, 3Q, 3b, 3g, 3l and 3q): Material: IANCP679 (Fig 3), partial, missing the left zygapophysis cranialis and its associated ansa costotransversaria; IANCP671, partial, corpus vertebra only.

Measurements (mm) in order of specimens listed: Length centrum from cranial-most point to ventral side of facies artic. caudalis 38.4, 35.5; maximum width facies artic. cranialis 26.0, 25.0; maximum width facies artic. caudalis 21.5, -; width at mid-length centrum 18.1, estimated at 21.0; height facies artic. caudalis 16.2, 17.4; maximum height foramen vertebrale 9.3, -.

Vertebra 12 is distinguished from vertebra 11 by its abruptly shorter centrum which is also comparatively wider at mid-length (Fig 3q). Dorsally, the area ligamenti elastici is enlarged from that in vertebra 11, forming a slightly prominent rugose area 9 by 9 mm in area (Fig 3l). There is a small cranially pointed processus ventralis just caudal to the fovea cranioventralis (Fig 3b). An incipient processus transversus projects from the ansa costotransversaria (Fig 3b).

*Posterior cervical*, *vertebra 13*, (Fig 3C, 3H, 3M, 3R, 3c, 3h, 3m and 3r): Material: IANCP681, partial, subadult missing, most of processus spinosus and processus transversi; IANCP682 (Fig 3), partial, missing part corpus vertebra and left ansa costotransversaria and processus transversus.

Measurements (mm) IANCP682 only: Length centrum from cranial-most point to ventral side of facies artic. caudalis 30.9; width across zygapophysis cranialis 35.0; maximum width facies artic. cranialis estimated at 29.0; maximum width across processus transversus estimated from complete side 60.0; maximum height foramen vertebrale 9.2.

Vertebra 13 is the first of the cervicothoracic vertebrae, i.e. those with distinct processus spinosus but no articulations for ribs, and is described based on the more complete example IANCP682. It articulates well with IANCP679. The processus spinosus is eroded dorsally so its dorsal extent is unknown, but in cranial view its width expands dorsally, and it was wider than long (Fig 3c). The area ligamenti elastici cranially is a rugose area about 9 mm wide that is not prominent (Fig 3c). The corpus vertebra is relatively shorter and broader than it is in vertebra 12 and is dorsoventrally compressed. The foramen vertebrale is circular and about the same size as the foramen transversarium (Fig 3m). The ansa costotransversaria projects horizontally from the corpus vertebra, then extends dorsally forming a right angle around the foramen transversarium to link to the processus transversus (Fig 3c). The processus transversus is short and only slightly prominent laterally of the ansa costotransversaria. There are single small pneumatic foramina penetrating the arcus vertebrae cranially from within the foramen transversarium.

*Posterior cervical*, *vertebra 14*, (Fig 3D, 3I, 3N, 3S, 3d, 3i, 3n and 3s): Material: IANCP683 (Fig 3), partial, missing right ansa costotransversaria and processus transversus; IANCP684, near complete, missing left processus transversus and zygapophysis cranialis.

Measurements (mm): Length centrum from cranial-most point to ventral side of facies artic. caudalis 30.9, 29.5; width across zygapophysis caudalis 31.3, 25.6; maximum width facies artic. cranialis estimated at 29.5, 25.6; maximum width facies artic. caudalis 24.7, estimated at 24.7; height facies artic. caudalis 15.8, 15.4; maximum width across processus transversus estimated from complete side 63.0, 64.0; maximum height foramen vertebrale 9.4, 8.5.

Vertebra 14 is the second of the cervicothoracic vertebrae, and is described based on the more complete example IANCP683 relative to vertebra 13. It has a well-developed bifid processus spinosus that projects dorsally (c. 12 mm), slightly higher than the height of the facies artic. zygapophysis caudalis, and is wider (25 mm) than it is long (12 mm) (Fig 3d). The specimen IANCP684 has a slightly higher processus spinosus. The area ligamenti elastici cranially is a prominent rugose area 15 mm high by 10 mm wide (Fig 3d). The vertebra has a wide but shallow fovea cranioventralis with a small processus ventralis projecting cranially below the fovea (Fig 3d). The foramen vertebrale is circular and about the same size as the foramen transversarium. As in vertebra 13, the ansa costotransversaria projects horizontally from the corpus vertebra, then extends dorsally forming a right angle around the foramen transversarium to link to the processus transversus. The processus transversus is short and only slightly prominent laterally of the ansa costotransversaria (Fig 3n). There are single small pneumatic foramina penetrating the arcus vertebrae cranially from within the foramen transversarium.

*First thoracic*, *vertebra 15*, (Fig 3E, 3J, 3O, 3T, 3e, 3j, 3o and 3t): Material: IANCP691 (Fig 3), complete.

Measurements (mm): Length midline of centrum 19.7; width across zygapophysis cranialis, 31.3; width across zygapophysis caudalis, 22.0; maximum width facies artic. cranialis, 32.6; maximum width facies artic. caudalis, estimated at 21.5; height at mid-width facies artic. caudalis, 16.1; maximum width across processus transversus, estimated from surviving half, 60.2; dorsoventral diameter foramen vertebrale, 8.0.

Vertebra 15 is the first thoracic vertebrae as evidenced by presence of fovea costalis tuberculi et capituli (Fig 3t). It differs from vertebra 14 with a taller non-bifid processus spinosus that extends dorsally a distance 1.5 times the diameter of the facies artic. zygapophysis caudalis (Fig 3e). The processus spinosus is however still wider than it is craniocaudally long (Fig 3j). The size of area ligament elastici is larger and much more protuberant cranially than in vertebra 14. The foramen vertebrale is circular and reduced in diameter from that of vertebra 14 (Fig 3o): all more caudal vertebrae have smaller diameter foramina. The facies artic. zygapophyses caudales are more narrowly divergent dorsally than they are in vertebra 14 and do not project laterally of processus spinosus (Fig 3o and 3t). There is no foramen transversarium. A small, cranially located processus ventralis is present (Fig 3t). The fovea costalis tuberculi (upper rib attachment) is located on the ventral side of processus transversus and the fovea costalis capituli (lower attachment) on the lateral side of the corpus just caudal to the lip of the facies artic. cranialis (Fig 3t). The vertebra is not pneumatised.

**Fig 4:**
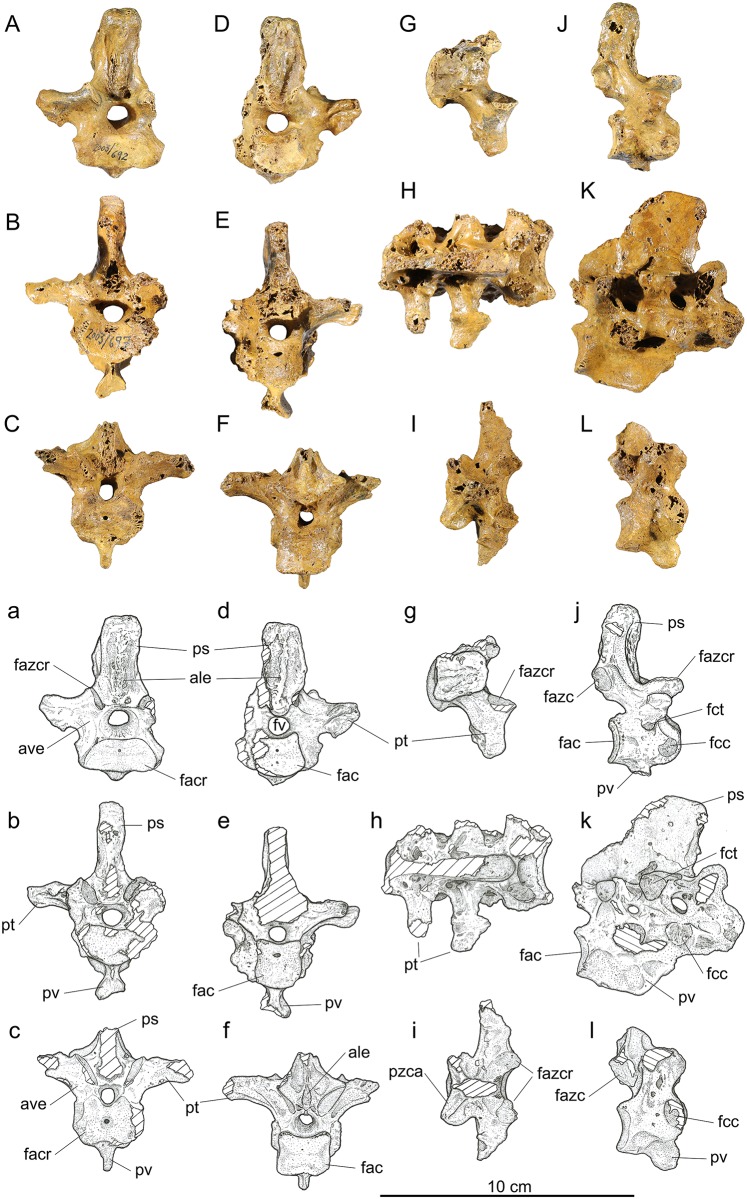
Vertebrae 16–20 of *Sylviornis neocaledoniae*. (A-C) Anterior, (D-F) posterior, (G-I) dorsal, and (J-L) right lateral views. Vertebra 16, IANCP692 (A,D,G,J); notarium, vertebrae 17–19, IANCP697 (B,E,H,K); vertebra 20, IANCP690 (C,F,I,L). Abbreviations: ale, area lig. elastici; ave, arcus vertebrae; fac, facies artic. caudalis; facr, facies artic. cranialis; fazc, facies artic. zygapophysis caudalis; fazcr, facies artic. zygapophysis cranialis; fcc, fovea costalis capituli; fct, fovea costalis tuberculi; fv, foramen vertebrale; ps, proc. spinosus; pt, proc. transversus; pv, proc. ventralis; pzca, proc. zygapophysis caudalis.

*Second thoracic*, *vertebra 16*, (Fig 4A, 4D, 4G, 4J, 4a, 4d, 4g and 4j): Material: IANCP680, near complete, worn to tips of processus spinosus, processus transversi, and lateral margins of facies artic. cranialis; IANCP685, centrum only; IANCP692 (Fig 4), near complete, missing only the left processus transversus.

Measurements (mm) in order of specimens listed: Length centrum from cranial-most point to ventral side of facies artic. caudalis 28.8, 30.4, 29.2; Length midline of centrum, 21.7, 23.5, 21.0; width across zygapophysis cranialis, 35.0,-, estimated at 25.0; width across zygapophysis caudalis, 21.4, -, 19.8; maximum width facies artic. cranialis, 36.0, 34.5, 32.4; maximum width facies artic. caudalis, 24.4, 22.7, estimated at 23.0; height at mid-width facies artic. caudalis, 16.4, 15.3, 15.5; dorsoventral diameter foramen vertebrale, 6.9, 7.6, 7.6.

While IANCP692 articulates reasonably well with vertebra 14 (IANCP684) and are likely to be of the same individual judging by size and shared preservation characteristics, the morphology of IANCP691 (attributed here to vertebrae 15) is a good intermediate and indicates that an intervening vertebra is missing. Vertebra 16 (IANCP692) is little different from that referred to vertebra 15 in its articular facies, but has a taller but narrower processus spinosus, larger foveae costales (Fig 4j), and a noticeably smaller diameter of the foramen vertebrale (Fig 4d). In addition, the facies artic. zygopophyses caudales are subparallel to each other (divergent dorsally in vertebra 15 and more so in 14) (Fig 4j). The area ligament elastici are much larger, matching the increased size of the processus spinosus, but form prominent medial crests both cranially and caudally (Fig 4a and 4d). The fovea costalis tuberculi is pedicellate and about 5 mm in diameter on the ventral surface of the processus transversus (Fig 4j). The foveae costales capituli are larger, near 7 mm diameter, on the craniolateral margin of the facies artic. cranialis (Fig 4j). There are no pneumatic foramina.

*Notarium*, *fused vertebrae 17–19*, (Fig 4B, 4E, 4H, 4K, 4b, 4e, 4h and 4k): Material: IANCP696, complete fused corpus vertebrae but lacking processus spinosus and processus transversi for all; IANCP697 (Fig 4), near complete, processus transversi broken off on left side and caudal part of processus spinosus lost; IANCP699, a caudal fragment preserving mainly vertebra 19.

Measurements (mm) in order of specimens listed: Length midline of centrum 63.0, 62.0, -; width across zygapophysis cranialis vertebra 17, estimated at 22.0, 24.2, -; width across zygapophysis caudalis vertebra 19, 25.3, 27.0; maximum width facies artic. cranialis, -, 26.0, -; maximum width facies artic. caudalis, 19.5, 18.0, 21.3; height at mid-width facies artic. caudalis, 16.1, 16.9, 16.7; dorsoventral diameter foramen vertebrale cranially, 7.1, 6.6, -; dorsoventral diameter foramen vertebrale caudally, 6.5, 6.1, 5.0; maximum width across processus transversus, -, estimated from preserved half 64.0, -; maximum height from dorsal side facies artic. zygapophysis caudalis to ventral side facies artic. caudalis, 35.0, 35.0, -; maximum height, -, 78.7, -.

The notarium is comprised of three fused thoracic vertebrae as described by Poplin and Mourer-Chauviré [12], and here is described in more detail primarily from the more complete IANCP697. The facies artic. cranialis is about twice as wide as deep, whereas the facies artic. caudalis is about as wide as it is deep (Fig 4b). The notarium lacks a fovea cranioventralis. Ventrally, a prominent processus ventralis forms a 12 mm wide oval plate that is cranioventrally directed and located beneath processus transversus of vertebra 18 and is linked by a crest to the ventral margin of facies artic. caudalis (Fig 4k). The cranial end of this plate is close to the corpus vertebra, but the ventral surface of vertebra 17 is broken on this and IANCP696 precluding knowing whether a crista ventralis adorns that vertebra ventrally. The foveae costales capituli are located at the cranial margin of each component vertebra and are located dorsally such that they overlap the foramen vertebrale (Fig 4k). IANCP697 is the only specimen preserving processus transversi and then only for vertebrae 18 and 19. The form of the processus transversus on vertebra 17 is, judged from that on vertebra 16 which articulates well with this notarium specimen, to be more robust and extend further laterally than does that on vertebra 18. Those on vertebrae 18 and 19 are successively shorter and that on vertebra 18 shows that there were no ossified ligaments linking them laterally (Fig 4h), and so the notarium lacked fenestra intertransversaria. The processus spinosus, not preserved in the material available to Poplin and Mourer-Chauviré [12], was at least 43 mm long near the arcus vertebrae, thicker cranially (10.7 mm) than it is most caudally (4.3 mm), and extended at least 31 mm above the processus transversi (Fig 4k). Two foramina intervertebrale penetrate the arcus vertebrae providing exits for the pelvic nerves from the foramen vertebrale (Fig 4k). Pneumatism of the notarium is restricted to recessi dorsocraniales pneumatici on the two posterior vertebrae.

*Vertebra 20*, *last presacral vertebra*, (Fig 4C, 4F, 4I, 4L, 4c, 4f, 4i and 4l): Material: IANCP687, near complete, missing left processus transversus and tip of processus spinosus; IANCP688, fragment; IANCP689, near complete, missing tips processus transversi and tip processus spinosus; IANCP690 (Fig 4), near complete, missing right processus transversus and processus spinosus; IANCP694, juvenile, near complete.

Measurements (mm) for near complete adult specimens in order listed: Length midline of centrum 21.0, 24.0, 23.0; width across zygapophysis cranialis 29.0, 27.2, -; width across zygapophysis caudalis, -, estimated at 30.0, -; maximum width facies artic. cranialis, 27.8, 27.8, 27.6; maximum width facies artic. caudalis, -, 20.9, 21.8; height at mid-width facies artic. caudalis, 14.5, 16.5, 14.3; maximum width across processus transversus, -, estimated at 68.0, -.

Vertebra 20 has a short corpus vertebrae that is considerably wider than deep cranially but only slightly wider than deep caudally (Fig 4c and 4f). A processus ventralis is variably expressed, as it is well developed cranially in IANCP689 and the juvenile IANCP693, but smaller in IANCP690 (Fig 4c). A processus ventralis is predicted given it is robust on some notaria, e.g. IANCP 697, but the single synsacrum available (IANCP559) has only a low crista ventralis cranially, so the size/presence of a process on vertebra 20 may be variable/absent. The fovea costalis capituli is large and on the cranial margin of the corpus at a level just below the foramen vertebrale (Fig 4l). A feature of vertebrae 20, and as conforms with the notarium and the synsacrum, is the narrow width of the processus spinosus compared to that of preceding vertebrae. Another is the narrow diameter of the foramen vertebrale (Fig 4c), which is less than in all prior vertebrae. The corpus vertebra lacks pneumatic foramina but foramina, possibly nutrient ones, penetrate the arcus vertebrae below the processus transversus.

#### Pelvis (Fig 5)

**Fig 5:**
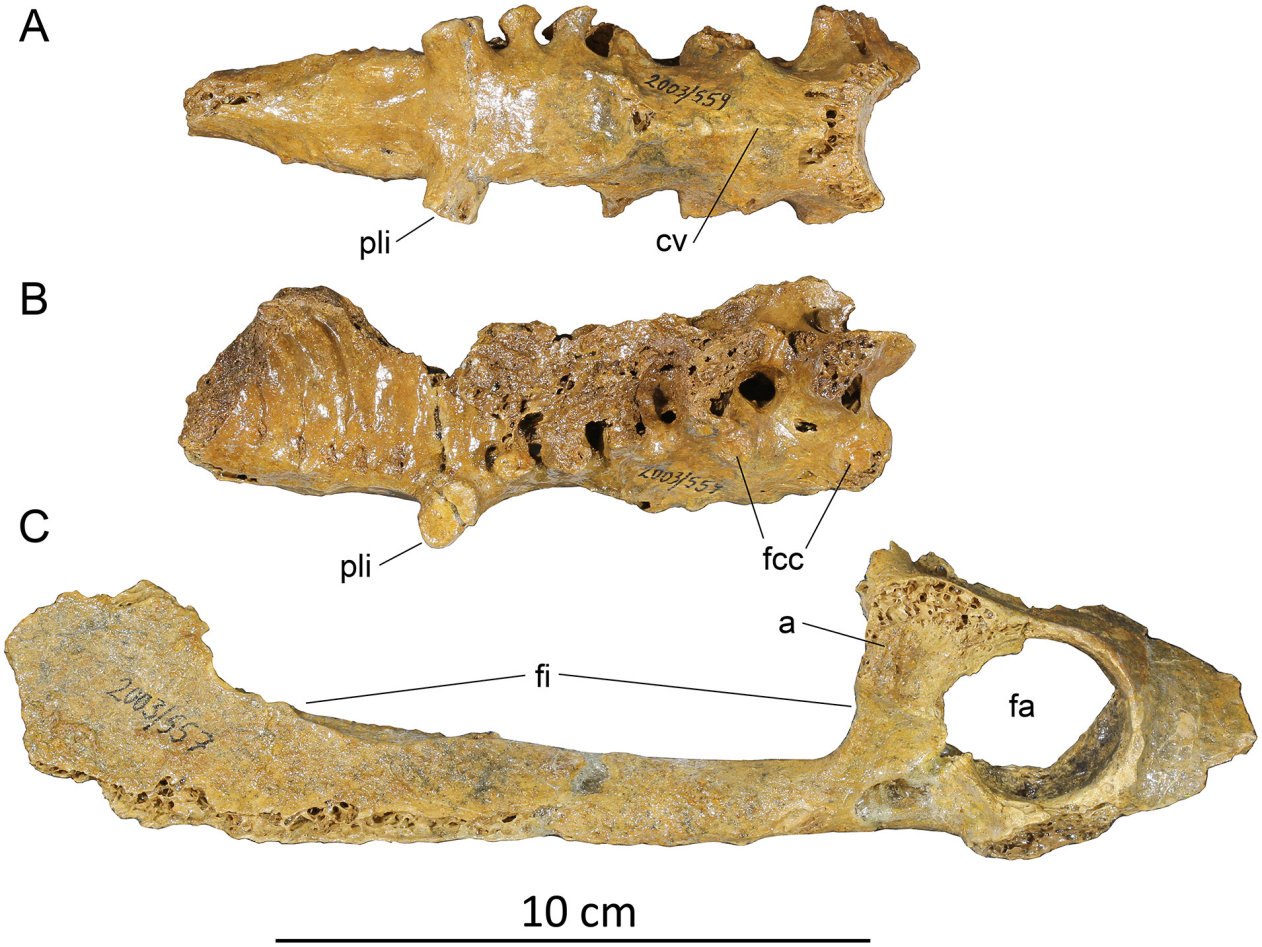
Pelvis of *Sylviornis neocaledoniae*. (A, B) synsacral fragment (IANCP559), (C) right acetabulum and ischium (IANCP557). Abbreviations: a, antitrochanter; cv, crista ventralis; fa, foramen acetabuli; fcc, foveae costales capituli; fi, foramen ilioischiadicum; pli, processus lateralis for articulation with ilium.

Material: IANCP559, a synsacrum, entirely lacking the processus spinosus and processus transversi on the anterior three vertebrae. Measurements: preserved length 117 mm long, with 12 fused vertebrae. Length anterior to the largest iliac articulation 79 mm, maximum diameter across the iliac articulation 34 mm. IANCP558, the caudal half of the left ilium synsacrum including the antitrochanter. IANCP557, the right ischium and part ilium preserving the acetabulum: preserved length 210 mm; dorsoventral height of foramen acetabuli is 30 mm.

Synsacrum: The synsacrum includes six anterior vertebrae. The first three support a distinct crista ventralis that abruptly terminates under the third synsacral vertebrae: caudal to this point the ventral surface of the synsacrum is flattened over the next 6–7 vertebrae (Fig 5A). Vertebrae 1 and 2 have foveae costales capituli (Fig 5B). Neither the height of the processus spinosus nor the width of the processus transversi can be determined. Vertebrae 4–6 have flattened facets on short processus laterales that articulated with the ilium (Fig 5A). The most caudal processus is the largest with its facet 12.4 by 9.9 mm.

Ischium: The ischium is straight in the lateromedial plane, not bowed, and while eroded caudally apparently had a straight ventral surface. The foramen ilioischiadicum is elongate, c. 110 mm long and accounts for 76% of the preserved postacetabular length (Fig 5C). However, its length is 3.7 times the diameter of the foramen acetabuli, whereas in *Leipoa ocellata* SAM B11482 it is only 2.4 times as long, so it is relatively much more elongate in *S*. *neocaledoniae*, and clearly much longer than half ischial length. A crest terminating ventrally at the anterior end of this foramen marks the caudal end of the foramen obturatum, which is about 22 mm long and overlapped anteriorly the foramen acetabuli. The corpus ischii lacks pneumatic foramina either externally nor internally.

Ilium: The ilium fragment reveals little except that the fossa iliaca dorsalis extends caudally to the area dorsal of the antitrochanter.

#### Humerus (Figs 6A, 6B and 7A, 7B)

**Fig 6:**
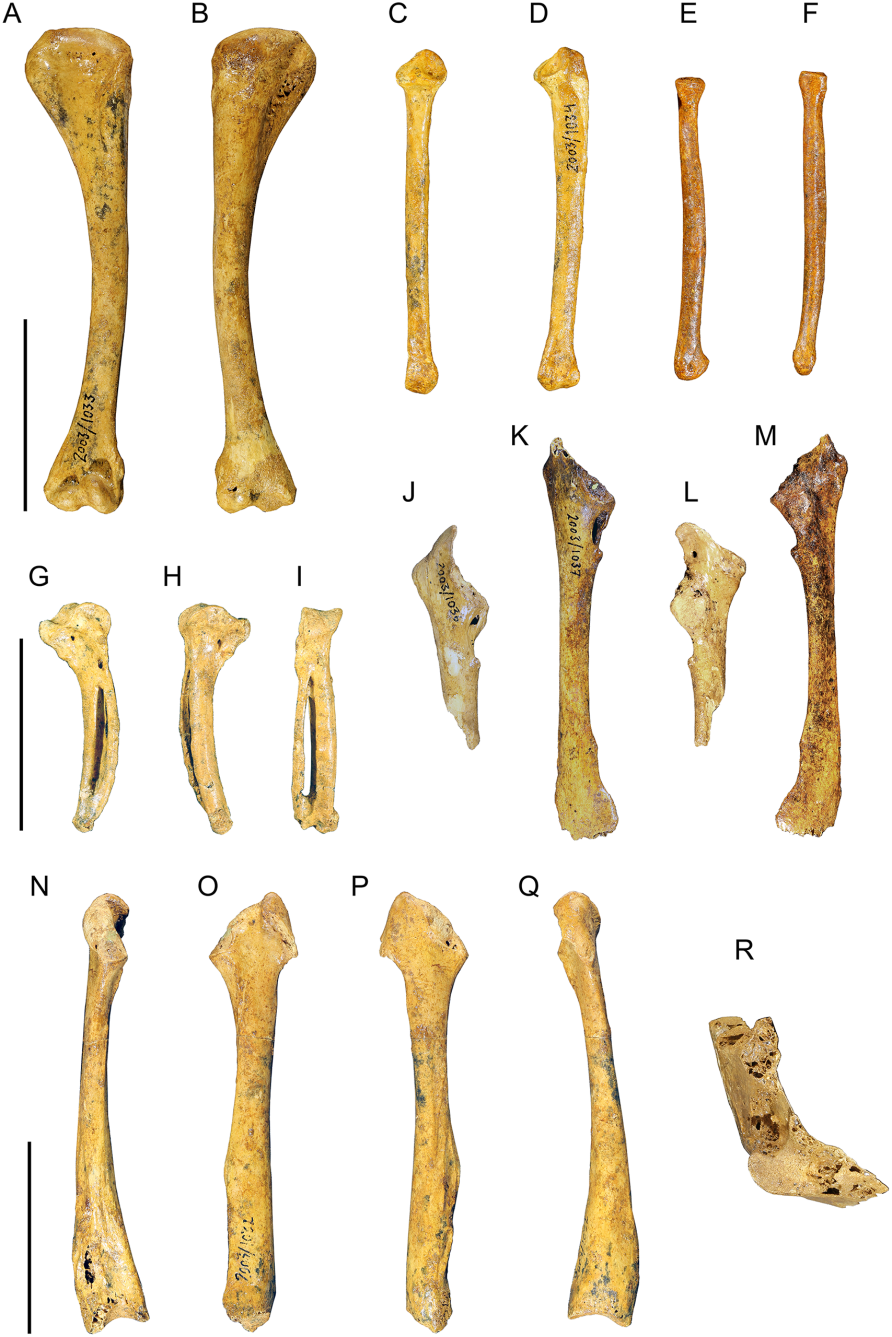
Photographs of pectoral girdle elements of *Sylviornis neocaledoniae*. Left humerus IANCP1033 in cranial (A) and caudal (B) views. Right ulna IANCP1034 in internal (C) and ventral (D) views. Right radius IANCP1041 in caudal (E) and dorsal (F) views. Right carpometacarpus IANCP1038 in ventral (G), dorsal (H) and caudal (I) views. Left scapulae IANCP1036 (J, L) and IANCP1037 (K, M) in medial (J, K) and lateral (L, M) views. Left coracoid IANCP1032 in dorsal (N), medial (O), lateral (P) and ventral (Q) views. Sternum IANCP1060 in anterior view (R). Scale bars are 5 cm.

**Fig 7:**
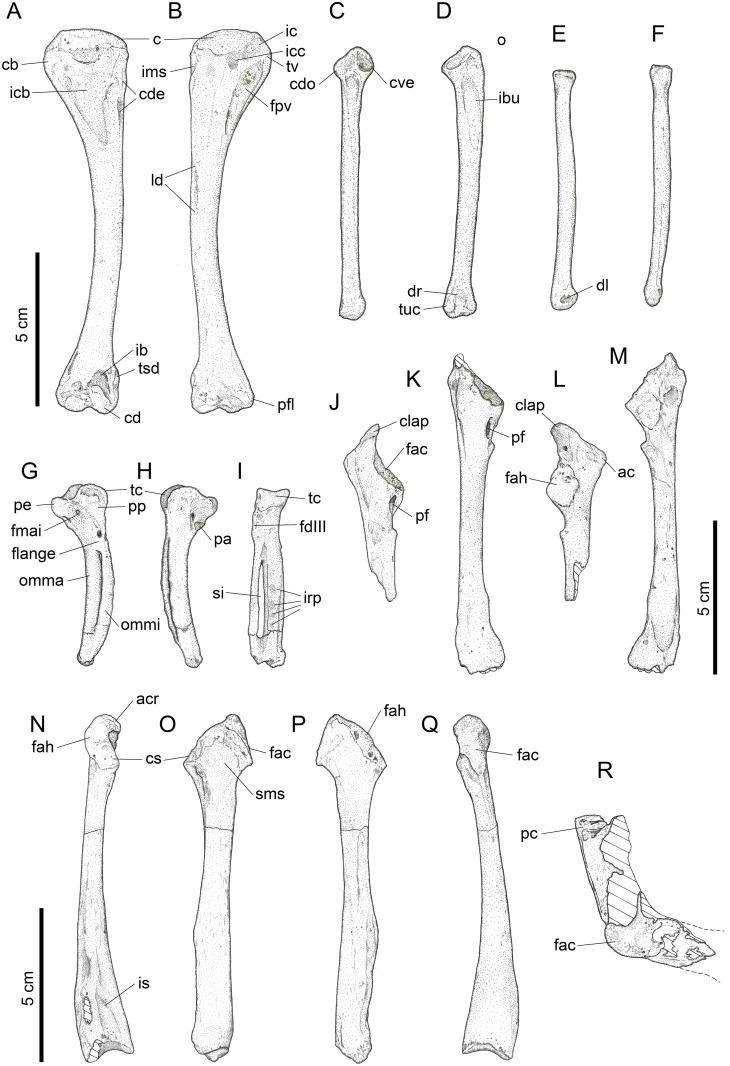
Interpretive drawings of pectoral girdle elements of *Sylviornis neocaledoniae*. Left humerus IANCP1033 in cranial (A) and caudal (B) views. Right ulna IANCP1034 in internal (C) and ventral (D) views. Right radius IANCP1041 in caudal (E) and dorsal (F) views. Right carpometacarpus IANCP1038 in ventral (G), dorsal (H) and caudal (I) views. Left scapulae IANCP1036 (J, L) and IANCP1037 (K, M) in medial (J, K) and lateral (L, M) views. Left coracoid IANCP1032 in dorsal (N), medial (O), lateral (P) and ventral (Q) views. Sternum IANCP1060 in anterior view (R). Abbreviations: ac, acromion; acr, acrocoracoid; c, caput; cb, crista bicipitalis; cd, condylus dorsalis; cde, crista deltopectoralis; cdo, cotyla dorsalis; clap, crista lig. acrocoraco-procoracoideum; cs, cotyla scapularis; cve, cotyla ventralis; dl, depressio ligamentosa; dr, depressio radialis; fac, facies artic. coracoideum; facs, facies artic. coracoideus on sternum; fah, facies artic. humeralis; fdIII, origin m. flexor digiti III; fmai, fossa m. abductor indicis; fpv, fossa pneumotricipitalis ventralis; ib, impressio brachialis; ibu, impressio brachialis ulnaris; ic, incisura capitis; icb, impressio coracobrachialis; icc, insertii m. coracobrachialis caudalis; ims, impressio m. supracoracoideus; irp, insertions remiges primarii; is, impressio sternocoracoidei; ld, attachment m. latissimus dorsalis; o, olecranon; omma, os metacarpale majus; ommi, os metacarpale minus; pc, processus costales; pe, processus extensorius; pf, pneumatic foramen; pfl, processus flexorius; pp, processus pisiformis; si, spatium intermetacarpale; sms, sulcus m. supracoracoideus; tc, trochlea carpalis; tsd, tuberculum supracondylaris dorsalis; tuc, tuberculum carpale; tv, tuberculum ventrale.

Material: IANCP1033, R Hum; IANCP1042, R Hum; IANCP1043, R Hum; IANCP1044, juv. R Hum; IANCP1045, pL Hum; IANCP1046, juv. pL Hum; IANCP1047, dR Hum; IANCP1048, juv sL Hum; IANCP1049, chick d+pR Hum; IANCP1050, pR Hum; IANCP1051, pR Hum; IANCP1052, pL Hum; IANCP1053, dL Hum; IANCP1054, sR Hum; IANCP1055, shaft Hum; IANCP1056, 2 dL Hum; IANCP1057, dR Hum; IANCP1058, pR Hum; IANCP1059, juv pL Hum; IANCP1062, pR Hum; IANCP1063, sR Hum; IANCP1064, pR Hum; IANCP1065, dR Hum; IANCP1066, L Hum.

Measurements: Table 1.

**Table 1:** Summary statistics of measurements (mm) for humeri of *Sylviornis neocaledoniae*.

	TL	PW	mid SW	DW
Mean	120.2	27.4	9.4	20.2
Standard Deviation	4.22	1.15	0.66	0.42
Minimum	114.2	25.7	8.6	19.7
Maximum	123.3	28.5	10.2	20.7
Count	4	8	4	8

Poplin and Mourer-Chauviré [12] had only poorly preserved fragments of this element and gave a minimal description which we greatly enlarge on. The humerus, as best exemplified by IANCP1033 and IANCP1042, is reduced in size compared to pelvic elements, with the proximal and distal ends poorly developed relative to that in volant megapodes and the shaft relatively thin for its length. The shaft is slightly arched dorsally but is straight in dorsal aspect. The crista deltopectoralis is reduced to a low rounded, ventrally convex, ridge that extends ventrally onto the cranial surface (Figs 6A and 7A). The crista bicipitalis is thick and terminates distally more proximally than does the crista deltopectoralis, and the insertion of m. scapulohumeralis caudalis (m. dorsalis scapulae) on its caudomedial margin is poorly defined. The fossa pneumotricipitalis ventralis is relatively small, very shallow, and pneumatic (Figs 6B and 7B). The crus dorsale fossa is low and rounded and extends proximally of the fossa pneumotricipitalis ventralis to a low rounded tuberculum ventrale. The incisura capitis is elongate and shallow and bound dorsally by the margo caudalis which links to the widest part of the caput humeri. The incisura has a few nutrient foramina opening from it into the caput humeri. The insertii m. coracobrachialis caudalis is located in the dorsal end of the incisura, but it is, however, only a shallow poorly defined sulcus (Figs 6B and 7B). The tuberculum intermedium [71], which characteristically for galliforms extends from the caput humeri to close the incisura capitis dorsally, is lacking in *Sylviornis*. Similarly, the fossa pneumotricipitalis dorsalis that is deep in many galliforms, is reduced to a shallow sulcus distal to the dorsal half of the caput. The impressio m. supracoracoideus is elongate and forms a shallow scar on the dorsal facies level with the fossa pneumotricipitalis and so separated distally from the dorsal margin of caput humeri where it usually inserts in taxa with a tuberculum dorsalis (Figs 6A and 7A). There is a small and low tuberculum dorsale in *Sylviornis* adjacent to the dorsal margin of the caput, but it is not protuberant. Cranially, there is a well-developed transversely broad impressio coracobrachialis centred on the proximal end in its widest part. The sulcus ligamentum transversus is a very shallow groove restricted to the crista bicipitalis just proximal to the tuberculum ventrale. The attachment of m. latissimus dorsi forms an elongate crest nearly 20 mm long on the dorsal side of the caudal shaft surface.

Distally, the humerus is unusual because the cranial surface proximal to the condyles is essentially flat and lacks a fossa brachialis. The impressio m. brachialis forms a very shallow elongate scar extending about 20 mm proximally from a point close to the medial margin level with the proximal side of condylus dorsalis (Figs 6A and 7A). The scar is bound medially by a narrow crest on the cranioventral margin of the shaft and extends dorsally to cover about 3/4 of the shaft width. There is no tuberculum supracondylare ventrale and only a very small flat scar for the insertion of pronator brevis ventrally. There is, however, a prominent, robust and rounded tuberculum supracondylaris dorsalis that ends distally at a level just proximal of condylus dorsalis (Figs 6A and 7A). The condyli dorsalis et ventralis are well-developed, but the processus flexorius ends distally proximad of a line drawn across the distal margin of the condyles. Caudally there is no sulcus scapulotricipitalis, but the sulcus humerotricipitalis is deep and leads to a shallow fossa olecrani.

#### Ulna (Figs 6C, 6D and 7C, 7D)

Material: IANCP1034, R ulna; IANCP1067, R ulna; IANCP1068, R ulna; IANCP1069, R ulna; IANCP1070, L ulna; IANCP1071, L ulna; IANCP1072, pL ulna; IANCP1073, pL ulna; IANCP1074, pL ulna; IANCP1075, pL ulna; IANCP1076, dL ulna; IANCP1077, dL ulna; IANCP1078, pL ulna; IANCP1079, juv pL ulna; IANCP1080, pL ulna; IANCP1081, juv L ulna; IANCP1089, pR ulna; IANCP1090, pR ulna; IANCP1091, dL ulna; IANCP1092, juv pR ulna.

Measurements: Table 2.

**Table 2:** Summary statistics of measurements (mm) for ulnae, radii and carpometacarpi (cmc) of *Sylviornis neocaledoniae*. Ulna DW is width across condylus dorsalis ulnaris, ulna SW is dorsoventral width at mid length.

	Ulna TL	Ulna PW	Ulna SW	Ulna DW	Radius TL	Radius DW	Cmc TL	Cmc PW
Mean	87.8	13.1	5.8	11.5	81.5	9.6	58.9	18.2
Standard Deviation	4.55	0.46	0.42	0.41	2.67	0.22	0.59	0.55
Minimum	80.0	12.5	5.4	11.0	78.5	9.4	58.2	17.5
Maximum	93.0	14.0	6.5	12.1	83.5	9.9	59.8	19.0
Count	6	9	5	7	3	4	6	5

Ulnae are much shorter than humeri but are variable in length (Table 2). Poplin and Mourer-Chauviré [12] had complete specimens of this element and described it briefly, but our material allows a more detailed description. The shaft is straight and compressed dorsoventrally, such that at the impressio brachialis it is much deeper craniocaudally than thick dorsoventrally. The impressio brachialis is shallow and flat and extends nearly to the caudal margin (Figs 6D and 7D). The cotyla dorsalis is prominent and its articular facet curves over distally to face cranially but does not form a hook. The incisura radialis varies from a shallow to a very deep fossa that undercuts the cotylae, e.g., IANCP1072. The cotyla ventralis is deeply concave to take the well rounded humeral condylus dorsalis. The olecranon is very poorly developed and does not project proximally of the cotyla ventralis. The margo caudalis bears very shallow pits that are the homologues of the papillae remigalis caudalis, best seem in IANCP1069 where 5 are visible in the distal two thirds of length. Distally, the condylus dorsalis ulnaris is flattened dorsally, and truncated distally not rounded. There is no incisura tendinosa and the tuberculum carpale is very short such that there is no incisura tuberculum carpalis. A distinct depressio radialis is, however, present (Figs 6D and 7D) and extends slightly proximal to the condylus dorsalis ulnaris.

#### Radius (Figs 6E, 6F and 7E, 7F)

Material: IANCP1035, L rad; IANCP1041, R rad; IANCP1082, R rad; IANCP1083, juv R rad; IANCP1084, juv L rad; IANCP1085, juv R rad; IANCP1086, dR rad; IANCP1087, pR rad; IANCP1088, pR rad.

Measurements: Table 2.

The radii are short and massive as described by Poplin and Mourer-Chauviré [12], with the shaft slightly bowed ventrally. Proximally, the cotyla humeralis is ovoid in proximal view and shallow. Distally, the sulcus tendinosus dorsally and the depressio ligamentosa ventrally are very shallow (Figs 6E and 7E).

#### Carpometacarpus (Figs 6G–6I and 7G–7I)

Material: IANCP1016, R cmc; IANCP1038, R cmc; IANCP1093, L cmc; IANCP1094, R cmc; IANCP1095, L cmc; IANCP1096, L cmc.

Measurements: Table 2.

Carpometacarpi range in length from 58.2 to 59.8 mm. They were described in some detail by Poplin and Mourer-Chauviré [12] but we expand on their descriptions here. The shaft of the carpometacarpus is bent cranially in its distal half (Figs 6G, 6H and 7G, 7H). Only IANCP1038 has a complete os metacarpale minus and it reveals that the spatium intermetacarpalis is parallel sided over most of its length, rather than markedly wider distally as in volant megapodes. The trochlea carpalis is robust and in ventral aspect has a round profile but in dorsal profile is somewhat flattened. The trochlea carpalis is distinctly grooved so that in caudal or cranial view it is notched proximally. There is no fovea carpalis cranialis and no fovea carpalis caudalis. The processus extensorius is robust and rounded, shorter than the ventral width of the trochlea carpalis, directed slightly proximally, and rotated ventrally at the tip. The processus alularis is well developed indicating the presence of well-formed pollex. The dorsal rim of the trochlea carpalis is offset markedly dorsally from the shaft, rather than more or less aligned with it. The processus pisiformis is very low and rounded, and the fossa infratrochlearis is very shallow. The ventral facies is excavated from the base of the processus extensorius caudally and distally towards the os metacarpale minus forming a broad flattened fossa confluent with the synostosial facies for the origin of m. abductor indicis [72] (Figs 6G and 7H). There is a large protuberant tuberosity ventrally on the proximal end of os metacarpale minor for the origin of m. flexor digiti III (Figs 6I and 7I). At the proximal end of the fossa for m. abductor indicis and about the middle of the base of the processus extensorius is a foramen that passes right through the bone to exit dorsally on the shaft margin and caudal and more proximal than processus alularis. The presence of the foramina is variable, as in IANCP1094 neither are present and in IANCP1096 the dorsal one is lacking. Like all megapodes, *S*. *neocaledoniae* lacks a processus intermetacarpalis, with the insertion of the m. extensor metacarpi ulnaris (flexor attachment) a low elongate scar on the caudal facies just distal to the proximal synostosis of the metacarpals. However, on the ventral facies, the os metacarpale minus and os metacarpale majus are linked by a flange of bone of variable length that delimits proximally a foramen through which a vessel ran in a proximoventral to distodorsal direction (Figs 6G and 7G). This flange has the effect of increasing the area of origin for m. abductor indicis. Distally, the synostosis of the ossa metacarpale minus and metacarpale majus is short. The os metacarpale minus has less distal extent than the os metacarpale majus. The os metacarpale majus has 4 large depressions on its dorsal surface for the insertion of remiges primarii, clearly visible in IANCP1038 (Figs 6I and 7I).

#### Os carpi ulnare

Material: IANCP1039, 2L ulnare. The ulnare is about the size of that of *Leipoa ocellata*, but the crus breve is relatively shorter and the incisura metacarpalis is a broader U-shape. The processus muscularis is poorly developed so that the proximoventral profile of the crus longum is rounded, not angular.

#### Scapula (Figs 6J–6M and 7J–7M; Table 3)

Material: IANCP1036, L scap; IANCP1037, L scap; IANCP1112, 2L1R juvenile scap; IANCP1113, R juvenile scap; IANCP1114, R scap; IANCP1115, L scap; IANCP1116, shaft R scap; IANCP1117, L scap; IANCP1118, juvenile R scap; IANCP1119, L scap; IANCP1120, R scap.

Our material is more complete than that available to Poplin and Mourer-Chauviré [12] whose specimens critically lacked the acromial portion and most of the blade (Table 3). The scapula as best exemplified by IANCP1036 and 1037 has a straight corpus that is not bowed laterally and with a flat dorsal profile from the collum to near the distal end. The depth of the corpus is least at mid-length (c. 8 mm) and increases gradually distally to an abrupt ventral expansion at the distal tip (Figs 6K, 6M and 7K, 7M), however no specimen is complete distally. The acromion forms a robust rounded protuberance on the dorsal margin cranial to the facies articularis humeralis that is both expanded dorsally and laterally (Figs 6L and 7L). Extending cranially from this is a large lateromedially compressed flange about 10 mm long and some 8 mm deep whose ventral border is a sharp crest which is interpreted as the crista ligamentum acrocoraco-procoracoideum (Figs 6J and 7J). The base of this process is pneumatic in IANCP1036, but not in the other three assessable specimens (IANCP1037, 1115, 1120). There is no tuberculum coracoideum as the coracoid lacks a cup-like cotyla scapularis, but there is instead a flat facies articularis coracoideum about 12 mm long by 8 mm wide (Figs 6J and 7J) that extends ventrally from the crista ligamentum acrocoraco-procoracoideum. This facies is rough and slightly porous and likely indicates a fairly immobile joint. In contrast, the facies articularis humeralis is a smooth slightly convex oval zone extending 10–12 mm caudally from the cranial margin adjacent to the ventral margin on the lateral facies (Figs 6L and 7L). This facies occupies about half of the dorsoventral depth of the scapula and is bound dorsally by a shallow fossa that extends caudad of the acromion. Medial to the facies articularis humeralis and opening ventrally or on the ventromedial surface is a large pneumatic foramen of variable size (Figs 6K and 7K), much larger than that found by Poplin and Mourer-Chauviré [12], which may reflect individual variation. Immediately caudal to the pneumatic foramen is a prominent tuberculum m. scapulotricipitis that is the cranial end of a more elongate scar for the insertion of the scapulotriceps muscle. On the lateral facies of the collum and dorsal to this tuberculum, there is a narrow linear crest that extends dorsocaudally interpreted as for the insertion of m. scapulohumeralis cranialis.

**Table 3:** Measurements of scapulae of *Sylviornis neocaledoniae*: A, acromion to cranioventral side of facies articularis humeralis; B, dorsoventral depth of collum at cranial side of tuberculum m. scapulotricipitis; C, length from cranial side of acromion to preserved distal end.

Specimen	A	B	C
IANCP1037	22.4	11.0	99.0^a^
IANCP1036	23.4	9.3	
IANCP1114	21.3	9.6	100.7^a^
IANCP1115	20.5	10.6	
IANCP1120	21.2	9.8	
IANCP1119	20.6	9.6	

^a^, both specimens are lacking the distal tip of the corpus, estimated in each case to be about 10 mm.

#### Coracoid (Figs 6N–6Q and 7N–7Q; Table 4)

Material: IANCP1032, L cor; IANCP1097, cran pt L cor; IANCP1098, juv. R cor; IANCP1099, R cor; IANCP1100, cran pt R cor; IANCP1101, cran pt L cor; IANCP1102, cran and sternal parts R cor; IANCP1103, cran pt R cor; IANCP1104, juv. R cor; IANCP1105, juv. sternal pt R cor; IANCP1106, juv. cran pt R cor; IANCP1107, cran pt L cor; IANCP1108, cran pt L cor; IANCP1109, cran pt L cor; IANCP1110, cran pt R cor; IANCP1111, sternal pt R cor; IANCP1121, sternal pt L cor.

Poplin and Mourer-Chauviré [12] had no complete examples of this bone and gave a brief descriptions of the omal end. The coracoid of *Sylviornis* is large and robust (Table 4), but relatively elongate compared to that of extant megapodes like *Alectura* and *Leipoa*. The shaft differs from extant megapodes in being slightly bowed laterally and is lateromedially compressed so is markedly dorsoventrally deeper than it is wide, even though the dorsal surface is concave from the cotyla scapularis to the beginning of the impressio sternocoracoidei, unlike in most galliforms where this region is a flat surface above which the cotyla barely protrudes (Figs 6N–6Q and 7N–7Q). This lateromedial compression is marked at the omal end through the cotyla scapularis and facies articularis humeralis where depth is about three times width (Figs 6O and 7O). This compression is exacerbated by a very reduced acrocoracoid which projects only slightly ventrally and only a few millimetres medially of the facies articularis humeralis. The sulcus m. supracoracoidei is thus broad and shallow and faces medially (Figs 6N, 6O and 7N, 7O). The cotyla scapularis varies from slightly concave to slightly convex, has angular margins, and is roughly as long as it is wide (Figs 6N, 6O and 7N, 7O). The cotyla has its sternal margin raised above the shaft so is aligned obliquely to the shaft. It has a rough texture indicating a strong ligamentous connection to the scapula, as noted by Poplin and Mourer-Chauviré [12]. The facies articularis humeralis extends ventrally from the cotyla and is only slightly offset laterally, less so than in volant galliforms (Figs 6N and 7N). It has comparable width, but is dorsoventrally longer than the cotyla scapularis. Its lateral part is slightly concave, but as it extends onto the cranial facies ventral to the cotyla, the facies articularis humeralis is convex in the lateromedial section. There is a pneumatic or vascular foramen laterally immediately sternally of the junction of the facies articularis humeralis and the cotyla scapularis. The marked reduction in the acrocoracoid has resulted in the facies articularis clavicularis becoming a rugose zone on the medioventral facies of the acrocoracoid that is aligned roughly parallel to the shaft (Figs 6O and 7O), rather than in a dorsoventral plane at about right angles to the shaft as in megapodes. The overall form of the omal end of the coracoid in dorsal view is that the cotyla scapularis, facies articularis humeralis and acrocoracoid form a shallow C-shape (Figs 6N and 7N), rather than the marked, near right angle, that characterises galliforms. There is no processus procoracoideus and no foramen nervi supracoracoidei present in any specimen, contra the observations for one specimen reported by Poplin and Mourer-Chauviré [12]. A distinct intermuscular ridge connects the impressio m. sternocoracoidei to the medial side of the cotyla scapularis. This is traversed in a medial-omal—laterosternal direction by a thin vascular groove creating a notch in the ridge about 15 mm below the cotyla, and the crest itself has occasional minute vascular foramina in it, but no feature interpretable as a foramen nervi supracoracoidei was observed. The foramen described by Poplin and Mourer-Chauviré [12] is very small and separated from the inner edge by a very thin bone crest, which is consistent with our observations and may be explained as the intermuscular crest being slightly more developed and having enveloped the vessel.

**Table 4:** Summary statistics of measurements (mm) for coracoids of *Sylviornis neocaledoniae*. Abbreviations: L, length; AL, acrocoracoid length from the cotyla scapularis; minSW, minimum shaft width in dorsal view; StnW, sternal width.

	TL	AL	minSW	StnW
Mean	107.6	23.8	6.4	16.3
Standard Deviation	3.68	0.48	0.20	0.92
Minimum	105.0	23.0	6.2	15.6
Maximum	110.2	24.2	6.7	16.9
Count	2	8	7	2

The sternal end is narrow in dorsal view with a shallow impressio m. sternocoracoidei that is restricted to the sternal third of length (Figs 6N and 7N). The sternal end is, however, surprisingly thick, with a depth about 66% of width, but is not robust as the surface bone is very thin. At the omal end of the impressio m. sternocoracoidei, a prominent ligament attachment site results in the shaft being elevated dorsally giving it an angular appearance in lateral view. The impressio contains pneumatic foramina sternally and in an area along its lateral margin, but is less pneumatic than in megapodes such as *Leipoa* and *Alectura*. The ventral shaft surface is compressed into a ridge from the base of the acromion to about mid-length. More sternally the ventral surface is rounded so that the facies articularis sternalis has an evenly laterally-convex profile in sternal aspect. The articular surfaces of the ventral and dorsal facets (facies interna and facies externa) are continuous. The ventral facet is flat and deep and separated by a shallow central ridge (crista intermedia) from the dorsal facet. A processus lateralis is variably present, e.g. it is present but is very reduced on IANCP1099, but is entirely absent on IANCP1032.

#### Sternum (Figs 6R and 7R)

Material: IANCP1060, a fragment of the right side of a sternum preserving the coracoid articular facet and the costal articulation area. There are four processus costales each with an incisura intercostalis caudal to it. The pars cardiaca is pneumatic especially dorsal to the coracoid buttress. Ventrally, the facies articularis coracoideus is a prominent buttress about 17 mm wide and 10 mm thick dorsoventrally (Figs 6R and 7R). The middle third joins to a rounded, cranially-directed, ridge about 6 mm wide which articulates with the dorsal part of facies articularis sternalis on the coracoid. When articulated, the processus lateralis of the coracoid extends caudally past the buttress on its lateral side, but the lateral side of the coracoid is about 15–18 mm mesad of the lateral side of the sternum.

#### Femur (Fig 8A–8E)

**Fig 8:**
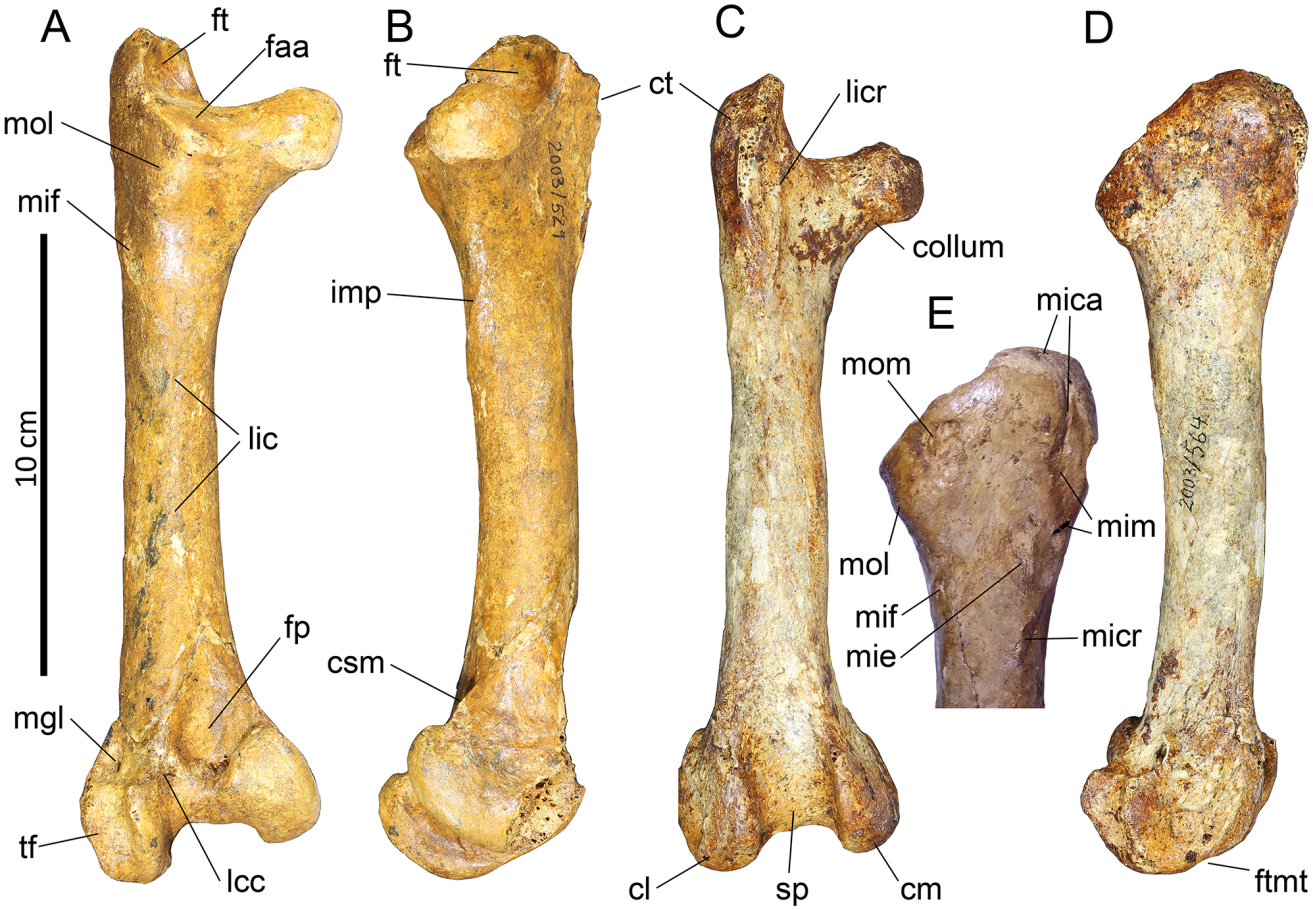
Femora of *Sylviornis neocaledoniae*. (A, B) left, IANCP529; (C, D) right, IANCP564; and (E) proximal right, IANCP527+535, in caudal (A), medial (B), cranial (C) and lateral (D, E) views. Abbreviations: c, collum; cl, condylus lateralis; cm, condylus medialis; csm, crista supracondylaris medialis; ct, crista trochanteris; faa, facies artic. antitrochanterica; fp, fossa poplitea; ft, fossa trochanteris; ftmt, fovea tendineus m. tibialis; imp, insertion for m. puboischiofemoralis; lcc, insertion for lig. cruciatum craniale; lic, linea intermuscularis caudalis; licr, linea intermuscularis cranialis; mica, insertion for m. iliotrochanterica caudalis; micr, insertion for m. iliotrochanterica cranialis; mie, insertion for m. iliofemoralis externus; mif, insertion for ischiofemoralis; mim, insertion for m. iliotrochanterica medialis; mgl, insertion for m. gastrocnemialis lateralis; mom, insertion for m. obturatorius medialis; mol, insertion for m. obturatorius lateralis; sp, sulcus patellaris; tf, trochlea fibularis.

Material: IANCP526+543, s+pL fem; IANCP527+535, pR fem; IANCP528, R fem; IANCP529, L fem; IANCP530, juv R fem; IANCP531+547, L fem; IANCP532, R fem (-d); IANCP533, R fem (dorsal half); IANCP534, R fem; IANCP538, dR fem; IANCP539, frag pR fem; IANCP540, dL fem; IANCP542, pR fem; IANCP544, pt dL fem; IANCP545, pL fem; IANCP546, pt dR fem; IANCP548, L fem; IANCP564, R fem; IANCP568, sL fem; IANCP1025, pR fem; IANCP1026, pR fem; IANCP1029, juv R fem; IANCP1030, shaft fem; IANCP1031, pL fem.

Poplin and Mourer-Chauviré [12] had no complete examples of this bone and their description was brief. The femora of *Sylviornis neocaledoniae* have broadly similar proportions to those of megapodes (Table 5). In lateral aspect, the shaft is slightly bowed dorsally and the crista trochanteris projects proximodorsally of the collum and caput (Fig 8D). The crista trochanteris encloses a well-developed fossa trochanteris which sometimes has pneumatic foramina in its base. The facies articularis antitrochanterica is convex in the craniocaudal plane and projects caudally as a lip overhanging the caudal facies (Fig 8A and 8B). On the cranial facies, the crista trochanteris is short and extends distally of the collum a distance equivalent to the proximodistal width of the caput (Fig 8C). The facies mesad of the crista trochanteris is flat, not concave. The linea intermuscularis cranialis extends along the shaft to merge with the distal end of the crista trochanteris then passes mesad of the crista towards the facies articularis antitrochanterica, so enclosing an oval area about 30 mm long by 10 mm wide that bears pneumatic foramina (Fig 8C). The caput is small and lacks a fovea ligamentum capitis. The collum is not constricted in caudal aspect, although it is slightly so in proximal view. A large (15 by 12 mm) rounded prominence on the caudolateral area adjacent to the facies articularis antitrochanterica is interpreted as the impressiones obturatoriae principally for the insertion of m. obturatorius lateralis. Lying close to the proximal end and just caudally of mid-depth is a large (c. 15 mm long and c. 12 mm broad) recessed scar for the insertion of the m. obturatorius medialis which is the obturatorius muscle that leaves the most marked scar [73–75].

**Table 5:** Measurements (mm) of femora of *Sylviornis neocaledoniae*: Proximal width (PW) is maximum, shaft width and depth (SW, SD) were taken at mid length, and distal width (DW) is maximum. Contributing specimens: IANCP526+543, IANCP527+535, IANCP528, IANCP529, IANCP531+547, IANCP532, IANCP534, IANCP538, IANCP542, IANCP564, IANCP1031.

	TL	PW max	SW	SD	DW
Mean	193.0	56.2	21.6	22.9	53.0
Standard Error	3.25	0.37	0.39	0.81	0.52
Standard Deviation	7.97	0.99	1.02	2.14	1.15
Minimum	179.5	54.1	20.5	19.1	51.4
Maximum	200.8	57.0	23.0	26.0	54.6
Count	6	7	7	7	5

The remaining muscular insertions on the proximal lateral facies are best seen in IANCP527+535 (Fig 8E) and conform to the general galliform plan illustrated by Zinoviev ([74]: Fig 22). The largest is an elongate slightly sigmoid sulcus lying about 10 mm from the crista trochanteris, and extending from the proximal margin to the distal end of the crista trochanteris, for the insertion of the mm. iliotrochanterici caudalis et medius (the caudalis is most proximal). A continuation of this scar for the insertion of m. iliotrochantericus cranialis, separated from the former by about 10 mm, extends distocaudally from the end of the crista trochanteris to a point about 70 mm from the proximal end. The m. iliotrochantericus cranialis is thus located distal to the distal end of the crista trochanteris. For these three scars, the insertion facies indicate that the fibres were directed dorsally towards the trochantal crest. The insertion area of the m. iliofemoralis externus roughly straddles the space between the insertions for m. iliotrochantericus medius and m. iliotrochantericus cranialis, but is offset caudally about 10 mm. Its proximocaudal margin is some 15 mm distocranial of the insertion of the m. obturatorius medialis. Its impression shows the fibres were directed proximally and can nearly form a pocket (e.g. IANCP531+547). The insertion area of the m. ischiofemoralis is an elongate (15 mm long) scar located about 10 mm further caudad of the m. iliofemoralis externus and straddles the laterocaudal margin of the shaft. It extends from about the level of the distal end of the crista trochanteris distocaudally towards the caudolateral margin and has a scar that indicates attached fibres were directed proximocaudally. The insertions of m. ischiofemoralis and m. iliofemoralis externus are thus much more widely separated than in *Gallus gallus* and megapodes, wherein they are close together and nearly centred on the lateral facies.

The linea intermuscularis caudalis has one crest centrally on the caudal facies at mid length, but slightly further proximally it splits in two at a point sometimes marked by a nutrient foramen. The crest, directed proximomedially, is robust and elevated and presumed to be the insertion of m. caudofemoralis and merges with another elevated crest on the medial margin that is presumed to be for the insertion of m. puboischiofemoralis pars medialis. The lesser crest is directed proximomedially and passes just distad of the scar for the insertion of m. ischiofemoralis.

The insertion of m. gastrocnemialis lateralis [the term tuberculum is inappropriate given the recessed nature of the insertion] is a near circular scar about 10 mm wide that is deeply recessed into the caudal facies proximally adjacent to the trochlea fibularis and facing caudally (Fig 8A and 8D). The caudal impressio ansae m. iliofibularis forms a shallow rugose scar on the lateral facies aligned craniocaudally and located slightly distad of the insertion of m. gastrocnemialis lateralis (e.g. IANCP564). The cranial impressio ansae m. iliofibularis is marked by striations caudocranially traversing the craniolateral margin about 20 mm from the insertion of m. gastrocnemialis lateralis. The fossa poplitea is restricted to the medial half of the bone and forms a deep, steep-sided, pneumatic pocket into the base of the medial side of condylus medialis. The fossa poplitea is bound medially by a sharp crista supracondylaris medialis that continues as a linea intermuscularis caudalis proximal to the fossa (Fig 8A). There are no transversely aligned prominent ligamental insertion scars, such as seen in megapodes or *Gallus*, present within the fossa. However, between the edge of the fossa and the condylus lateralis, there is a distinct, wide (10 mm), triangular and shallow sulcus for the insertion of the ligamentum cruciatum craniale. Caudally, there is a broad U-shaped groove, deepest laterally where it leads to the last mentioned sulcus, separating the condylus medialis from the condylus lateralis. Its width is such that the lateral edge of the condylus medialis does not reach half way across distal width, in contrast to *Gallus* where the condylus medialis extends much further laterally. The condylus lateralis projects slightly more caudally than the condylus medialis but both have equal cranial projection. The sulcus patellaris is broad and deep and forms a deep notch in the distal profile (Fig 8C). The impressio ligamentum cruciati cranialis is strongly marked forming an impression distally in the sulcus intercondylaris that is partly excavated into the base of the condylus lateralis and extends mediocaudally towards the base of the condylus medialis. There is no epicondylus medialis and the medial facies of the condylus medialis is flat. Laterally, there is a deep, ovoid, and centrally placed impressio ligamentum collateralis lateralis. The caudally directed trochlea fibularis, in caudal aspect, is widest proximally, narrows distally, and extends to the distal end of the condylus lateralis, as in other galliforms. Distally, the lateral condyle bears a shallow fovea tendineus m. tibialis cranialis.

Five specimens (IANCP527+535, IANCP529, IANCP531+547, IANCP532, IANCP564) preserved the shaft allowing mid-shaft circumference measurements. These ranged 74–81 mm and from these estimated mass for *S*. *neocaledoniae* was calculated as follows: mean 34.4 kg, range 27.6–34.4 kg, standard deviation 2.51, using the algorithm in Campbell and Marcus [36], or 34.1 kg, range 27.4–34.1 kg, standard deviation 2.48, using the algorithm in Field et al. [37].

#### Tibiotarsus (Fig 9A–9E, Table 6)

**Fig 9:**
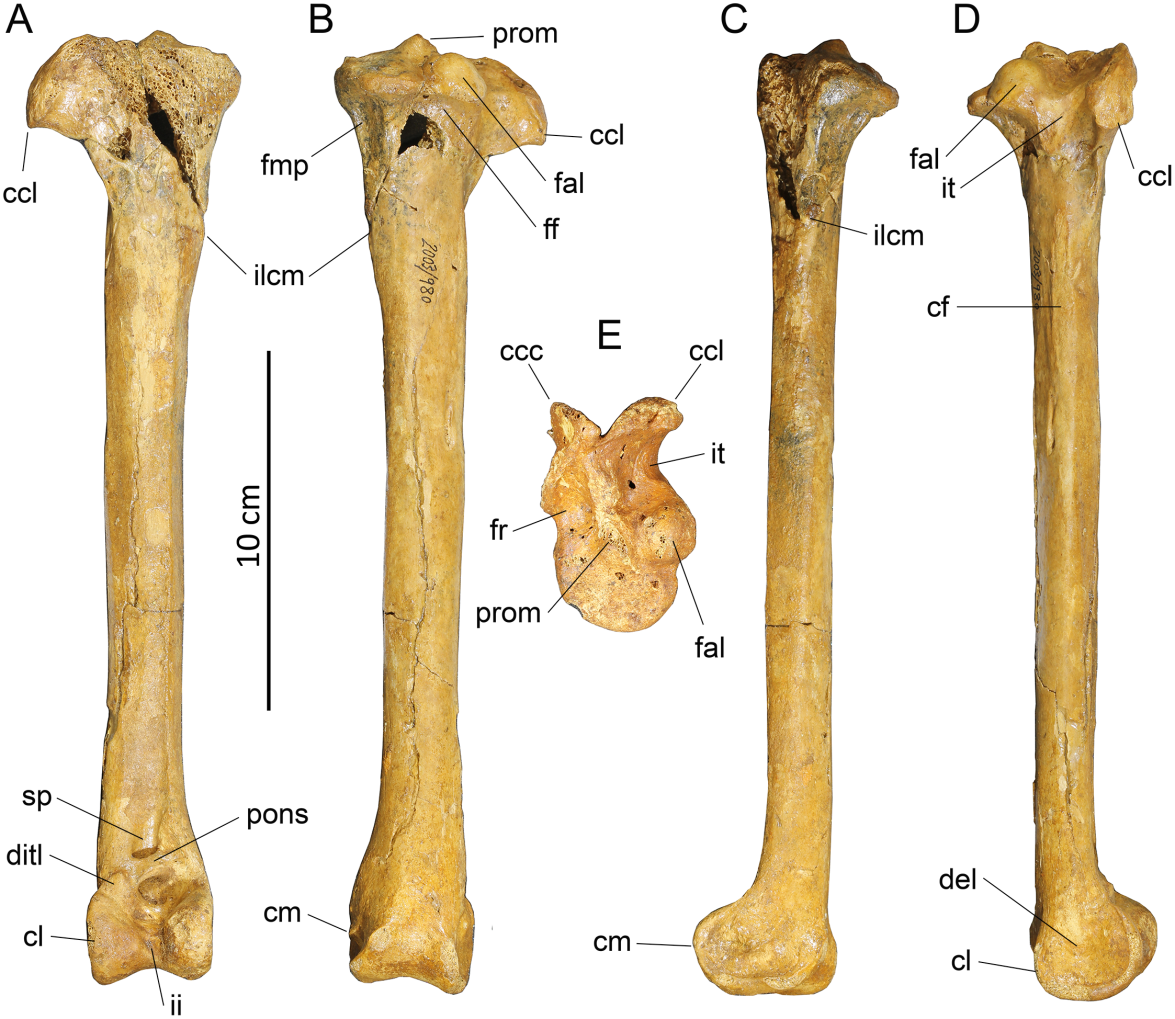
Tibiotarsi of *Sylviornis neocaledoniae*. (A-D) right, IANCP980 and (E) proximal right IANCP1013, in anterior (A), caudal (B), medial (C), lateral (D) and proximal (E) views. Abbreviations: ccc, crista cnemialis cranialis; ccl, crista cnemialis lateralis; cf, crista fibularis; cl, condylus lateralis; cm, condylus medialis; del, depressio epicondylaris lateralis; ditl, distal insertion of transverse ligament; fal, facies artic. lateralis; ff, fossa flexoria; fmp, fossa for origin of m. plantaris; fr, fossa retropatellaris; iclm, impressio lig. collateralis medialis; ii, incisura intercondylaris; it, incisura tibialis; pons, pons supratendineus; prom, prominence in area interarticularis; se, sulcus extensorius.

Material: IANCP 717, L proximal tibial epiphysis; IANCP718, juv L tib; IANCP723, dR tib; IANCP980, R tib; IANCP981, dL tib; IANCP982, R tib; IANCP983, dL tib; IANCP984, dL tib; IANCP985, dR tib; IANCP986, dL tib; IANCP987, juv tib; IANCP988, pL tib; IANCP989, dR tib; IANCP990, dL tib; IANCP991, dR tib; IANCP992, dR tib; IANCP993, dL tib; IANCP994, dR tib; IANCP995, dL tib; IANCP1003, pR tib; IANCP1004, pL tib; IANCP1005, pR tib; IANCP1006, pR tib; IANCP1007, pL tib; IANCP1008, dR tib; IANCP1009, pR tib; IANCP1010, pL tib; IANCP1011, pR tib; IANCP1012, pR tib; IANCP1013, pR tib; IANCP1027, dR tib; IANCP1028, pL tib.

As for femora, the tibiotarsi from Ile de Pins are fragmentary and poorly preserved [12]. Among our material, only one specimen, IANCP980, preserves near total length and it is missing the proximal part of the crista cnemialis cranialis (Fig 9A–9D). It is 265 mm long, and from the relative extension of the cnemial crest in IANCP1003, another 3–4 mm at most can be added to this length for a total of 269 mm. The bone is robust with a mid-shaft width about 8% of length (Table 6). As shown by IANCP1003 and 1013, the crista cnemialis cranialis projects only slightly proximad of the area interarticularis. It also has very limited distal extent with its distal end about level with the hook of the crista cnemialis lateralis, and its cranial projection, best seen in IANCP1004, is limited at about 12 mm. On the proximal surface, the fossa retropatellaris is a distinct fossa open to the medial facies that is separated by a robust ridge from the incisura tibialis (Fig 9E). This ridge links the crista cnemialis cranialis with an ovoid prominence centrally in the area interarticularis. The facies articularis lateralis is globose and very prominent (rising c. 10 mm), and is separated from both the facies articularis medialis caudally and the central elevation of area interarticularis by distinct grooves. It is much more defined than in megapodes such as *L*. *ocellata* and *A*. *lathami*. The incisura tibialis is about 20 mm long craniocaudally, 15 mm wide, and recessed about 10 mm below the last-mentioned ridge so its base is about level with the lateral part of facies articularis medialis. This is therefore shallower than in galliforms such as *G*. *gallus* and *A*. *lathami*. In proximal view, this incisura forms a notch about 10 mm deep between the crista cnemialis lateralis and the edge of facies articularis lateralis. The crista cnemialis lateralis has a very robust, broad and flattened proximolateral surface for the insertions of m. femorotibialis externus and m. femorotibialis medius (sensu Zinoviev [74]), a tip that is only slightly hooked distally, and a ventral margin forming a crest that extends down the shaft to a point level with the proximal end of the crista fibularis (Fig 9A, 9B and 9D). On the caudal margin of the proximal end of the crista cnemialis lateralis is a distinct foramen (e.g., in IANCP980, 1003, 1006, 1013), in which location there is a shallow, but more elongate groove in *A*. *lathami*, *M*. *reinwardt*, *L*. *ocellata* and *G*. *gallus*. The fossa flexoria for the origin of m. flexor digitorum longus is broad (c. 20 mm) and undercuts both the facies articularis lateralis and half of the facies articularis medialis and lacks pneumatic foramina. Adjacent to it medially, and separated by a narrow crest is a narrow (c. 10 mm) fossa on the caudomedial facies for the origin of m. plantaris, similar to that typically found in megapodes. The impressio ligamentum collateralis medialis is barely protuberant from the surrounding facies, and is thus much less protuberant than that of megapodes, e.g. *A*. *lathami*, but as in that species, its length overlaps with the proximal end of the crista fibularis.

**Table 6:** Measurements (mm) of tibiotarsi of *Sylviornis neocaledoniae*. Abbreviations: TL, total length is the maximum from the crista cnemialis cranialis to distal end of the condylus medialis, which was estimated here based on IANCP 980 completed with reference to IANCP1013; PW, proximal width is the maximum across the articular facies; PD proximal depth is from crista cnemialis cranialis to the caudal margin of facies articularis medialis; SW, shaft width is the minimum in the distal third of shaft; DW, distal width is a maximum; DLC, depth condylus lateralis was taken on a plane at right angle to shaft; DMC, depth condylus medialis was maximum craniocaudal depth. Contributing specimens: IANCP718, IANCP723, IANCP980, IANCP981, IANCP982, IANCP983, IANCP984, IANCP985, IANCP986, IANCP988, IANCP989, IANCP990, IANCP991, IANCP992, IANCP993, IANCP994, IANCP995, IANCP1003, IANCP1004, IANCP1005, IANCP1007, IANCP1008, IANCP1009, IANCP1010, IANCP1012, IANCP1013, IANCP1027.

	TL	PW	PD	SW	DW	DLC	DMC
Mean	269.0	42.1	64.5	19.6	34.7	29.8	36.3
Standard Deviation		1.15	1.71	0.61	1.60	0.88	1.16
Minimum		40.4	62.8	18.8	32.1	28.5	33.8
Maximum		44.2	66	20.4	37.4	31.1	37.8
Count	1	8	4	7	15	13	16

The shaft is straight with parallel sides and bears a marked linea intermuscularis cranialis that extends from the end of the crista cnemialis cranialis down the medial margin to a point near the pons supratendineus. The crista fibularis is not prominent on the lateral margin, unlike megapodes, and at c. 45 mm long, is about 17% of tibiotarsal length. The nutrient foramen is large and located distal of the crista fibularis on the caudal surface close to the lateral margin. The cranial facies is convex over most of this length with a shallow sulcus extensorius extending about 70 mm proximal to the pons. The distal insertion of the ligamentum transversum is on a proximolateral-distomedial aligned prominence laterad of the pons supratendineus. The ligament’s proximal insertion is a scar flush with the surface at the proximomedial side of the pons adjacent to the sulcus extensorius, as is typical of galliforms.

The pons supratendineus has a proximomedial to distolateral alignment as usual for galliforms (Fig 9A). It is narrower proximodistally than it is wide, so contrasting with most examined galliforms, wherein the pons is longer than wide. The distal end of the sulcus extensorius is secondarily excavated with foramina in its base at a point just distal to the pons. The incisura intercondylaris is narrow, as usual in megapodes, and more so than that seen in *G*. *gallus*. However, because of a cranially-low condylus lateralis, the incisura is open laterally in the cranial half of its depth, and thus not as steeply parallel-sided in distal view as in megapodes. In distal view the condylus medialis has greater cranial projection than the condylus lateralis, but both are relatively short in cranial view so that proximodistal length (c. 27 mm) is greatly exceeded by the distal width (c. 35 mm), versus only slightly so in *Gallus gallus*.

The sulcus m. fibularis [peronei] faces laterally, is shallow and defined by very low crests for the insertions of the retinaculum m. fibularis. In lateral view, the condylus lateralis has a round profile that is slightly flattened distally, and which does not project caudal to the shaft. The depressio epicondylaris lateralis is shallow and deepest centrally (Fig 9D). In medial aspect, the condylus medialis is craniocaudally elongate and distinctly flattened distally. The epicondylaris medialis small and obscured in cranial view, and is bordered anteriorly and distally by a shallow depressio epicondylaris medialis between it and the condylar margin. In the juvenile specimen IANCP718, the partially fused distal tarsus shows that the astragalar process extended 20 mm proximal of the condylus lateralis.

#### Tarsometatarsus (Fig 10A–10F)

**Fig 10:**
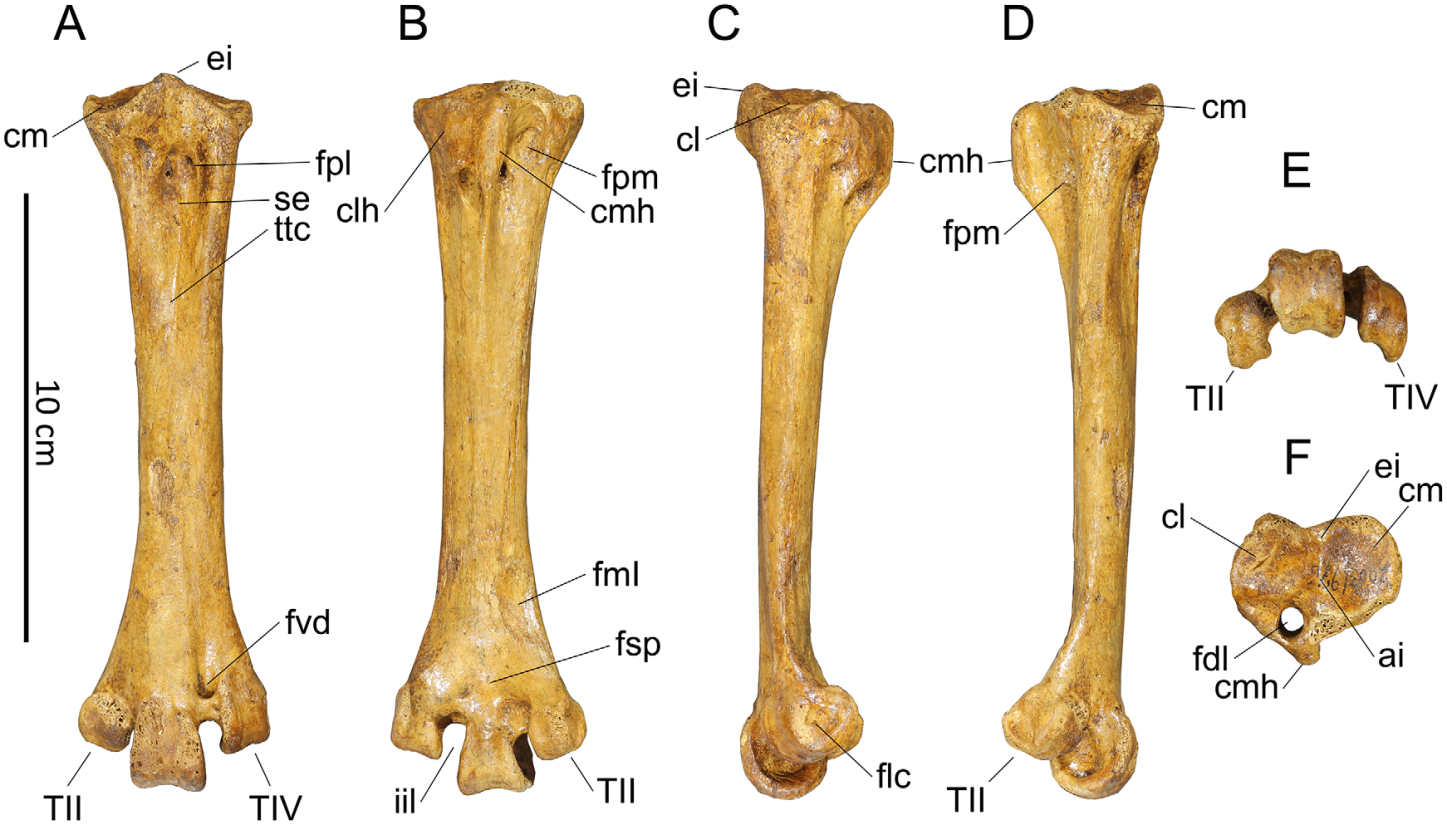
Left tarsometatarsus of *Sylviornis neocaledoniae*. (A-F) IANCP976, in anterior (A), caudal (B), lateral (C), medial (D), distal (E) and proximal (F) views. Abbreviations: ai, area intercotylaris; cl, cotyla lateralis; clh, crista lateralis hypotarsi; cm, cotyla medialis; cmh, crista medialis hypotarsi; ei, eminentia intercotylaris; fdl, sulcus for tendon of m. flexor digitorum longus; flc, fovea ligamentum collateralis; fmI, fossa metatarsi I; fpl, foramen vasculare proximale lateralis; fpm, fossa parahypotarsalis medialis; fsp, fossa supratrochlearis plantaris; fvd, foramen vasculare distale; iil, incisura intertrochlearis lateralis; se, sulcus extensorius; TII, trochlea metatarsi II; TIV, trochlea metatarsi IV; ttc, tuberositas m. tibialis cranialis.

Material: IANCP536, p+sL tmt; IANCP537, pR tmt; IANCP541, 2 parts R tmt; IANCP549, juv pR tmt; IANCP550, juv R tmt; IANCP551, trochlea III of L tmt; IANCP552, juv dR tmt; IANCP553, pt juv pR tmt; IANCP554, 2 frags pR tmt; IANCP719, juv L tmt, tarsal not synostosed but perfect; IANCP722, juv R tmt; IANCP971, L tmt; IANCP972, L tmt; IANCP973, R tmt; IANCP974, L tmt; IANCP975, L tmt; IANCP976, L tmt; IANCP977, dR tmt; IANCP978, pt R tmt; IANCP979, L tmt.

Poplin and Mourer-Chauviré [12] provided a fairly comprehensive description of the tarsometatarsus, although some aspects were compromised by breakage such as the proximal end. A complete description is given here and the data used in our phylogenetic and functional inferences. Several complete to near-complete tarsometatarsi are available with a mean length of c. 158 mm (Table 7, Fig 10). They are larger than those of all megapodiids, and robust, with proportions similar to species of *Leipoa*, *Megapodius* and *Alectura*, whereas those of *Aepypodius* and *Macrocephalon* are more elongate, as noted by Poplin and Mourer-Chauviré [12]. The cotyla medialis is proximodistally level with the cotyla lateralis, but projects more cranially and is craniocaudally deeper (Fig 10A and 10F). The eminentia intercotylaris in cranial aspect is centred on the shaft, triangular, and projects proximally of the cranial cotylar margin, but in lateral aspect does not project proximal to the area intercotylaris nor the caudal margin of the cotyla medialis (Fig 10A and 10C). Cranially, the sulcus extensorius has a width about one third of the shaft and extends to only about 25% of total length (Fig 10A). It begins at the foramina vascularia proximalia and there is no fossa infracotylaris dorsalis proximal of the foramina. The foramen vasculare proximale medialis is 2–3 times larger than, and displaced distally from, its lateral equivalent. The impressiones retinaculi extensorii are low barely perceptible crests medial to the proximal limit of the sulcus extensorius. The tuberositas m. tibialis cranialis comprise a pair of impressions (Fig 10A), the larger one lies distal to the foramen vasculare proximale medialis on the medial wall of the sulcus extensorius, while the smaller one is centred on the length of its medial counterpart on the lateral wall of the sulcus extensorius. Both scars have little elevation above the adjacent facies and so are wholly confined in the sulcus extensorius in lateral or medial aspect.

**Table 7:** Measurements (mm) for tarsometatarsi: TL (greatest length), PW proximal width from cotyla medialis to cotyla lateralis, PD proximal depth from crista medialis hypotarsi to the highest point of the cotyla medialis, DW distal width across trochlea metatarsi II to trochlea metatarsi IV, DMC depth of medial cotyla, WFPM width of fossa parahypotarsalis medialis, SW1 width of shaft at thinnest point = midlength, SW2 width of shaft at proximal side of fossa metatarsi I, SD1 shaft depth at SW1, SD2 shaft depth at SW 2, HD, hypotarsus depth from the base of the hypotarsus to the eminentia intercotylaris, DT3 depth of lateral rim of trochlea metatarsi III, WT3 width of trochlea metatarsi III.

	TL	PW	PD	DW	DMC	WFPM	SW1	SW2	SD1	SD2	DT3	WT3
Mean	157.8	37.3	36.5	42.2	24.5	15.5	17.4	19.5	11.8	10.5	20.1	16.6
Standard Deviation	4.53	1.19	0.76	0.63	0.79	1.26	0.81	1.41	0.87	0.53	1.15	0.38
Minimum	151.2	34.8	35.7	41.4	23.5	14.2	16	17.3	10.8	9.6	18.5	16
Maximum	163.4	39.5	37.5	42.8	25.8	18	18.5	21.3	12.9	11.2	22.2	17.2
Count	8	11	6	4	9	7	8	10	8	10	9	8

The hypotarsus is located entirely laterad of the eminentia intercotylaris (Fig 10B and 10F). The crista medialis hypotarsi is the deepest crista, but its depth is much less than that of the adjacent cotyla lateralis. The hypotarsus is triangular in plantar view, shorter than wide, longest medially and draws to a point proximolaterally. Best observed in IANCP973 and 976, its plantar surface is nearly smooth, with only a slight elevation marking the cristae intermediae hypotarsi, which is separated from the crista medialis by a very shallow sulcus on the surface plantar of the canal. Its flattened surface is, however, directed considerably laterally, not plantarly as indicated by Poplin and Mourer-Chauviré [12: Pl. 4.1]. The crista lateralis hypotarsi is taken to be a relatively small triangular section of the hypotarsus located immediately laterad of the cristae intermediae hypotarsi and proximal to the plantar exit of the lateral vascular foramen (Fig 10B). There is no sulcus for m. fibularis longus laterad of the hypotarsus. The hypotarsus is monocanaliculate (sensu [76]), with a single canal for the tendon of m. flexor digitorum longus, however, as the canal is deeper than wide, it probably carried other tendons, either the tendon of m. flexor perforatus digiti 2 (fp2) or the tendon of m. flexor perforans et perforatus digiti 2 (fpp2), which lie plantar to fp2. In medial aspect, the profile of the crista medialis hypotarsi gradually lowers to the shaft distally, i.e. does not end abruptly, and it lacks a hook.

The fossa parahypotarsalis medialis is fairly broad, occupying about half of corpus width, but is short, such that it does not extend to midlength, and shallow being bound by a thick shaft dorsally (Fig 10B and 10D). The fossa parahypotarsalis medialis is thus relatively much smaller than in megapodes like species of *Leipoa*, *Megapodius*, *Alectura*, *Aepypodius* and *Talegalla*, which all have the shaft dorsal to the fossa very dorsoplantarly compressed. The fossa parahypotarsalis lateralis is very narrow and shallow.

The shaft is wider than deep and convex cranially over its distal half. Minimum shaft width is at mid length and widths increase both proximally and distally of that point, that is there is no mid-section with parallel sides. The fossa metatarsi I is shallow, lacks a distinct facet, does not impact on the medial margin of the shaft, and its width at fossa mid-length, is about 35% of the adjacent shaft width.

Distally the trochlea diverge symmetrically from the shaft, trochleae metatarsi II and IV have equal distal projection, and trochlea metatarsi III, while longest, is short, extending less distally of the incisura intertrochlearis lateralis than its width (Fig 10A). Trochlea metatarsi IV is slightly broader than trochlea metatarsi II and both are much narrower than trochlea metatarsi III. The greater width of trochlea metatarsi III is seen in *Progura* spp., but in the smaller species of *Leipoa*, *Alectura* and *Megapodius* the trochleae are all roughly the same width. All trochleae are grooved dorsally and distally, although the grooves are shallow on trochleae metatarsi II and IV. On trochlea metatarsi III, dorsally the medial rim extends further proximally than the lateral one, but plantarly, asymmetry results in greater proximal extent of the lateral rim. Trochlea metatarsi IV has a weakly developed flange extending plantarly from its lateral rim (Fig 10E), but that on trochlea metatarsi II is even smaller. Both trochleae metatarsi II and IV have deep foveae ligamenta collateralium. The foramen vasculare distale, as noted by Poplin and Mourer-Chauviré [12], is large dorsally and extends via a broad canalis interosseous distalis, for vessels and the tendon of m. extensor brevis digiti IV, to the incisura intertrochlearis lateralis: only a minute foramen marks the plantar exit of the foramen vasculare distale (Fig 10A and 10B). The fossa supratrochlearis plantaris is shallow, but at its mediodistal margin, it is secondarily deepened adjacent to the incisura intertrochlearis medialis into a distinct oval fossa which undercuts the rim of trochlea metatarsi III.

A near full-sized juvenile specimen (IANCP719) has an unfused proximal tarsal and incompletely synostosed metatarsals. Metatarsal II is the broadest of the three metatarsals proximally and is separated from the other two by an elongate (18 by 3 mm) foramen. Metatarsal III, in proximal view, inserts as a triangular wedge between metatarsals IV and II and is grooved plantarly by a sulcus that precedes formation of the fdl canal. The lateral vascular foramen is tiny. The proximal tarsal, in proximal view, has all the features of the adult, but plantarly the hypotarsus had not ossified to the extent of enclosing the fdl canal, although a depth and width of crista medialis hypotarsi similar to that seen in adult bones, had been attained. Distally the canalis interosseous distalis had not formed.

#### Pedal phalanges and os metatarsal I (Fig 11)

**Fig 11:**
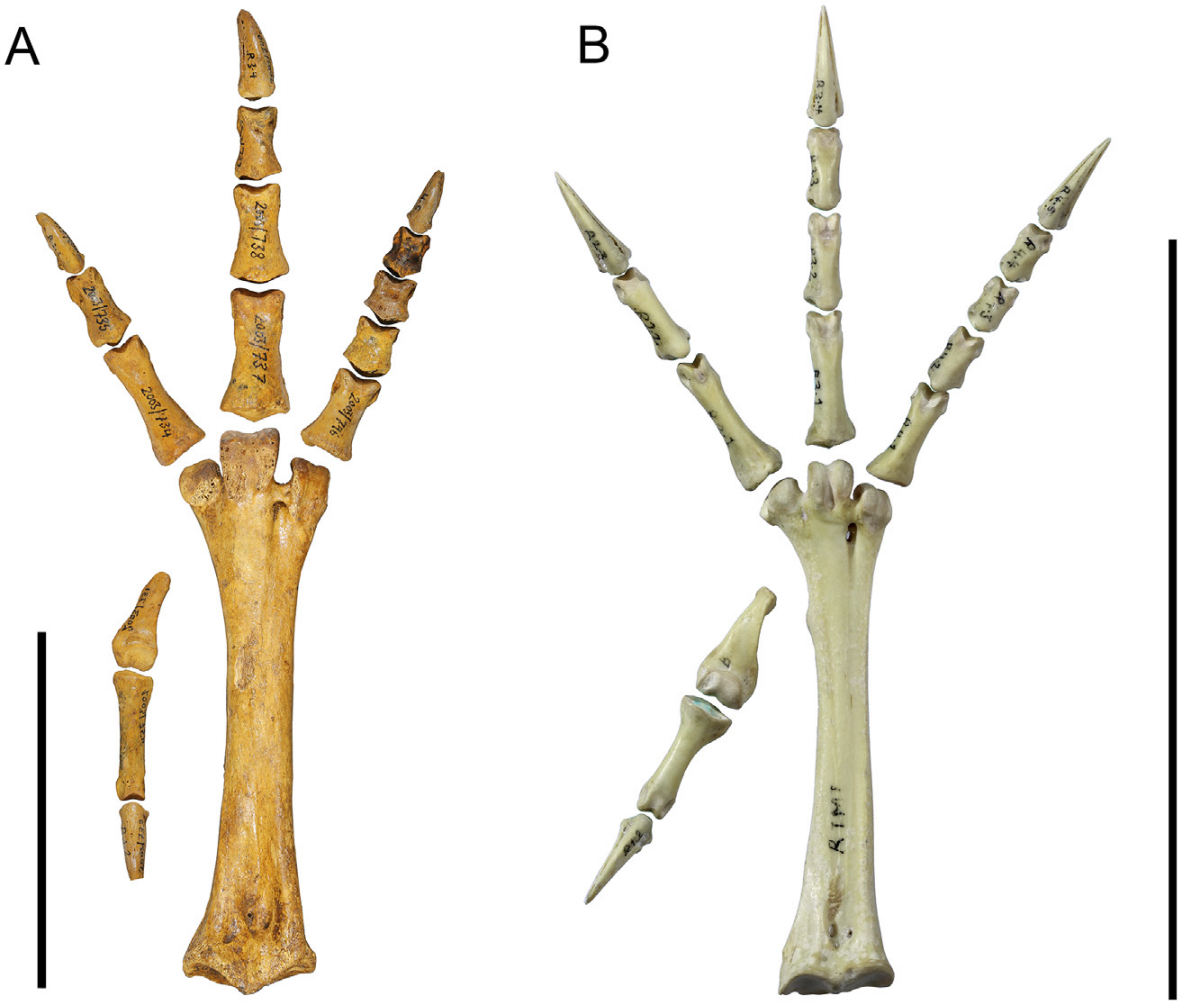
Composite foot of *Sylviornis neocaledoniae* compared to that of *Leipoa ocellata* scaled to the same absolute size. (A) *Sylviornis neocaledoniae*: tarsometatarsus is IANCP976 reversed, the phalanges are IANCP731 (reversed), 733–745, and 754 (reversed). (B) *Leipoa ocellata* SAM B.11482. Note that the digits of *S*. *neocaledoniae* are relatively shorter, especially digits 2 and 4, and the unguals are much shorter. Scale bars are 10 cm.

*Material*: All phalanges identified to element are listed here, but only those considered to be adult are used in the measurements for which summary statistics are provided belowand the mean used to compare to other taxa (S1 File).

Numerous phalanges are known for *Sylviornis neocaledoniae*. While no articulated material is known, the collection reveals that this bird had the usual digital formula of 2:3:4:5 for digits I to IV as shown in a composite set (Fig 11) assembled based on matching size of the elements from The Pocket, in Cave B. The phalanges are all relatively short and robust compared to all extant megapodes (Tables 8–22, [12]). The unguals are also relatively shorter than in all megapodes, compared to proxies for body size, e.g. femur length width (S1 File). The unguals are compressed lateromedially, especially at midlength in the area covered by the horny sheath, with mean Width:Depth ratios as follows I.2 (5.9:8.2); II.3 (7.5:7.9); III.4 (8.3:8.1); IV.5 (7.0:7.8), thus only III.4 is on average slightly wider than deep. None are notably flattened ventrally and all lack a markedly angular transition from the sides to the ventral surface. Ungual I.2 is more curved than the other unguals and has a prominent tuberculum extensorium. All other unguals effectively lack a tuberculum extensorium. The metatarsal 1 is relatively elongate and similar in shape to that of megapodes, as noted by Poplin and Mourer-Chauviré [12].

**Table 8:** Measurements (mm) of metatarsal I. The proximal facet articulates with the metatarsal facet on the tarsometatarsus. Material: IANCP731, IANCP764-767, IANCP833-836, NISP = 9.

	TL	Length of proximal facet	Width distal condyle	Depth of distal condyle
Mean	27.9	11.4	9.9	7.6
Standard Deviation	1.16	1.22	0.80	0.72
Minimum	25.9	9.7	8.7	6.6
Maximum	29.3	13.4	11.5	8.7
Count	6	7	8	9

**Table 9:** Measurements (mm) of phalanx I.1. Material: IANCP572, IANCP732, IANCP749-756, IANCP815, IANCP839-852. NISP = 25.

	TL	PW	PD	SW	DW	DD
Mean	34.4	11.3	8.7	6.3	8.3	8.1
Standard Deviation	1.56	0.78	0.81	0.61	0.74	0.50
Minimum	30.3	9.7	7.6	5.3	7.0	7.3
Maximum	36.6	12.8	10.2	7.7	9.2	9.1
Count	16	17	14	19	19	17

**Table 10:** Measurements (mm) of phalanx I.2. Material: IANCP733, IANCP801-802, IANCP856, IANCP863, IANCP874-875, IANCP876-883; NISP = 15.

	TL	PW	PD	SW	SD
Mean	20.9	7.7	10.1	5.9	8.2
Standard Deviation	1.83	0.67	0.83	0.31	0.64
Minimum	18.8	6.6	9.0	5.5	7.2
Maximum	23.3	8.9	11.9	6.4	9.1
Count	6	12	11	11	11

**Table 11:** Measurements (mm) of phalanx II.1. Material: IANCP569, IANCP734, IANCP758, IANCP768-776, IANCP816-817, IANCP884-892; NISP = 23.

	TL	PW	PD	SW	DW	DD
Mean	36.2	15.0	12.9	8.6	11.3	10.0
Standard Deviation	1.53	1.18	0.96	0.79	0.82	0.78
Minimum	32.2	12.8	10.4	6.8	9.5	8.9
Maximum	37.8	16.5	14.0	9.7	12.8	11.8
Count	12	10	12	14	15	15

**Table 12:** Measurements (mm) of phalanx II.2. Material: IANCP735, IANCP759, IANCP777-779, IANCP893-898, IANCP948; NISP = 12.

	TL	PW	PD	SW	DW	DD
Mean	21.2	11.7	11.0	8.5	10.4	7.5
Standard Deviation	0.87	1.22	0.61	0.53	0.60	0.58
Minimum	19.8	10.0	10.4	7.6	9.2	7.0
Maximum	22.5	13.3	12.1	9.1	11.2	8.5
Count	8	7	6	8	8	7

**Table 13:** Measurements (mm) of phalanx II.3. Material: IANCP736, IANCP803-804, IANCP806, IANCP864-867; NISP = 8.

	TL	PW	PD	SW	SD
Mean	21.9	8.5	10.3	7.5	7.9
Standard Deviation	1.86	0.43	0.49	1.05	0.41
Minimum	19.0	7.6	9.6	6.0	7.2
Maximum	24.8	8.9	11.0	8.8	8.4
Count	7	7	8	7	8

**Table 14:** Measurements (mm) of phalanx III.1. Material: IANCP737, IANCP760-761, IANCP780-787, IANCP818, IANCP899-910, IANCP949, IANCP964; NISP = 26.

	TL	PW	PD	SW	DW	DD
Mean	37.5	18.5	15.9	11.3	14.1	10.9
Standard Deviation	0.83	0.85	0.83	0.58	0.73	0.79
Minimum	36.4	16.9	14.7	10.2	12.6	9.5
Maximum	38.9	19.6	16.9	12.4	15.1	11.9
Count	12	10	9	12	11	12

**Table 15:** Measurements (mm) of phalanx III.2. Material: IANCP738, IANCP762-763, IANCP788, IANCP789-791, IANCP819-822, IANCP911-926, IANCP965; NISP = 28.

	TL	PW	PD	SW	DW	DD
Mean	27.7	15.2	11.7	10.0	12.5	8.6
Standard Deviation	1.29	0.85	1.04	0.44	0.56	0.67
Minimum	25.8	13.8	9.7	9.0	11.6	7.8
Maximum	30.4	16.5	14.3	10.8	13.6	9.6
Count	12	14	14	15	12	12

**Table 16:** Measurements (mm) of phalanx III.3. Material: IANCP739, IANCP927-929; NISP = 4.

	TL	PW	PD	SW	DW	DD
Mean	21.3	12.6	10.3	8.6	10.8	8.3
Standard Deviation	0.59	0.51	0.63	0.34	0.49	1.28
Minimum	20.7	12.0	9.8	8.4	10.5	7.4
Maximum	21.9	13.0	11.0	9.0	11.2	9.2
Count	3	3	3	3	2	2

**Table 17:** Measurements (mm) of phalanx III.4. Material: IANCP740, IANCP805, IANCP807-809, IANCP824-825, IANCP868-869; NISP = 9.

	TL	PW	PD	SW	SD
Mean	23.3	9.5	11.0	8.3	8.1
Standard Deviation	1.66	0.60	0.46	0.92	0.31
Minimum	21.5	8.7	10.2	7.0	7.7
Maximum	26.1	10.7	11.6	9.8	8.6
Count	6	9	9	8	8

**Table 18:** Measurements (mm) of phalanx IV.3. Material: IANCP741, IANCP746, IANCP792-799, IANCP826, IANCP936-944, IANCP951-953, NISP = 23.

	TL	PW	PD	SW	DW	DD
Mean	25.2	15.9	11.6	10.7	12.7	9.4
Standard Deviation	1.17	0.67	0.77	0.70	0.70	0.73
Minimum	23.6	14.7	9.6	9.1	11.2	8.5
Maximum	27.4	16.8	13.1	11.7	13.5	11.2
Count	16	15	15	16	14	14

**Table 19:** Measurements (mm) of phalanx IV.2. Material: IANCP742, IANCP747, IANCP800, IANCP827-828, IANCP945, IANCP954-958, IANCP967; NISP = 12.

	TL	PW	PD	SW	DW	DD
Mean	15.4	13.4	10.5	10.8	11.9	7.4
Standard Deviation	0.63	0.39	0.74	0.84	0.92	0.91
Minimum	14.5	12.9	9.7	10.0	11.1	6.1
Maximum	16.0	13.8	11.4	11.9	13.2	8.2
Count	4	4	4	4	4	4

**Table 20:** Measurements (mm) of adult phalanges IV.3 and IV.4. Material: Phalanx IV.3: IANCP743, IANCP748, IANCP829, IANCP959-960, IANCP968; NISP = 6, only 1 is adult. Phalanx IV.4: IANCP830.

Phalanx	TL	PW	PD	SW	SD	DW	DD
IV.3	14.3	13.1	10.3	10.1	6	11.8	8.2
IV.4	12.4	11.2	9.3	8.5	9.4	6.3	12.4

**Table 21:** Measurements (mm) of phalanx IV.5. Material: IANCP810, IANCP811, IANCP870, IANCP871, IANCP872, IANCP783.

	Tlength	PW	PD	SW	SD
Mean	20.4	8.5	10.3	7.0	7.8
Standard Deviation	1.68	0.41	0.54	0.67	0.44
Minimum	18.4	7.9	9.5	6.4	7.3
Maximum	22.4	8.9	11.2	8.1	8.3
Count	5	5	6	6	6

**Table 22:** Phalanges compared—means values for *S*. *neocaledoniae* (from Tables 8–21) compared to summary statistics (mean, [mean divided by tmtL*100], standard deviation, and range) data for *L*. *ocellata* and *A*. *lathami*, and selected individuals of various other megapode species. *Leipoa ocellata* specimens (n = 11): SAM B414, SAM B1094, SAM B5039, SAM B11482, SAM B47825, SAM B48526, SAM B48765, SAM B49461, SAM B55458, SAM B55528, SAM B58520; *Alectura lathami* specimens (n = 7): SAM B46568, MV B11471, MV B19290, QM O27852, QM O27218, QM O27843, QM O27844.

	*Sylviornis* mean	*L*. *ocellata*	*A*. *lathami*	*M*. *eremita* MV B20648	*M*. *reinwardt* ANWC 22869	*Eulipoa wallacei* USNM 558275	*Talegalla fuscirostris* KMNH 97007	*M*. *maleo* 1897.7.20.97
I.1	34.4 (21.8)	17.9 (24.3) 0.76, 16.7–19.3	22.7 (24.8) 1.14, 21.5–25.0	20.6 (32.6)	24.5 (32.4)	20.5 (34.1)	20.1 (24.5)	23.9 (27.2)
I.2	20.9 (13.2)	15.8 (21.5) 0.91, 14.6–17.2	19.0 (20.7), 1.98, 16.0–21.2	19.4 (30.7)	20.0 (26.5)	?	14.5 (17.7)	13.9 (15.8)
II.1	36.2 (22.9)	19.6 (26.6) 0.82, 18.5–21.1	23.5 (25.6) 0.89, 22.5–24.7	18.7 (29.6)	22.1 (29.2)	19.0 (31.6)	19.5 (23.8)	23.3 (26.5)
III.1	37.5 (23.8)	18.7 (25.4) 0.58, 17.8–19.8	22.7 (24.8) 0.95, 21.4–24.0	16.1 (25.5)	18.8 (24.9)	15.6 (26.0)	19.5 (23.8)	23.8 (27.0)
III.4	23.3 (14.8)	17.5 (23.8) 1.32, 15.3–20.0	20.3 (22.1) 2.12, 17.1–23.8	17.6 (27.9)	20.0 (26.5)	17.5 (29.1)	16.3 (19.9)	13.3 (15.1)
IV.1	25.2 (16.0)	14.0 (19.0) 0.53, 13.2–14.9	16.5 (18.0) 0.92, 15.3–17.5	12.5 (19.8)	15.1 (20.0)	12.8 (21.3)	13.8 (16.8)	18.0 (20.5)
Tmt L	157.8	73.7, 3.34, 67.8–79.2	91.7, 2.94, 86.8–95.4	63.1	75.6	60.1	81.9	88.0

### Results—PCA analyses of Measurement data

**Fig 12:**
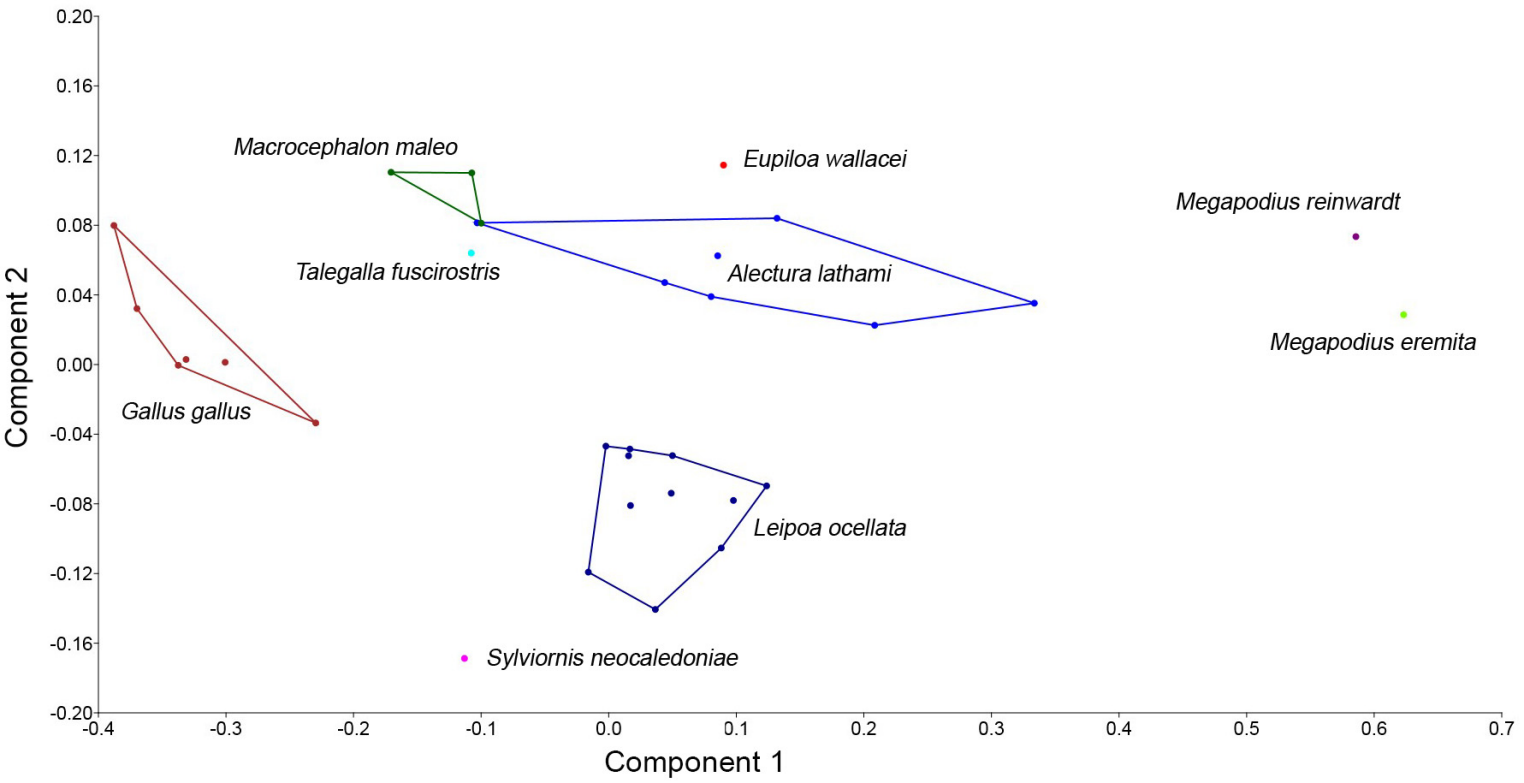
PCA plot showing separation of *S*. *neocaledoniae* from various megapodes and *Gallus gallus*. The plot is based on leg bone measurements standardised for size using femur length and selected ratios of some lengths divided by tarsometatarsus length of various galliforms compared to (see S1 File).

The datasets for measurements of femora, tibiotarsi, tarsometatarsi and phalanges (S1 File) were visualised with Principal Component Analyses conducted in PAST v3.08 [35]. Individuals for species formed discrete clusters and species were clearly segregated on the PC1 and PC2 axes by trends in two main planes (Fig 12). PC1 accounted for the majority of discrimination, determined by the Scree plot and a Loadings plot (see S4 File) with just seven variables driving the separation of the taxa in the PCA (phal I.1L/femL, phal I.2L/femL, phal III.4L/femL, phal I.1L/tmtL, phal I.2L/tmtL, phal III.4L/tmtL, phal 3.4SW/SD). A biplot of variables showed that tmtL/femL was an additional a major driver on the PC2 axis (confirmed in a Loadings plot of PC2). The broad confidence interval for tmtL/FemL on loadings plot probably relates to the fact that much of the separation relates to the single point for *Sylviornis neocaledoniae* relative to the remaining individuals. Thus in Fig 12, birds with relatively short tarsometatarsi lie more negative on the PC2 axis, so separating for example, *Macrocephalon maleo* from *Leipoa ocellata*. Simultaneously, relative length of the proximal phalanges to femur length separated birds with more elongate phalanges to the upper right on the plot from birds with relatively shorter phalanges towards the lower left. Because of the non-correlation of tarsometatarsus length and femoral length, birds with elongate proximal phalanges relative to tarsometatarsi grouped to the lower right, thus separating *S*. *neocaledoniae* and *L*. *ocell*ata from *G*. *gallus*. Lastly, the degree of dorsoventral flattening of ungual phalanx III.4, a proxy for all anterior unguals, is a major driver of separation on the PC1 axis. Taxa that are well known for great dorsoventral compression, e.g. the *Megapodius* species, are well separated to the right, with *Gallus gallus* the farthest left on PC1. Thus, *S*. *neocaledoniae*, which is well separated in the lower left of the PCA, has relative to femur length the shortest tarsometatarsus and the shortest proximal phalanges of compared taxa, and an ungual phalanx III.4 that is slightly deeper than wide at mid-length. Its ungual compression is similar to *M*. *maleo* and *Talegalla fuscirostris*, but not so enhanced as in *G*. *gallus*. In summary, *S*. *neocaledoniae* lies outside of all other megapodes, in the same direction as does *G*. *gallus*, and the best mound builders are the most distant.

### Results—Phylogenetic Analyses

**Fig 13:**
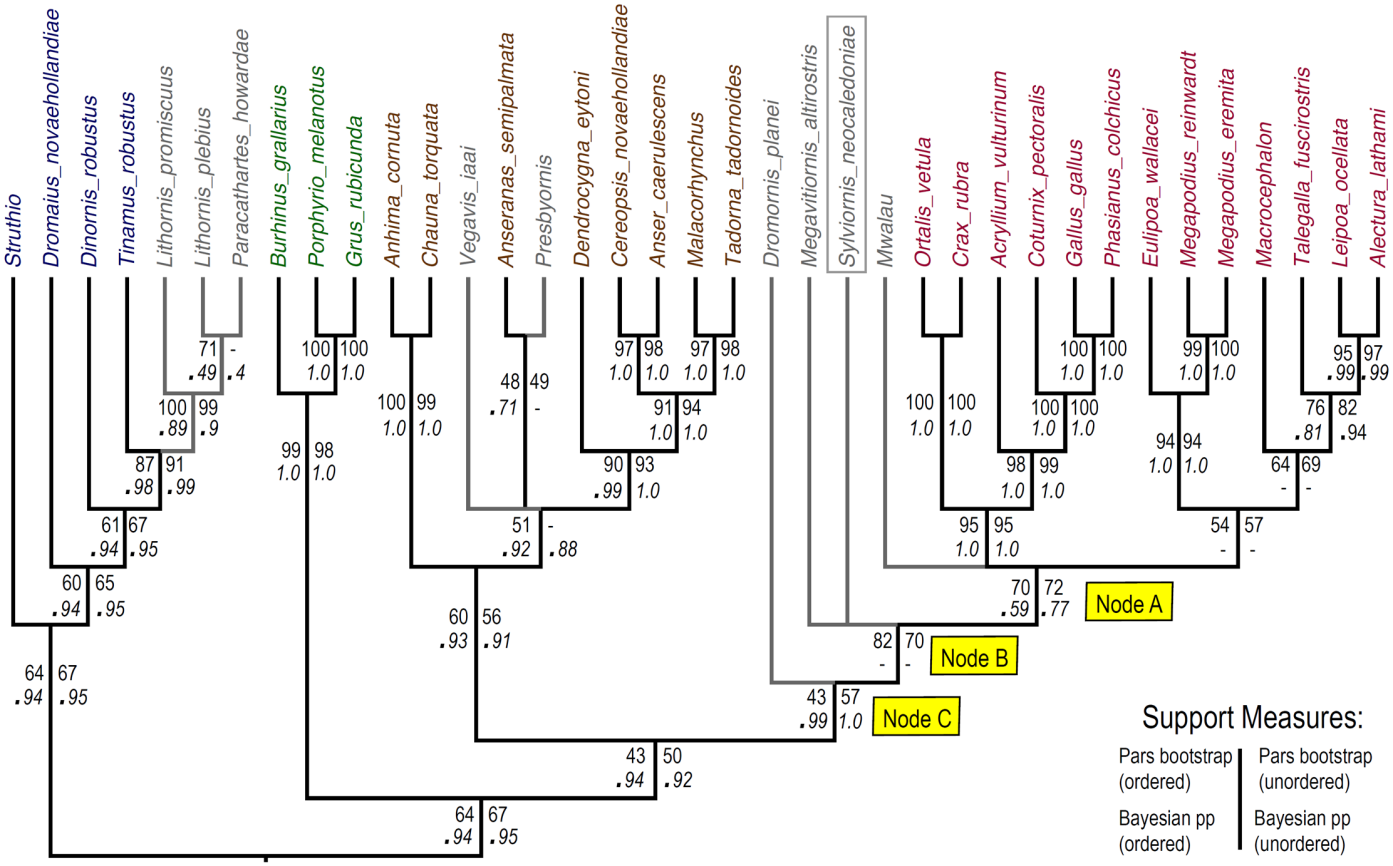
The strict consensus tree from parsimony and ordered analysis with a molecular backbone constraint. In this analysis, relationships between taxa with molecular data (living taxa and moa) were constrained, with other (fossil) taxa free to move to their optimal positions within this backbone. Tree statistics: Length 1425, Consistency index = 0.2786, Homoplasy index = 0.7214, Retention index = 0.6295. The Bootstrap and probability values of the other analyses (Parsimony, unordered, Bayesian ordered and unordered) are shown, with a dash to indicate clade not found.

Parsimony and Bayesian analyses of the morphological data with the molecular backbone constraint always placed *S*. *neocaledoniae* as a basal galliform. The parsimony analysis with ordered characters found 12 trees of length 1425; the strict consensus is shown in Fig 13, along with supports for retrieved nodes from all the "backboned" analyses. *Sylviornis neocaledoniae* along with *M*. *altirostris* fell outside of crown galliforms under all analyses, with varying support (Fig 13 Node A: bootstrap 70/72% and posterior probability 0.59/0.77, depending on whether characters were ordered/unordered). In six of the most parsimonious trees (MPTs) they were sister taxa, whereas in the other six MPTs, they formed successive branches on the stem. There is thus no evidence that either *S*. *neocaledoniae* or *M*. *altirostris* nest within crown Galliformes as close relatives of modern megapodes. Although outside the galliform crown, both taxa were robustly placed along the stem of Galliformes, by themselves in ordered parsimony analyses (Node B: bootstrap 82/70%) and together with *Dromornis planei* in Bayesian analysis (Node C, posterior probabilities 0.99/1.0). The relationships of the Holocene extinct megapode *Mwalau walterlinii* from Vanuatu were unresolved, as in six of the MPTs, it was the sister taxon to extant megapodiids, and in the remaining six MPTs, it was the sister taxon to extant galliforms. More complete specimens will be required to find resolution for this taxon. The fossil taxa *Presbyornis pervetus* and *Vegavis iaai* were recovered within crown Anseriformes with varying support (bootstrap 60/56%; pp 0.93/0.91); however, they were more strongly excluded from crown Anatidae (bootstrap 90/93%; pp 0.99/1.0). The lithornithids were retrieved with strong support as the sister group to extant tinamous, and thus deeply nested within palaeognaths (bootstrap 88/91%; pp 0.98/0.99).

**Fig 14:**
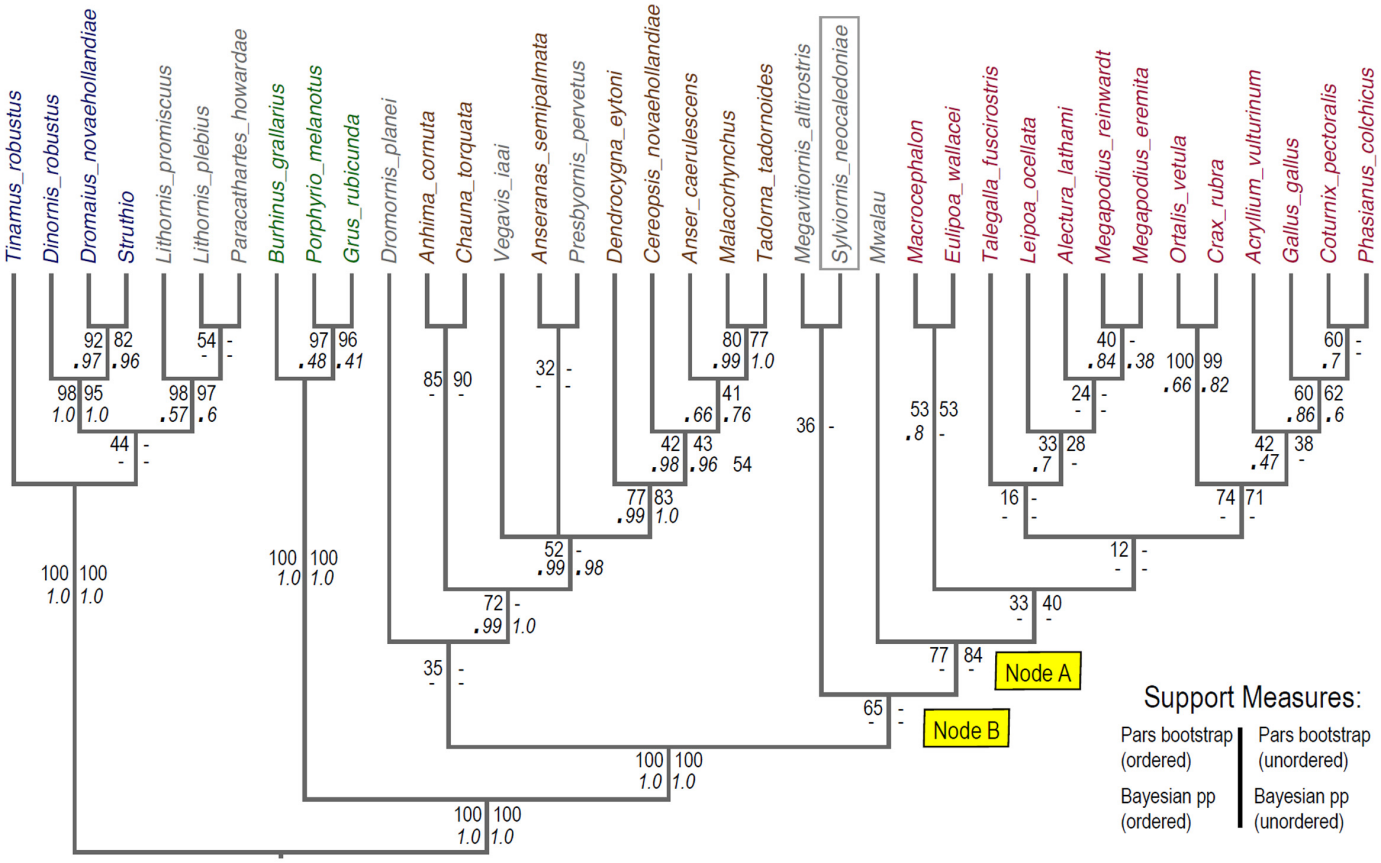
The strict consensus from parsimony and ordered analysis with only ingroup-outgroup constraints. The ingroup-outgroup constraint was (Palaeognaths ((neognaths) (galloanseres)) wherein all taxa were free to move within but not between these three clades. Three MPTs were found Length 1395, Consistency index = 0.2846, Homoplasy index = 0.7154, Retention index = 0.6404. The bootstrap and probability values of the other analyses (Parsimony, unordered, Bayesian ordered and unordered) are shown, with a dash to indicate that the clade not found. We consider this tree less plausible than the one in Fig 13 (see main text).

Parsimony and Bayesian analyses of the morphological data with only ingroup-outgroup constraints (palaeognaths ((neognaths) (galloanseres)) retrieved trees highly inconsistent with recent genetic data. In the parsimony analyses, *Sylviornis neocaledoniae* and *Megavitiornis altirostris* fall outside of crown Galliformes (Fig 14, Node A: bootstrap 77/84% ordered/unordered). However, in contrast to the molecular evidence (see Fig 13), tinamous emerge as basal palaeognaths, and megapodes are weakly resolved as a paraphyletic assemblage basal to all other living galliforms, which are monophyletic with strong bootstrap support (bootstrap 74/71% ordered/unordered). In the ordered analysis, *S*. *neocaledoniae* and *M*. *altirostris* are placed on the stem to Galliformes (Fig 14, Node B; bootstrap 65%) and *Dromornis planei* falls on the stem of Anseriformes with weak support (bootstrap 35%), while *Vegavis iaai* and *Presbyornis pervetus* fall within crown Anseriformes (bootstrap 72% and posterior probability 0.99, 1.0). The Bayesian analyses, in contrast, retrieved a monophyly of extant megapodes (consistent with molecular and morphological data) but then surprisingly placed a clade consisting of *Sylviornis neocaledoniae*, *Megavitiornis altirostris* and *Dromornis* as the sister group to megapodes (unordered) or within megapodes (ordered). These parsimony and Bayesian results are considered less plausible than corresponding analyses with the molecular backbone, and will not be discussed further.

In the context of our favoured analysis with a molecular backbone imposed, we found the following character state transformations using DelTran optimisation (for the ordered analysis) for major nodes: Galloanseres are united by 13 unambiguous apomorphies: unambiguous = this particular character transformation occurs on this node under *both* accelerated [ACCTRAN] and delayed [DELTRAN] optimisation schemes (the character in question can still exhibit homoplasy elsewhere in the tree). The following five unambiguous changes are most compelling (synapomorphic state in brackets): Character 20, CI = 1.000, 0 ==> 1 (recessus tympanicus bound ventrally by laterally projecting ala parasphenoidalis); Character 53, CI = 0.500, 2 ==> 1 (quadrate, crista orbitalis on prominent bulge laterally on proc. orbitalis); Character 60, CI = 1.000, 0 ==> 1 (mandible with two cotylae); Character 64, CI = 0.600, 1 ==> 2 (mandible, retroarticular process elongate and <2x width at base); Character 68, CI = 1.000, 0 ==> 1 (processus medialis mandibulae elongate and dorsally oriented).

*Dromornis planei* and all galliforms (Node A) are united by 13 unambiguous apomorphic changes of which the following are the most compelling (synapomorphic state in brackets): Character 3, CI = 0.667, 0 ==> 2 (premaxilla dorsoventrally deep and narrow); Character 52, CI = 0.333, 0 ==> 1 (quadrate, foramen pneumaticum rostromediale present); Character 126, CI = 0.500, 0 ==> 1 (humerus, insertion of the principle part of the tendon of M. supracoracoideus an elongate scar distal to the tuberculum); Character 248, CI = 0.500, 0 ==> 1 (tibiotarsus, distal opening of canalis extensorius, aligned transversely to the axis).

A clade consisting of *Sylviornis neocaledoniae*, *Megavitiornis altirostris* and remaining galliforms to the exclusion of remaining taxa (Node B) is supported by nine unambiguous apomorphies that all exhibited considerable homoplasy. Retention of the primitive state of these same state changes excludes *Dromornis planei* from this clade. The more significant apomorphic changes were: Character 4, CI = 0.250, 1 ==> 0 (premaxilla with flat ventral margin to decurved tip); Character 12, CI = 0.333, 2 ==> 0 (processus orbitalis lacrimale, changing from elongate to absent); Character 56, CI = 0.333, 0 ==> 1 (pterygoid articulation of quadrate, adjacent to each other to widely separated); Character 78, CI = 0.250, 0 ==> 2 (no notarium to one with 3–4 co-ossified vertebrae).

When all apomorphies (under DELTRAN or ACCTRAN) are examined though Mesquite, the following characterise *S*. *neocaledoniae* + Galliformes: Character 56 –state 1 (pterygoid articulations on the quadrate widely separated) almost unique (convergent in *P*. *pervetus* and palaeognaths); Character 78 –state 2 (notarium present with 3–4 fused vertebrae) almost unique within analysed taxa (convergent in tinamous); Character 92 –state 1 (scapula with well- developed tuberculum for attachment for ligamentum acrocoraco-procoracoideum) is almost unique (convergent in lithornithids); Character 94 –state 0 (coracoid, procoracoid with no foramen) is unique; Character 100 –state 1 (coracoid, facies articularis humeralis flat or convex) almost unique (convergent in tinamous); Character 107 –state 1 (coracoid ventral facet not prominent ventrally, directed sternally, and continuous via rounded ridge to dorsal facet) convergent in *Anhima cornuta* and *Grus rubicunda*; Character 121– state 1 (humerus, deltoid crest, caudally flat or convex) and character 132– state 1 (humerus, insertion for m. coracobrachialis caudalis forms distinct depressio insertii m. coracobrachialis caudalis at dorsal side of incisura capitis indenting the crista incisurae capitis distalis), are both almost unique, but only scored for *M*. *altirostris* as was undeterminable in the highly reduced humerus of *S*. *neocaledoniae* (convergent in tinamous); Character 167 –state 1 (carpometacarpus, ligament attachment on dorsal side trochlea carpalis for the insertion of lig. ulnocarpo-metacarpale dorsale distal to the proximal margin of processus extensorius) convergent in tinamou + lithornithids; Character 245 –state 1 (tibiotarsus, narrow incisura intercondylaris, width subequal to canalis extensorius) almost unique (convergent in *Burhinus*).

Monophyly of crown group Anseriformes (anhimids to anatids and included fossil taxa) was supported by 20 unambiguous apomorphic changes. The eight most compelling are (synapomorphic state in brackets): Character 6, CI = 1.000, 0 ==> 1 (upper bill with lamellae); Character 37, CI = 1.000, 0 ==> 1 (vomer not deeply cleft caudally); Character 38, CI = 1.000, 0 ==> 1 (vomer does not sleeve rostrum); Character 64, CI = 0.600, 2 ==> 3 (mandible, proc. retroarticularis >2 times depth at base); Character 114, CI = 0.500, 2 ==> 1 (furcula, robust and U-shaped); Character 151, CI = 0.500, 0 ==> 1 (ulna with uncompressed shaft); Character 217, CI = 0.500, 1 ==> 0 (femur, crista supracondylaris medialis long); Character 243, CI = 0.500, 1 ==> 2 (tibiotarsus, distal opening of canalis extensorius broadly overlaps condylus medialis); Character 285, CI = 0.600, 0 ==> 1 (webbing between toes present).

Nine unambiguous apomorphies united *Presbyornis pervetus* and *Anseranas semipalmata*, of which three are most compelling (synapomorphic state in brackets): Character 112, CI = 0.500, 0 ==> 1 (furcula, hypocleideum present as a low ridge); Character 158, CI = 0.667, 2 ==> 1 (carpometacarpus, fovea carpalis caudalis present and deep); and Character 190, CI = 0.500, 0 ==> 1 (femur, cranial facies adjacent to crista trochanteris deeply concave).

The fossil *Vegavis iaai* [60] is a critical calibration date in anseriforms for molecular phylogenetic analyses (e.g. [77]). Because of this, we retained this taxon but examined whether a more robust relationship for it was retrieved if we deleted other taxa with much missing data. With *Dromornis planei* excluded, the ordered and molecular-backbone constrained analysis found a single MPT where *Sylviornis neocaledoniae* was the sister taxon to *Megavitiornis altirostris* (78% Bootstrap) and formed a group with other galliforms with 100% bootstrap support. However, *V*. *iaai* was still unresolved relative to *A*. *semipalmata* + *P*. *pervetus* and Anatidae, and support for Galloanseres and Anseriformes was relatively low, with Bootstrap 63% and 62% respectively. We also excluded *V*. *iaai* to assess the effect of its considerable missing data: *P*. *pervetus* became the sister taxon to *A*. *semipalmata* with 56% Bootstrap support, and support for both Galloanseres and Anseriformes increased dramatically to 99% Bootstrap. These observations show that the largely incomplete character scoring for *D*. *planei* and *V*. *iaai* is disrupting resolution among basal galliforms and anseriforms respectively. Notwithstanding this, the data suggest that *V*. *iaai* is an anseriform in some way related to anseranatids and presbyornithids, but lying outside of anhimids or anatids.

## Discussion

### Comparisons of the post-cranial anatomy of *Sylviornis*

*Sylviornis neocaledoniae* was originally described as a ratite [10], but the affinities of this species were soon considered to lie with megapodes [11–15]. This consensus was challenged when Mourer-Chauviré and Balouet [16] placed it in a monotypic Sylviornithidae based on highly derived characteristics of the skull, including the following: its broad flattened cranium; a massive, dorsoventrally deep, laterally compressed rostrum with a large bony ornament; mandible with an elongated symphysis; and a mobile craniofacial hinge that transects the nasals early in ontogeny. However, such autapomorphies can appear in species nested within families otherwise lacking them, for example, some ratites (*Casuarius* spp.) and galliforms (e.g. *Numida meleagris*, *Macrocephalon maleo*) have bony ornament on their skull, and some galliforms have such on their rostrum (e.g. some cracids). Bill shape and size is known to vary remarkably within groups, as well shown within Anatidae, for example, with extremes seen in larger flightless taxa, such as the moa-nalos [4] and in pigeons such as the dodo [78].

To better understand the systematic implications of such autapomorphies, they need to be examined in the context of the entire skeletal morphology. We have therefore comprehensively described the post-cranial skeleton of *S*. *neocaledoniae* above, then conducted a phylogenetic analysis of a wide sample of galloanseres to examine its proposed megapode affinities. In the following, we have made detailed comparisons with various megapode taxa to assess the putative similarities, but also have drawn comparisons more widely as required. To address our secondary aim of assessing the mound building potential of *S*. *neocaledoniae*, we also used *Gallus gallus* in the comparisons to represent a non-mound building galliform.

The most obvious feature of *S*. *neocaledoniae* is its large size. It is far larger than all extant and all extinct megapodiids [12]. The giant galliform *Megavitiornis altirostris* from Fiji is substantially smaller with mean lengths of its femora, tibiotarsi and tarsometatarsi (155.2 mm vs 193 mm, 192.3 mm vs 269 mm, and 111.3 mm vs 158 mm respectively [15]. The large Australian species of *Progura* are also smaller [79].

The vertebral column is extraordinarily robust, especially in the more anterior cervicals, reflecting the much larger musculature to support and manipulate the head, which in *S*. *neocaledoniae* is massive compared to all megapodes [12]. The only putative megapodiid approaching *S*. *neocaledoniae* in cranial morphology is *M*. *altirostris*, whose skull is poorly known, but the available premaxilla, cranial and mandible fragments [15] are similar. In *S*. *neocaledoniae*, this requirement for unusually large musculature is reflected in the greatly expanded arcus vertebrae and processus spinosus of the axis and successive cervical vertebrae and in the development of a large dorsal projection on the processus articularis caudalis on vertebrae 3 to 5. We interpreted the vertebral count as 20 presacral vertebrae and thus the same as for all galliforms. These include 12 cervicals, two cervicothoracic (numbers 13, 14) and six thoracic vertebrae, i.e. those with ribs attached. Three thoracic vertebrae (17–19) are fused as a notarium, which is one less than for all extant megapodes examined, wherein usually four (numbers 16–19) are fused as a notarium. However, there is some variation, as one *A*. *lathami* examined, QM O.27844, has a notarium including just three vertebrae, numbers 16–18 and Poplin and Mourer-Chauviré [12] reported that species of *Megapodius* have three. It is inferred that the four sternal ribs articulated with costal ribs on vertebrae 16–19. The whole vertebral series displays reduced pneumatism concordant with the flightless nature of *S*. *neocaledoniae* and the lack of need to minimise mass.

The pelvis of *S*. *neocaledoniae* is poorly represented in our material precluding any useful comparisons. We cannot ascertain how broad the pelvis was as the sole synsacrum lacks complete lateral processes and there are no well-preserved ilia. But the available fragments allow estimation of pelvis length assuming proportions similar to *Leipoa ocellata*, which is the extant megapode closest to *S*. *neocaledoniae* in the principal component analyses. The synsacrum IANCP554 is 79.2 mm long from the anterior margin of facies articularis cranialis to the caudal side of the last and biggest transverse process articulating with the ilia, which is 266% larger than *L*. *ocellata* SAM B.11482 with a corresponding length of 29.7 mm. The ischial acetabular fragment IANCP557 is c. 50 mm wide from the top of the facies antitrochanterica to the opposite side of the acetabulum, or is 324% larger than in the same specimen of *L*. *ocellata* (15.4 mm). If the pelvis of *Sylviornis neocaledoniae* was 266–324% larger than that of *Leipoa ocellata* SAM B11482 (total length 106.5 mm), a total length in the range 283 to 345 mm is possible. However, IANCP554 is 34 mm across the major articular processes with the ilia, versus 19.5 mm or only 174% larger, indicating perhaps a relatively narrower pelvis in *S*. *neocaledoniae*. This and that the foramen ilioischiadicum is longer than half ischial length, and so more elongate than all megapode species and all galliforms examined, indicate that the pelvis of *S*. *neocaledoniae* is not just an enlarged version of *L*. *ocellata*. It is, however, much smaller than that indicated in the sketch provided by Balouet ([14], Fig 5) wherein it was depicted as three times longer than the femur.

The pectoral girdle of *S*. *neocaledoniae* differs greatly from those of volant fowl and clearly indicates that it could not fly. All elements have reduced robustness relative to those in volant forms and are proportionately smaller using femur length as a proxy for body size, but the distal wing elements are relatively more reduced in length compared to the more proximal ones. No extant megapodes are flightless and the only undoubted candidate among extinct forms is *Megavitiornis altirostris* [15, 31]. The giant extinct species of *Progura* from Australia all display elongate and robust wing elements and sterna with a deep carina, ones not relatively smaller than those of smaller volant surviving taxa ([15, 79], THW unpubl. Data). Given these caveats, however, some comparisons can be made. The available fragments of the sternum reveal little other than that *S*. *neocaledoniae* has four costal articulations which typifies galliforms in general, although the caudal two were somewhat reduced in size. The sternum appears to have a sternal basin that is rectangular in section, but the extent of loss or reduction of the carina is unknown, although Balouet [14] depicted a carina in his reconstructed skeleton. The coracoid sternal articulation was limited to a small flat facies, very widely separated, located at the junction of the flat ventral sternal basin and its ascending lateral sides.

The coracoid is relatively elongate, which is unsurprising as its length relates to thorax size and the need for it to connect the sternum to the humeral pivot on the side of the bird. It is, however, very gracile concordant with the markedly reduced wing and is very similar in proportions and shape to that of *Megavitiornis altirostris*, sharing with that taxon a markedly reduced processus acrocoracoideus and the loss of the processus lateralis [15]. The considerable reduction of the acrocoracoid, which is associated with the loss of the ability to fly as noted by Poplin and Mourer-Chauviré [12], has resulted in the facies articularis clavicularis becoming a rugose zone on the medioventral facies of the acrocoracoid that is aligned roughly parallel to the shaft, rather than in a dorsoventral plane at about right angles to the shaft as in megapodes. The overall form of the omal end of the coracoid in dorsal view is that the cotyla scapularis, facies articularis humeralis and acrocoracoid form a shallow C-shape, rather than the marked, near right angle, that characterises galliforms. The acrocoracoid overhangs the shaft ventrally, which Holman [80] stated does not occur in megapodiids or cracids, but this is variable and occurs in *L*. *ocellata*, *M*. *reinwardt*, *T*. *fuscirostris* and *Progura naracoortensis* among megapodes we examined. *Sylviornis neocaledoniae* differs from *M*. *altirostris* by a more sternally-elongate ventral projection of the processus acrocoracoideus over the shaft, which in medial view starts level with the facies articularis scapularis: in *M*. *altirostris*, the projection commences level with the cranial end of the facies articularis humeralis. The thick straight shaft with only a rudimentary processus procoracoideus that lacks a foramen nervi supracoracoidei is typical of galliforms [12]. The omal end of the impressio musculus sternocoracoidei is markedly elevated relative to the shaft more omally, which is a point of difference with all extant megapodes examined and *P*. *naracoortensis*. *Sylviornis neocaledoniae* has pneumatic foramina in the lateral side of the impressio musculus sternocoracoidei. Such pneumatism is variable in Megapodiidae [80], but is well-developed in *P*. *naracoortensis*, *L*. *ocellata* and *M*. *reinwardt*.

The scapula is typical of galliforms in gross morphology, including the presence of a well-developed process for ligamentum acrocoraco-procoracoideum on the acromion, which is a feature characteristic of galliforms and lithornithids (as styloid acromion in Houde [28]), that is absent in all anseriforms, and seems to be associated with the absence of a processus procoracoideus on the coracoid. Also typical of galliforms, it lacks a tuberculum coracoideum, with instead a flat facies articularis coracoideum. However, it has a large pneumatic foramen ventromedial to the facies articularis humeralis, a feature found only in megapodes and cracids among galliforms [80], and which is not present in anseriforms. In our specimens, this foramen was larger than in those previously reported from Ile de Pins [12]. We did not recognise any clavicula fragments in our sample, and it is likely that the furcula is reduced to two separate clavicle slivers that are closely applied or occasionally fused to the acrocoracoid in life, given the rugose nature of the facies articularis clavicularis, see Balouet ([14]: Plate 4A,B). A similar reduction in clavicles is seen in the parrot *Strigops habroptilus*, which is also notably flightless (pers. observ. THW).

The wing retains a normal skeletal set of bones, although all are much reduced from their volant original form. The humerus, while elongate, is relatively much shorter than in volant forms when compared to the leg elements. Its proximal and distal extremities are also markedly reduced compared to all volant galliforms, including *Progura naracoortensis*. Nevertheless, several features of the humerus unambiguously show the galliform nature of *S*. *neocaledoniae*. These include the insertii m. coracobrachialis caudalis being located in the dorsal end of the incisura rather than on the tuberculum ventralis, an elongate impressio musculus supracoracoideus forming a shallow scar on the dorsal facies and a small and low tuberculum dorsale. However, the attachment of m. latissimus dorsi on the dorsal side of the caudal shaft surface is characteristic of megapodes within galliforms: in all others it inserts on the ventral side of the shaft surface. The absence in *Sylviornis* of the tuberculum intermedium [71], that characteristically for galliforms extends from the caput humeri to close the incisura capitis dorsally, may relate to reduction of the caput and proximal end generally. Similarly, caution is warranted in interpreting the observation that the fossa pneumotricipitalis dorsalis, fossa II of Holman [80], is only a shallow sulcus distal to the dorsal half of the caput, as in megapodes and cracids, rather than the deep one of many galliforms. However, *S*. *neocaledoniae* shares the small fossa pneumotricipitalis ventralis and especially the thickened and broad caudomedial margin of this fossa solely with megapodiids among galliforms [12, 80]; such features and also the dorsal attachment of m. latissimus dorsi are likely plesiomorphic in galliforms. Distally, the very flattened fossa m. brachialis and poorly separated condyli [12] are undoubtedly related to the flightless nature of the bird.

The ulna of *S*. *neocaledoniae* is slender with the olecranon and tuberculum carpale much reduced [12] as expected of a flightless bird. The markedly dorsoventrally compressed ulnar shaft is typical of galliforms as is its well-marked impressio brachialis. The ulna differs from that of all megapodes by the presence of an enclosed incisura radialis, but this may relate to the marked dorsoventral compression of the shaft immediately distal to the proximal end, which is probably the result of loss of volancy.

The carpometacarpus of *S*. *neocaledoniae* is markedly reduced in size compared to that of volant megapodes, e.g. it is much smaller than that of *P*. *naracoortensis*, which is a much smaller bird. Associated with this is a marked reduction in relative size of the trochlea carpalis and of the processus pisiformis as noted previously [12]. It retains a distinct processus alularis with an articular facet for phalanx digiti alulae, so the wing did have a pollex, and distally two facets remain for the distal phalanges of digits II and III. Many galloanseres are characterised by a ligamental notch in the dorsal (= external) rim of trochlea carpalis. In megapodiids and cracids, the dorsal rim continues distally of the notch [80], with often the only indication of a notch, in caudal view, is a marked swing ventrally of the crest, before its continuation more distally, whereas in other galliforms, e.g. *G*. *gallus*, it ends abruptly at this notch. The very abbreviated trochlea carpalis, especially the dorsal rim, of *S*. *neocaledoniae*, makes assessment of this feature impossible. The presence of large foramina, previously noted by Poplin and Mourer-Chauviré [12], penetrating the base of the processus extensorius in *S*. *neocaledoniae* appears to be unique. Dorsally this foramen appears to have housed a vessel exiting in a distal direction, whereas that on the ventral surface is larger and possible pneumatic in nature. No similar foramina were seen in megapodes, although a small vesicular foramen is present ventrally at the base of the processus extensorius in *P*. *naracoortensis*. The carpometacarpus of *Sylviornis neocaledoniae* is similar to those of megapodes and *Acryllium vulturinum* in lacking a processus intermetacarpalis, whereas those of phasianids and cracids have one [12, 80]. In cracids, the processus intermetacarpalis is very small but distinct, and while Holman [80] reported that this is the case in *Alectura lathami*, our observations of this megapode found it like others and it seems likely he saw a specimen where the flexor attachment was a bit more rugose than normal. Uniquely among galliforms, the fossa for the origin of m. abductor indicis [72] is greatly enlarged in *S*. *neocaledoniae* and extended over the ventral facies of the proximal parts of os metacarpale minus and os metacarpale majus by a flange of bone. Poplin and Mourer-Chauviré [12] noted this flange, explaining it as incomplete fusion of the minor metacarpal. However, we interpret the presence of the flange and the greatly enlarged fossa to mean that the m. abductor indicis was hyper-developed in *S*. *neocaledoniae* implying some function for the distal wing despite the bird being flightless. This is supported by the large protuberant tuberosity ventrally on the proximal end of os metacarpale minor for the origin of m. flexor digiti III, whose insertion in the more distal wing is responsible for its flexion, and that the os metacarpale majus has four large depressions on its dorsal surface for the insertion of remiges primarii. Also, on well-preserved ulnae papillae remigales are present. Together these features suggest that the primary and secondary feathers were large and could be manipulated for display. This wing was thus relatively larger than that of the kiwi *Apteryx* sp., which are functionless and buried within the plumage, but not so large as in the large palaeognaths *Struthio camelus* and *Dromaius novaehollandiae*, where the elongate wing bones allow for holding display feathers far out from the body. In *S*. *neocaledoniae*, the wings may have been like they were in the dodo [6, 78], with small but distinct feather tufts able to be protruded from the body plumage.

The leg bones, while very much larger and stouter, were found to have greatest similarity to those of megapodes by Poplin and Mourer-Chauviré [12]. The femur is very much larger than that of any other galliform, but is proportioned similar to that of megapodes. It shares with all galliforms the presence of a distally short trochanter femoris with a well-developed fossa trochanteris and a caudocranially convex facies antitrochanterica. It shares with megapodes a trochanter whose crista trochanteris is well elevated above the shaft, whereas it is lower in all other galliforms except some meleagridids [80]. It shares with the megapode species in *Progura*, *Leipoa*, *Alectura*, *Megapodius*, *Eupiloa*, but not *Megavitiornis altirostris*, *Talegalla fuscirostris* or *Macrocephalon maleo*, pneumatic foramina entering the trochanter cranially, as in part noted previously [12]. The absence of a pneumatic foramen entering below the facies articularis antitrochanterica caudally is shared with *L*. *ocellata*, species of *Megapodius*, *A*. *lathami*, *M*. *altirostris* and *M*. *maleo*, but is in contrast to *P*. *naracoortensis* and *T*. *fuscirostris* which have a large foramen. The accentuation of the marks for certain insertions, e.g. those for m. gastrocnemialis lateralis and the ligamentum collaterale lateralis probably relate to the size of the bird. A major difference from femora of compared galliforms is the form of the fossa poplitea, which is restricted to the medial half of the bone and forms a deep, steep-sided pneumatic pocket. In *Gallus* and megapodes, it is shallow and, moreover, is lined proximally by a well-marked scars for muscle insertions. In *S*. *neocaledoniae*, the insertion for ligamentum cruciatum craniale, which lies laterad of the fossa poplitea, is relatively larger than in the smaller extant galliforms and limits the lateral extent of the fossa. This ligamental attachment is however of similar size in the large extinct *P*. *naracoortensis* and its base is perforated by foramina.

The tibiotarsus of *S*. *neocaledoniae* is very much larger than that of any other galliform including the extinct *Megavitiornis altirostris*. It is more robust than other megapodes with shaft width 8% of length compared to 6.3% in *Leipoa ocellata* and 5.9% in *Alectura lathami* (S1 File). Typical of galliforms it had a proximally low crista cnemialis cranialis that was proportionally shorter than in extant megapodes [12], unconstricted base of the cnemial crests, distal end with condylus medialis not much inflected medially, the sulcus musculus fibularis is on the lateral facies although its retinaculi are low in contrast to the prominent ones in megapodes, centrally located sulcus extensorius with canalis extensorius opening distally towards the gap between the condyles, the distal margin of the pons supratendineus slopes proximomedially in cranial aspect, and a narrow incisura intercondylaris. The condylus medialis projects more than 10% cranially of the condylus lateralis in *S*. *neocaledoniae* and *Megavitiornis altirostris*, unlike in all megapode species examined (species of *Megapodius*, *Talegalla*, *Aepypodius*, *Macrocephalon*, *Eulipoa*, *Leipoa*, and *Alectura*) and other galliforms, where the condyle cranial height was more nearly equal. This is a result of a relatively lesser cranial projection of the condylus lateralis in *S*. *neocaledoniae*, as the condylar width versus depth is about equal, slightly wider than deep in most megapodes, but notably deeper than wide in *G*. *gallus*. The proximodistal length of the pons supratendineus is shorter than its width, as in *M*. *altirostris*, *P*. *naracoortensis*, *M*. *reinwardt*, and *T*. *fuscirostris*. The epicondylaris medialis is low and not sharply projecting, less so than in any megapode examined, including *M*. *altirostris* and *P*. *naracoortensis*. Similarly, the impressio ligamentum collateralis medialis is very low compared to all megapodes, but not dissimilar to that of *G*. *gallus*. Both the poorly developed impressio ligamentum collateralis medialis and the low epicondylaris medialis probably relate to a lesser strength to dig or scratch earth. Another difference from all megapodes examined is that the lateral wall of the incisura intercondylaris is sloped laterally, not forming a face nearly parallel to the other side of the incisura as in, e.g., all megapodes, which may relate to the lower cranial projection of this condyle.

The tarsometatarsus of *S*. *neocaledoniae* is large and robust and relative to femoral length shorter than in any megapode except *Megavitiornis altirostris*, 82% femur length vs 72% respectively (data herein, [12, 15]). There is no evidence of a spur cone such as typifies males in species of Phasianinae [80]. As for all megapodes and *M*. *altirostris*, trochlea metatarsi II has about equal distal extent as trochlea metatarsi IV or extends slightly farther distal. Holman [80] reported that in *L*. *ocellata* and *A*. *lathami*, trochlea metatarsi II was slightly elevated above trochlea metatarsi IV, but in all our specimens, trochlea metatarsi II extended slightly more distad of IV. All other galliforms have trochlea metatarsi II more proximad of trochlea metatarsi IV. As in most galliforms and all megapodes it has a single hypotarsal canal, but the depth of its hypotarsus is less than for all megapodes. While it shares with all megapodes a convex anterior facies to the distal shaft, it differs from them in that the facet in fossa metatarsi I does not project mesad of the shaft, and it has only a minute foramen marking the plantar exit of the foramen vasculare distale, although, dorsally, it is large and extends via a broad canalis interosseous distalis, to the incisura intertrochlearis lateralis [12]. Moreover, as noted previously [12], the shaft is relatively craniocaudally thicker mesad of the extensor sulcus than in all megapodes, wherein it is often compressed to a thin flange, e.g. in species of *Talegalla*, *Aepypodius*, *Megapodius*, *Leipoa*, *Alectura*, and *Macrocephalon*. This relates to the excavation of the fossa parahypotarsalis medialis which is shallower in *S*. *neocaledoniae* than in any megapode. Also, the tuberositas m. tibialis cranialis is weakly developed and does not project above the medial margin of the extensor sulcus as it markedly does in species of *Megapodius* and to a lesser extent in *A*. *lathami*. *Sylviornis neocaledoniae* differs from *M*. *altirostris* in having a more elongate tarsometatarsus with a much shallower fossa metatarsi I with no facet, and a far shallower fossa parahypotarsalis medialis. However, the shallow fossa metatarsi I supported a relatively elongate metatarsal 1 that articulated with phalanx 1 of digit 1 relatively low on the tarsus, which is characteristic of megapodiids and cracids, whereas digit 1 of phasianids starts higher on the tarsus [12]

The toes had the usual avian phalangeal formula (2, 3, 4, 5). The phalanges of *S*. *neocaledoniae* are short relative to the tarsometatarsus length and robust or broad for their length (Tables 8–21 above, [12]). The length of phalanx I.1 is almost the same length as that of phalanx III.1 (Tables 9 and 14), which distinguishes Megapodiidae and Cracidae from other galliforms [12]. The ungual phalanges are laterally compressed and short, whereas those of all megapodes are more elongate and especially so relative to the length of the tarsometatarsus (see Table 22). *Talegalla fuscirostris* is the megapode wherein phalanx length as a proportion of tarsometatarsus length most nearly approaches that for *S*. *neocaledoniae*, particularly for phalanges II.1 and III.1. However, in terms of relative length of ungual phalanges, those of *Macrocephalon maleo* (I.2 and III.4) are the most similarly short, and this taxon is also most similar to *S*. *neocaledoniae* in sharing lateromedially compressed unguals. The extinct Australian taxon *Progura naracoortensis* [74] also has laterally compressed and relatively short unguals.

The data obtained here allows comparison with those previously published for *S*. *neocaledoniae* from Ile des Pins [12]. That material is more sparse and more fragmentary, but where comparative measurements are possible, such as for lengths of ulnae, radii and carpometacarpi, our data show complete overlap of measurements. The exceptions are where Poplin and Mourer-Chauviré [12] estimated lengths for tibiotarsi (320 mm) and tarsometatarsi (172 mm), which far exceed our mean values of 269 mm and 158 mm respectively. Those authors assumed proportions for *S*. *neocaledoniae* were similar to those of *Alectura lathami*. The data suggest that the birds on Ile des Pins and at Pindai Peninsula were the same size and, as described by Poplin and Mourer-Chauviré [12], they do not differ in any major way.

We estimate the mass of *S*. *neocaledoniae* as 27–34 kg. This is slightly smaller than the 30–40 kg estimate in Steadman ([2]: 292), but nevertheless confirms it as the most massive neognath in the Pacific region. It is larger than the flightless gruiform *Aptornis defossor* (mean 18.9 kg, range 14.9–22.4 kg) or the giant goose *Cnemiornis calcitrans* (mean 18.3 kg, range 16.4–20.1 kg) of New Zealand [3]. It was exceeded in size by moa (Dinornithiformes) New Zealand and the emus and cassowaries (Casuariidae) in Australia-Papua region. However, it was not the largest galloanserine, as all mihirung birds (Dromornithidae) of Australia and the gastornithids of the Northern Hemisphere were larger [24].

**Fig 15:**
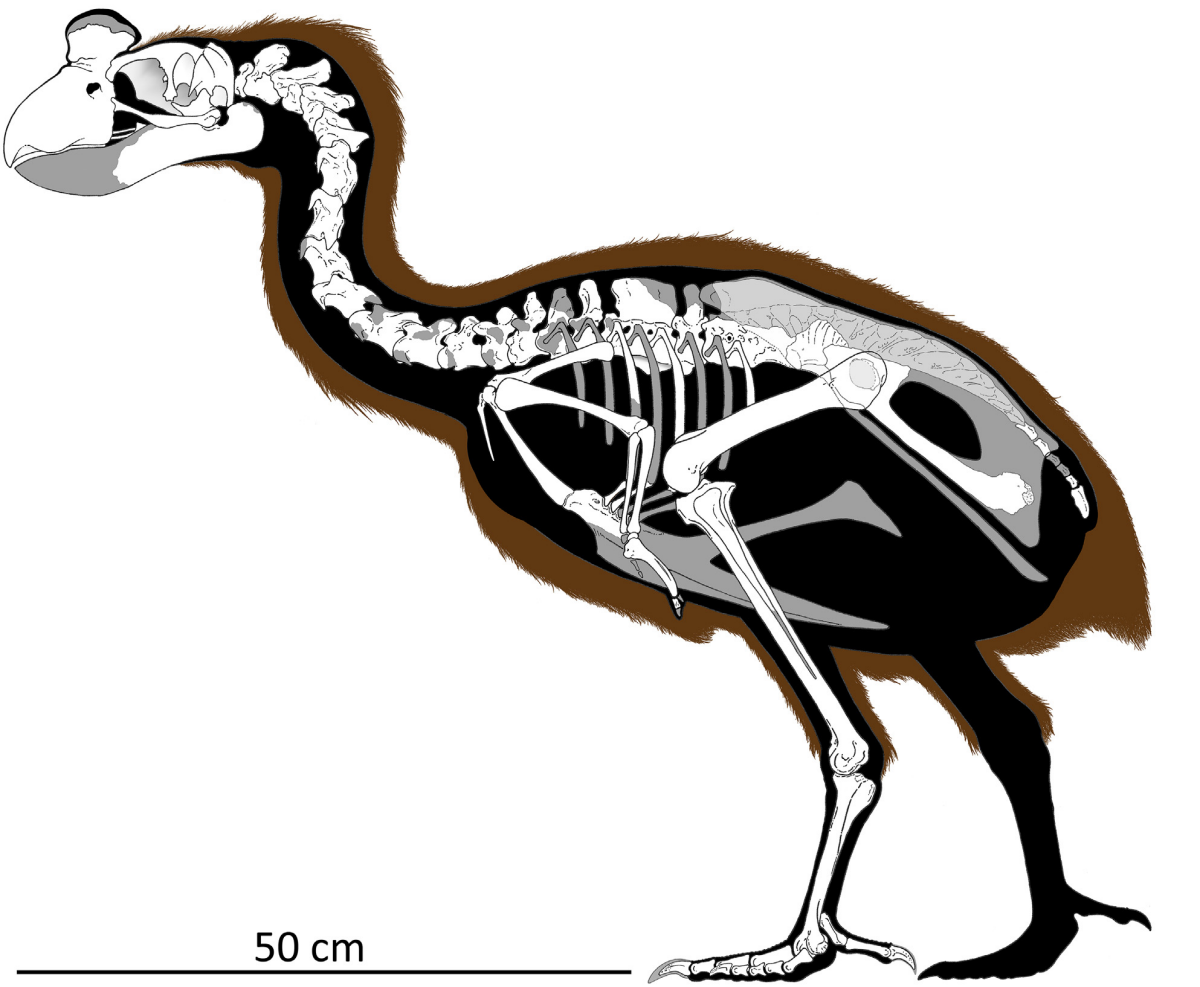
A skeletal reconstruction for *Sylviornis neocaledoniae* in a resting pose. The missing skeletal parts are estimated (shaded bones), with the pelvis based on proportions of *Leipoa ocellata*. The bill rhamphotheca is not reconstructed so as to not obscure the underlying skeletal morphology.

In summary, *S*. *neocaledoniae* was larger than any extant or fossil galliform. It also had a more robust and larger head and concomitant with that a more robust vertebral column. Its large size was associated with flightlessness as evidenced by markedly reduced pectoral elements and together these obscure comparisons of features of phylogenetic utility with other galliforms. Many of the similarities with megapodes are plesiomorphic in nature as concluded by Mourer-Chauviré and Balouet [16]. Its tarsometatarsi are short and its toes were much shorter and more robust than megapodes. Therefore, in sum, *S*. *neocaledoniae* may be reconstructed as a heavyset (27 to 34 kg), short-legged bird with a massive head standing some 85 cm high (Fig 15), quite unlike any extant galliform. As in the dodo, short tufted wings would have extended from the plumage, which together with the knob-like osseous ornament to the bill that likely supported brightly coloured skin as in cracids [81], very likely was used in complex sexual displays.

### Mound-building ability of *Sylviornis neocaledoniae*

The skeletal morphology shows that *S*. *neocaledoniae* has, compared to most megapodes, a smaller hypotarsus, reduced muscular insertion areas plantarly, and correspondingly less insertion area dorsally on the tuberositas m. tibialis cranialis for ligaments flexing the digits, which in sum imply a very reduced musculature for retracting the digits. To interpret the functional constraints that this leg bone morphology has on its digging ability, we conducted a Principle Components Analysis using 56 variables based on measurements of *S*. *neocaledoniae*, *Gallus gallus* and several megapode species that represented the range of mound-building ability in the family. The taxa were widely separated on plots of PC1 and PC2, with the separation mainly driven by the relative lengths of the tarsometatarsus, proximal phalanges and unguals and by the degree of dorsoventral flattening of the ungual phalanx III.4, with species of *Megapodius* and *Gallus* at opposite ends of the range. *Sylviornis neocaledoniae* lay outside of all megapodes, as did *G*. *gallus*. These observations can be interpreted in terms of function. If a line is drawn from *Megapodius* to *Gallus* on the PCA plot (Fig 12), it delimits a trend from obsessive diggers such as *Megapodius*, species of which construct the largest mounds of any megapodes, although others burrow in large communal nesting grounds in volcanically heated earth [82], to *G*. *gallus*, that does not construct mounds, but does scratch whilst feeding. The two megapode species that do not construct mounds for nesting, *Macrocephalon maleo* and *Eulipoa wallacei*, nest in shallow burrows or pits dug into beach sands [82]. Both have relatively unflattened unguals and *M*. *maleo* has a relatively shallower fossa parahypotarsalis medialis than other megapodes, but one that is still much deeper than in *S*. *neocaledoniae*, and both have a relatively elongate tarsometatarsus and elongate proximal phalanges and unguals, which together confer a better digging ability for them than in *S*. *neocaledoniae*. In conclusion, *S*. *neocaledoniae* could, at best, scratch in a manner not dissimilar to a chicken and would not have been capable of digging the large nesting mounds that characterise certain megapodiids.

*Sylviornis neocaledoniae* has been identified as a possible maker of large mounds or tumuli on La Grande Terre and Ile des Pins (e.g., [2, 18–20]). However, there is no evidence of association of this species with these mounds and eggshell has not been found in them, despite the calcareous nature of at least those on Ile des Pins [20]. Given that our analyses show that *S*. *neocaledoniae* has no enhanced morphological adaptations for digging, such as those displayed by certain megapodes, it is most unlikely that it is responsible for these tumuli. Its relationships, as inferred from its phylogenetic position as the sister group to crown galliforms, makes it further unlikely to have been an ectothermic incubator, as if it was, this trait would have to have evolved twice on the galliform stem. Instead these tumuli are likely to be formed by an interplay of vegetation and erosion as suggested for similar mounds elsewhere [83–85]. Even if mounds were associated with eggshell, then the largest of all *Megapodius* species, the New Caledonian endemic and extinct *M*. *molistructor* Balouet and Olson, 1989 [13] would be a more likely contender for their builder.

### Insights into relationships of *Sylviornis neocaledoniae*

Our phylogenetic analyses examined fossil taxa within the phylogenetic framework of extant species as well established from analyses of molecular data for Aves and Neoaves [56, 58], Palaeognathae [40, 64, 65], Galliformes [65], and Anseriformes [66–68]. Taxa known only from morphology (i.e. fossils) were free to move to their optimal positions within this molecular backbone (extant taxa and *Dinornis robustus*) according to the phylogenetic signal in the 285 morphological characters scored. Within both parsimony and Bayesian analyses (Fig 13), *Sylviornis neocaledoniae* was outside extant (crown) Galliformes. In some analyses, it associated with *Megavitiornis altirostris* as its sister taxon, or in other analyses, these taxa formed a grade on the galliform stem. There is thus no evidence that either *S*. *neocaledoniae* or *M*. *altirostris* nest within crown Galliformes as close relatives of modern megapodes. This supports the decision by Mourer-Chauviré and Balouet [16] to remove *Sylviornis* from Megapodiidae and erect the new family Sylviornithidae. On the basis of our analyses, we here transfer *M*. *altirostris* of Vitilevu, Fiji from Megapodiidae to Sylviornithidae.

Mourer-Chauviré and Balouet [16] identified several autapomophies to support their referral of *S*. *neocaledoniae* to Sylviornithidae. However, we have some reservations about the use of autapomorphies to remove a highly derived taxon from within a group as, for example, large size, attributes of flightlessness, and specialised skull morphology do not preclude large flightless Hawaiian moa-nalos from being nested within Anatidae [4], and the ornament observed on the premaxilla of *S*. *neocaledoniae* is variably present among other galloanseres. Furthermore, some of these proposed autapomorphies are present in other, potentially related, taxa prompting us to reassess the significance of them. They considered that *Sylviornis neocaledoniae* was the only bird with a craniofacial hinge forming a diarthrosis of a ginglymus or hinge type, but *Megavitiornis altirostris* (here considered to be a sylviornithid), the gastornithid *Diatryma gigantea* Cope, 1876 [86], and the dromornithids, all have such a craniofacial hinge. Moreover, most parrots have a non-ossified craniofacial hinge where the premaxilla abuts the cranium, sometimes within a sulcus, especially in those with larger bills such as cacatuids. In the extinct taxa, function cannot be observed directly, but for the parrots, this hinge-type is associated with extreme mobility of the premaxilla to widen the gape and to facilitate manipulation of food items in their mouth. In *S*. *neocaledoniae*, the premaxilla encloses dorsally a large cavity in the bill which presumably contained a large tongue. With a large tongue and an unossified highly mobile craniofacial hinge, the bird was well adapted to manipulate objects in its mouth, and the most likely food items requiring this would be fruit or seeds. Among megapodes, *M*. *maleo* has a craniofacial hinge that approaches the synovial joint seen in *S*. *neocaledoniae* and which is associated with a much heavier bill than most megapodes. The effect of the hinge-like craniofacial hinge in *S*. *neocaledoniae* results in the unusual situation where the nasal is divided during ontogeny into two ossification centres, one caudal to and one rostral to the joint. This feature is also observed in dromornithids.

Another autapomorphy Mourer-Chauviré and Balouet [16] noted was that the cranial flexure, i.e., the occipital condyle, was uniquely situated just caudal to the basipterygoid processes, but this is also a feature of dromornithids [23, 24], here recovered near *S*. *neocaledoniae* as a more basal stem galliform. Moreover, a similar conformation appears in anhimids. *Sylviornis neocaledoniae* has an apparently unique trait where a descending lobe of the lacrimal projects rostroventrally to articulate via a small facet with the side of the nasal rostral to the craniofacial hinge, thereby providing lateral stability to the large rostrum. However, we note that in both *M*. *maleo* and *E*. *wallacei*, which have relatively larger rostra than other megapodes, the lacrimal configuration departs from that of other megapodes and galliforms. In them, the lacrimal is completely separated from the frontal and extends down the lateral side of the nasal rostral to the hinge, rather than being synostosed to the lateral edges of the frontal caudal to the hinge as in most galliforms. Thus, in birds with robust rostra, the lacrimal displays considerable variation in how it articulates with the rostrum or not, and so may reflect functional rather than phylogenetic constraints.

The skull of the giant Fijian sylviornithid *Megavitiornis altirostris* is not as well-known as that of *S*. *neocaledoniae*, but it shared the same broad cranium with a similar hinge. The nasal within the rostrum had a robust descending process but enclosed larger nares, so was less extensively fused with the premaxilla [16], but the degree of fusion ventrally between the nasal and maxilla cannot be judged because of breakage [15].

Our analysis finds that nine unambiguous apomorphies unite *Sylviornis neocaledoniae* and *Megavitiornis altirostris* in a clade with remaining galliforms to the exclusion of *Dromornis planei* (Fig 13, Node B). Further analysis showed that several apomorphic changes are compelling with limited convergence elsewhere: 1, the loss of the processus orbitalis lacrimale (Character 12); 2, separation of the pterygoid articulation on the quadrate (Character 56, convergent in *Presbyornis* and palaeognaths); 3, the co-ossification of 3–4 vertebrae to form a notarium (Character 78, convergent in tinamous); 4, scapula with well-developed tuberculum for attachment for ligamentum acrocoraco-procoracoideum unique (Character 92, convergent in lithornithids); 5, coracoid, procoracoid with no foramen (Character 94); 6, facies articularis humeralis of coracoid flat or convex (Character 100, convergent in tinamous); 7, humerus, crista deltopectoralis caudally flat or convex (Character 121) and (8) insertion for m. coracobrachialis caudalis on humerus forms a distinct depressio insertii m. coracobrachialis caudalis at the dorsal side of the incisura capitis that indents the crista incisurae capitis distalis (character 132), are convergent in tinamous; 9, carpometacarpus, ligament attachment on dorsal side trochlea carpalis for the insertion of lig. ulnocarpo-metacarpale dorsale distal to the proximal margin of processus extensorius (Character 167, convergent in tinamou + lithornithids); 10, and tibiotarsus with a narrow incisura intercondylaris where width is subequal to that of the canalis extensorius (Character 245 convergent in *Burhinus*). It is interesting that four of these (3, 6, 7, 8, 9) are convergent in tinamous and galliforms, and two (4, 9) are convergent in lithornithids, here recovered as the sister taxon of tinamous. This is in accordance with the noted similarity of the superficially fowl-like tinamous to galliforms (e.g. [28, 87]) and the similarities of lithornithids and tinamids [28]. The convergent loss of flight within palaeognaths [40] suggests that the lithornithid or tinamid condition is probably more representative of the primitive state in palaeognaths. These points justify the inclusion of lithornithids and tinamous in the furthest outgroup.

The dromornithid *Dromornis planei* was found to be the sister group to *S*. *neocaledoniae* and all other galliforms with significant Bayesian support united by 13 unambiguous apomorphies, notably including: quadrate with a foramen pneumaticum rostromediale (Character 52); humerus with the insertion of the principle part of the tendon of M. supracoracoideus an elongate scar distal to the tuberculum (Character 126); and Character 3, CI = 0.667, 0 ==> 2 (premaxilla dorsoventrally deep and narrow); and tibiotarsus with the distal opening of canalis extensorius aligned transversely to the axis (Character 248). Its poor resolution under parsimony analysis likely results from the considerable missing data for this taxon due mainly to the marked reduction in its pectoral girdle. It is however, important that *D*. *planei* showed no attraction to the large palaeognaths which are convergent in reduced pectoral girdle elements. Additionally, these analyses provide no support for the current consensus that dromornithids are anseriforms forming the sister group to either anhimids or anseranatids [23–24], nor to the more recent view that they were stem galloanseres [88]. However, to resolve this question adequately requires the inclusion of more dromornithid taxa and other putative relatives, which was beyond the scope of this paper, but is the subject of ongoing work.

Mayr [88] included *S*. *neocaledoniae* and *D*. *planei* whilst examining the phylogenetic relationships of Pelagornithidae and found these taxa to be weakly supported stem Galloanseres, although neither a monophyletic crown Galloanseres nor Anseriformes had significant support. Mayr’s [88] results concerning these taxa, while not the object of his study, are nevertheless compatible with our finding of Sylviornithidae and Dromornithidae as successive sister taxa on the galliform stem.

### Insights into relationships of fossil anseriforms

The inclusion of selected fossil anseriforms in the phylogenetic analysis has resulted in new insights into the relationships of key taxa, especially *Vegavis iaai* and the presbyornithid *Presbyornis pervetus*. The recovery of a topology for extant anseriforms concordant with current phylogenetic understanding, even when no molecular constraints were used (Fig 14), suggests confidence in the placement of the fossils within this clade. Both fossil taxa were recovered as anseriforms in a clade with Anatidae and *Anseranas semipalmata* (Anseranatidae) with moderate support. There was weak support for *P*. *pervetus* forming a clade with *A*. *semipalmata*, but this support was markedly enhanced when *V*. *iaai* was deleted from the analyses, doubtless due to significant missing data for *V*. *iaai*: only 32 of 285 characters were scored. This putative sister group relationship between *A*. *semipalmata* and *P*. *pervetus* is novel and counter to the current understanding of *P*. *pervetus* as the sister taxon to Anatidae [48, 77, 89].

This position for *Presbyornis* has implications concerning two significant characters. The presence of tela interdigitalis or interdigital webbing has long been said to characterise anseriforms, although this is rudimentary in anhimids [89]. *Presbyornis pervetus* had fully webbed feet [59], and if it is the sister group of *Anseranas semipalmata*, the semipalmate feet of the latter taxon would be best interpreted as a partial loss of the ancestral fully-webbed state. Livezey ([89]: 384) considered the four-notched sternum of *Presbyornis pervetus* to be a “unique bifurcation of the single, broad trabecula characteristic of other basal Anseriformes”, whereas our topology suggests that it could be an apomorphy for galloanseres secondarily lost in anseriforms except presbyornithids.

We also considered the relationships of *Anatalavis oxfordi*, which has either been considered to be a stem representative of Anseranatidae [33, 90] or the sister taxon of the clade uniting *Vegavis iaai*, Presbyornithidae, and Anatidae [60, 91]. Our initial analyses strongly supported a crown-anseriform relationship for *A*. *oxfordi* in a clade uniting *V*. *iaai*, Presbyornithidae, Anseranatidae and Anatidae, but its extensive missing data, as for *V*. *iaai*, prohibited further resolution. It was therefore deleted from further analyses, and further specimens, particularly ones preserving the unknown pelvis and legs are needed to better resolve its affinities.

*Vegavis iaai*, which was first described as a presbyornithid by Noriega and Tambussi [61], derives from 66.5 Ma rocks in Vega Island, Antarctica. Clarke et al. [60] used a stepwise phylogenetic analysis to conclude that the taxon was an anseriform that formed an unresolved trichotomy with Anatidae and *Presbyornis*, with successive outgroup taxa *Anatalavis*, *Anseranas* and anhimids. This relationship was based on scoring 11 characters into Livezey’s [89, 92] matrix of 123 plus 15 character matrix, and deleting all non-galloanserine taxa (see Supplementary Information, Clarke et al. [60]). We note that most of these 11 characters had plesiomorphic states, i.e. the state for *Vegavis* was shared with tinamous (7 characters) and/or more basal galloanseres (9 characters) as well as extensive homoplasy with non-galloanserine taxa, e.g., the hypotarsus character (90) state was shared with all the excluded non-galliform taxa. Regardless of these limited data, the placement of *Vegavis* as the sister taxon to Anatidae forming a clade that is the sister group to *Anseranas* has been and continues to be advocated as constraining the divergence of Anatidae from Anseranatidae to before 66.5 Ma and thus to be the most basal dated divergence event in crown group Aves [44, 60, 77, 93]. This position has been used in multiple phylogenetic analyses of Aves since Clarke et al.’s [60] analysis (e.g., [57, 58, 65, 94–97]). Our results cast doubt on the putative sister group relation of presbyornithids and anatids, and suggest that *V*. *iaai* forms an unresolved clade with anseranatids, presbyornithids and anatids. This is less resolved than the unresolved trichotomy of *Vegavis iaai* + *Presbyornis* + Anatidae as the sister group to *A*. *semipalmata* found by Clarke et al. [60]. Neither the clade Presbyornithidae nor *V*. *iaai* are supported as being more closely related to Anatidae than to Anseranatidae, contra recent authors [48, 60, 77, 89]. Until more data and a better resolved placement for *Vegavis* is possible, it only seems justifiable to use the 66.5 Ma age of this taxon to calibrate the divergence between anhimids and remaining anseriforms and not any more deeply nested divergence. Thus neither the clade Presbyornithidae nor *V*. *iaai* should be united with Anatidae in the Superfamily Anatoidea (Leach, 1819) [98] formed by Livezey [89], contra Ksepka and Clarke [77].

Our new position for *Presbyornis pervetus* in the anseriform phylogeny has important ramifications for fossil calibrations used in a majority of molecular phylogenetic analyses as *P*. *pervetus* derives from the Fossil Butte Member of the Green River Formation, with a reported ^40^Ar / ^39^Ar age of 51.66±0.17 Ma [99]. Older presbyornithids are known, such as *Teviornis gobiensis* Kurochkin et al., 2002 [100] from the Late Cretaceous (69–70 Ma) Nemegt Formation of Mongolia ([100], but see Ksepka and Clarke [77]) and less controversially the Mongolian Paleocene-Eocene boundary c. 55 Ma [101]. If presbyornithids are the sister taxon of Anseranatidae then their divergence should have a minimum calibration of 69 Ma, but more realistically, given the unresolved nature of the *Vegavis*/presbyornithid/anseranatid/anatid clade, this date should be applied to split between anhimids and remaining anseriforms, and thus is highly consistent with the more conservative interpretation of *V*. *iaai* discussed above.

Lastly, our phylogenetic analyses provide strong support for lithornithids being the sister group of Tinamidae, as obtained by Worthy and Scofield [39], Bertelli et al. [87], and Mitchell et al. [40], but this is only retrieved when tinamou and moa (*Dinornis robustus*) are constrained as sister-group taxa (conforming to robust molecular data, (e.g., [40, 64]). If tinamous and moa are not constrained together, then lithornithids pair with ratites as found by Houde [28]. Multiple apomorphies support the tinamou-lithornithid pairing, but most notable is the uniquely shared open fronto-parietal suture in adults (Character 15). This suture is not open in adult species of *Apteryx*, contra [49], but see [102], although fusion is slow in them as skeletal maturity takes several years [103, 104].

### Summary

Our new anatomical information and phylogenetic analysis finds that the large flightless bird *Sylviornis neocaledoniae*, originally described as a ratite, but then long regarded as a megapode, to be a stem galliform that forms a clade with *Megavitiornis altirostris*, forming Sylviornithidae. Neither species are megapodes, but the former perception that they were has led to the suggestion that *S*. *neocaledoniae* constructed the large mounds or tumuli in New Caledonia. Ectothermic incubation uniquely characterises Megapodiidae [21], with all included taxa depositing their eggs either in mounds, where heat from composting vegetation warms the eggs, or in holes dug in thermally heated soil or sand where sun can heat it such as in beach dunes. Our finding that it is a stem galliform (and thus not a megapode) makes it most unlikely to have been a mound-builder, as this scenario would require both mound-building and ectothermic incubation to have evolved twice. Furthermore, *Sylviornis neocaledoniae* shows no specific adaptation for digging to facilitate mound-building, unlike all extant megapodiids, making it even more unlikely that it exhibited ectothermic incubation.

## Supporting Information

S1 File Excel spreadsheet of measurements of megapodes and selected galliforms. (XLSX)

S2 File Word document of list of characters used in the Phylogenetic analysis. (DOCX)

S3 File The nexus file for the phylogenetic analysis conducted. (NEX)

S4 File Additional plots from the PCA. (DOCX)

## Abbreviations

AM: Australian Museum, Sydney, New South Wales, Australia
AMNH: American, Museum of Natural History, New York, U.S.A.
ANWC: Australian National Wildlife Collection, Canberra, Australia
IANCP: Institut d'Archéologie de la Nouvelle-Calédonie et du Pacifique, Nouméa, New Caledonia
KU: Ornithology Collection, University of Kansas Biodiversity Institute, Lawrence, Kansas, U.S.A
LACM: Vertebrate Zoology, Natural History Museum of Los Angeles County, Los Angeles, California, U.S.A.
NHMUK: Natural History Museum, London, United Kingdom
NMNZ: Museum of New Zealand Te Papa Tongarewa, Wellington, New Zealand
NMV: National Museum of Victoria, Melbourne, Victoria, Australia
QM: Queensland Museum, Brisbane, Queensland, Australia
SAM: South Australia Museum, Adelaide, South Australia, Australia
USNM: United States National Museum, Smithsonian Institution, Washington D.C., USA

ant: anterior
cmc: carpometacarpus
cor: coracoid
cran pt: cranial part
hum: humerus
fem: femur
fib: fibula
fur: furcula
juv: juvenile
mand: mandible
pt: part
rad: radius
scap: scapula
stern: sternum
tib: tibiotarsus
tmt: tarsometatarsus
L: left
R: right

DW: maximum distal width
PW: maximum proximal width
SW: mid-shaft width
TL: total length
