# Supplementary material for: Osteology Supports a Stem-Galliform Affinity for the Giant Extinct Flightless Bird Sylviornis neocaledoniae (Sylviornithidae, Galloanseres)
Source: PLoS One. 2016 Mar 30;11(3):e0150871. doi: 10.1371/journal.pone.0150871 (PMC4814122; doi:10.1371/journal.pone.0150871)
Supplement: S2 File — (DOCX) [file pone.0150871.s002.docx]

**Master Characters for galloanseres**

**Appendix 1.** Osteological characters used in the analysis. Characters marked with a star were treated as ordered in the analysis.

**TYPESET *Morphoclines = ord: 1 2 6 7 8 12-14 18 28 29 32 49 64 70 78 80 81 95 98 106 112 116 122 130 131 140 156 160 161 172 176 177 180 182 200 202 217 227-231 236 240 243 249 257 260-263 269 272 273 278-280 283 285;**

**unord: 3-5 9-11 15-17 19-27 30 31 33-48 50-63 65-69 71-77 79 82-94 96-97 99-105 107-111 113-115 117-121 123-129 132-139 141-155 157-159 162-171 173-175 178 179 181 183-199 201 203-216 218-226 232-235 237-239 241 242 244-248 250-256 258 259 264-268 270 271 274-277 281 282 284;**

**Skull**

*1. Premaxilla, length relative to cranium: 0, longer than cranium; 1, equal to cranial length; 2, shorter than cranium length. Cranial length in *Macrocephalon* did not include the casque dorsally but rather was from the caudal parts of the cranium.

*2. Premaxilla, length facies apertura nasalis compared to width craniofacial hinge, which in palaeognaths is interpreted as the rostral side of lacrymal: 0, long, greater than width of craniofacial hinge; 1, intermediate, approximately equal to width craniofacial hinge; 2, short, much less than width craniofacial hinge. See Worthy and Lee (2008, char. 3), not Livezey (1996a, char. 3), which is related to highly autapomorphic taxa.

3. Premaxilla: 0, sides divergent caudally; 1, spatulate; 2, not spatulate, dorsoventrally deep, with anteriorly steep culmen, nares reduced, e.g. *Sylviornis*.

4. Premaxilla, anterior section curved ventrally so ventral profile curved down: 0, Yes, (e.g. *Anseranas*); 1, No, straight ventral margin. Note, this is not the character of Livezey (1986: char. 12; 1996a: char. 10) & Worthy and Lee (2008, char. 16), which referred to the anterior tip of the median terminus of the premaxilla, which in e.g. *Malacorhynchus* is depressed ventrally.

5. Premaxilla, with culmen forming a distinct broad flattened ridge extending to tip defined by grooves laterally, with parallel structure ventrally on mandible: 0, yes; 1, no. From Parkes and Clark (1966), overlaps Ericson (1997, char set A: 21), (Worthy and Scofield 2012, char. 57) with state 0 a synapomorphy of palaeognaths.

*6. Upper beak, lamellae for filter feeding: 0, absent; 1, vestigial, 2, well developed. See Olson and Feduccia (1980, fig. 6) concerning the presence of vestigial lamellae in the Anhimidae. Mayr and Clarke (2003, char. 3).

*7. Premaxilla, palatal surface, fusion of proc. maxillopalatini: 0, not fused medially, fenestra ventromedialis open caudally (e.g. galliforms); 1, fused medially but incompletely, i.e., palate directly desmognathous, caudally enclosing elongate fenestra ventromedialis (e.g. *Anseranas,* anatids); 2, fused medially and completely and entire palate lacking fenestrae. Modified from Mayr and Clarke (2003: Fig. 5B, char. 11) and Worthy and Lee (2008, char 18), relates to Ksepka (2009, char. 9). Dinornithiforms have the processus maxillopalatini connected by the intervening vomer in perfect specimens so enclosing a fenestra ventromedialis but as the processus themselves are not fused medially are here coded 0.

*8. Skull, zona flexoria craniofacialis: 0, zona absent or indistinct, no transverse sulcus, overlap of processus frontalis of premaxilla and frontals and os nasales continuous over zona, e.g. palaeognaths; 1, well developed zona present marked by transverse sulcus, nasals and processus frontalis cross zona, e.g. *Anseranas*; 2, zona flexoria essentially a hinge, e.g. *Sylviornis*, *Dromornis*.

9. Cranium, os prefrontale (lacrimal), synostosis with os frontale and or os nasale: 0, lacking; 1, present but restricted to area caudal of craniofacial hinge; 2, present adjacent to nasal rostral to craniofacial hinge, and synostosed caudal to hinge. See Livezey (1986, char. 10; 1996a, char. 6), Ericson (1997, char set A: 5), Worthy and Lee (2008, char. 4). Ericson (1997) noted that incompletely ossified lacrimals typify Anhimidae, Anseranatidae, all Galliforms, Presbyornithidae, whilst in Anatidae they are fused. Bourdon (2011, char. 60) noted the condition was variable in anseriforms and inexplicably coded the character ‘inapplicable’ rather than variable. Here we noted that, in megapodes *Leipoa* and *Alectura* the lacrimal is fused and relatively vestigial, and it is even further reduced in *Megapodius*, but is large and unfused in *Gallus*. In dromornithids and *Macrocephalon*, in contrast to most birds, the lacrimal is fused to the nasal anterior to the zona flexoria (state 2). In *Sylviornis*, the lacrimal articulates via a small facet with the side of the nasal rostral to the craniofacial hinge, with complete synostosis caudally.

10. Cranium, lacrimal, presence proc. supraorbitalis, enclosing notch caudally: 0, process absent or small, variably laterally oriented; 1, large, encloses marked notch. Modified from Livezey (1986, char. 11 in part; 1996a, char. 7); Mayr and Clarke (2003, char. 13), Worthy and Lee (2008, char 5), Ksepka (2009, char. 6). Taxa with unfused or vestigial os prefrontales are coded 0. *Cereopsis* is coded 1 as the lateral margins of the lacrimal protrude markedly laterad and enclose a foramen caudally, even though caudally they are linked to osseous struts created from the autapomorphically deep salt gland impressions.

11. Cranium, os frontale, facies dorsalis, sulcus glandulae nasalis (salt gland impressions): 0, absent; 1, present, typically lateral. Is Livezey (1996a, char. 9 part), Mayr and Clarke (2003, char. 25), Worthy and Lee (2008, char. 7). Anatids are variable, contra Mayr and Clarke (2003), e.g. *Cereopsis* (1), *Anser* (0).

*12. Cranium, lacrimal, processus orbitalis lacrimale: 0, absent, e.g. *Leipoa*; 1, dorsoventrally short, tapering markedly ventrally (e.g. *Anseranas*); 2, dorsoventrally elongate. Here the process is coded the same despite whether is narrow, e.g. *Aythya australis*, or broad e.g. *Malacorhynchus*. Modified from Worthy and Lee (2008, char 10). In *Dromornis*, the processus orbitalis lacrimale is incorporated into the upper bill as a distinct strut caudad of the descending bar of the os nasale and linking to the processus jugale of the maxilla.

*13. Cranium, lacrimal – os ectethmoidale (ectethmoid) complex: 0, Ectethmoid not ossified, or just an incipient ridge dorsally below the nasals; 1, Ectethmoid ossified and small, not fused to lacrimal; 2, Ectethmoid ossified and large, fused to or abuts lacrimal forming lacrimal-ectethmoid complex (e.g. *Malacorhynchus*). Worthy and Lee (2008, char 11), Ksepka (2009, char. 8). The os ectethmoidale complex is that which encloses the capsula nasalis osseae and thus state 2 overlaps Bourdon’s (2011, char. 61) in which a well-developed mesethmoidale was found to be an apomorphy of Odontoanserae.

*14. Cranium, facies interorbitalis, depression frontalis: 0, absent; 1, shallow, elongate groove, variably extending to area between lacrimals or through the interorbital area; 2, marked concavity extending from between lacrimals through interorbital area. Modified from Worthy and Lee (2008, char 9). *Anseranas* treated as missing data because of unique ornament.

15. Cranium, fronto-parietal suture: 0, open; 1, closed. Mayr and Clarke (2003, char. 32). *Lithornis* and tinamous, but not *Apteryx* (contra Mayr and Clarke 2003) are open.

16. Cranium, projection of os squamosum lateroventrally links with an outgrowth of the ala parasphenoidalis anteriorly enclosing the recessus tympanicus rostralis: 0, absent; 1, present. See Worthy et al. (1997: char. 124), Worthy and Lee (2008, char 17). *Cereopsis* is 1.

17. Cranium, processus zygomaticus: 0, present; 1, absent or obsolete e.g. *Anseranas*, *Cereopsis*, See Zusi and Livezey (2000). The zygomatic process is present in most birds and is small in galliforms. In *Dromornis planei*, the proc. zygomaticus is fused to proc. postorbitalis (Murray and Megirian 1998) is coded 0. Overlaps Mayr and Clarke (2003, char. 33).

*18. Cranium, proc. zygomaticus, aponeurosis zygomatica : 0, unossified, e.g. *Dromaius*; 1. ossified, and often extends towards or converges with proc. postorbitalis but separated at its base by the impressio musculi adductoris mandibulae externus pars coronoidea on the lateral cranium in adults, e.g. *Alectura*; 2, ossified, and merges with proc. postorbitalis over whole length, e.g. anhimids. See Zusi and Livezey (2000) and Ksepka (2009, char. 12 and 13) here combined. *Dromornis* is 2. Anseriforms are coded 0 as the proc. zygomaticus is absent.

19. Cranium, impressio musculi adductoris mandibulae externus pars coronoidea et pars articularis (see, Zusi and Livezey (2000), both present laterally on cranium, small: 0, yes, e.g. *Leipoa*; 1, no, only pars articularis present laterally on cranium with pars coronoidea restricted to medial surface of proc. postorbitalis, e.g. anseriforms. In those taxa with a crista adductoris mandibulae externus articularis (see Zusi and Livezey 2000), e.g. anhimids, *Dromornis*, the pars coronoidea forms a sulcus medially at the junction of the processus postorbitalis and aponeurosis zygomatica, so are coded 1.This character is essentially the same as Ericson (1997, char. set A: 4) and overlaps Bourdon (2011, char. 59).

20. Cranium, recessus tympanicus (=tympanic cavity), lateral flange of the ala parasphenoidalis or a lateral projection of the lamina parasphenoidalis, and caudal connections with the proc. paroccipitalis of os exoccipitale: 0, recessus tympanicus not bound ventrally by laterally projecting ala parasphenoidalis, e.g. *Dromaius*; 1, recessus tympanicus bound ventrally by laterally projecting ala parasphenoidalis which links caudally to the proc. paroccipitalis lateral to fossa parabasalis. Much modified from Worthy and Lee (2008, char. 12).

21. Cranium, lacrimal, facies articularis frontonasalis, length relative to anterocaudal orbit diameter: 0, shorter than or equal to; 1, longer than. Worthy and Lee (2008, char. 13). *Dromornis planei*, lacking a lacrimal is coded -.

22. Cranium, tuba auditiva communis, separation of ostia (Livezey and Zusi 2006: char. 126): 0, widely separated; 1, closely approach mid line of cranium opening into ostium pharyngeale or tuba auditiva communis. Mayr and Clarke (2003, char. 29), Worthy and Scofield (2012, char. 23). In *Anhima*, the tuba auditiva communis open anteriorly in a single broad foramen, so is coded as 1. *Dromornis planei* is 1 as opening is within width of the rostrum.

23. Cranium, caudal part os basisphenoidale, ostium canalis opthalmici externa (for tubae auditivae, eustachian tubes) completely ossified ventrally: 0, yes; 1, no. Modified from Mayr and Clarke (2003, char. 28), Worthy and Scofield (2012, char. 22). *Dromornis planei* is 0.

24. Cranium, basiparasphenoid plate (= lamina parasphenoidalis) inflated ventrally below the tuba auditiva communis and the ostium pharyngeale and associated foramina rami palatinus et sphenomaxillaris, rounded, and broad: 0, no; 1, yes. In part Mayr and Clarke (2003, char. 26) and Worthy and Lee (2008, char. 19). With the inflation defined as relative to the tuba auditiva communis, *Anhima* is markedly inflated although it has a flat ventral surface. *Dromornis*, *Anseranas*, *Alectura* are 0, and *Gallus* is 1.

25. Cranium, lamina parasphenoidalis, caudolateral corner, development of tuberculum basilare (mamillar tuberosities), proc. mediales parasphenoidales (Livezey and Zusi 2006): 0, obsolete; 1, large and prominent. From Parker (1895), Pycraft (1900: 172), Livezey & Zusi (2006: char. 123), Mayr and Clarke (2003: char. 30), Worthy and Scofield (2012: char 18). *Dromornis planei* is 1.

26. Cranium, os opisthoticum/prooticum, pila otica with pneumatic openings lateral or caudolateral to it: 0, no; 1, yes. From Mayr and Clarke (2003, char. 31). Anatids 0, *Dromornis planei* is 0, palaeognaths are 1. Foramina anterolateral to the pila are not considered in this character. *Coturnix* differs from other galliforms examined with a taller and more anteriorly located pila otica and an extra foramen caudolaterally.

27. Cranium, os basisphenoidale (os parasphenoidale), presence of proc. basipterygoidei: 0, yes; 1, no.

*28. Cranium, os basisphenoidale (os parasphenoidale), position of proc. basipterygoidei: 0, on anterolateral corner of basitemporal platform caudal to rostrum parasphenoidale; 1, anterior to basitemporal platform on caudal end of caudally broad rostrum parasphenoidale; 2, on sides of rostrum parasphenoidale anterior to caudal end of rostrum, usually narrowly separated, e.g. Anatidae. Worthy and Scofield (2012, char. 12). Note: *Anhima* is not vastly different to some anatids contra Ericson (1996, 1997), but rather is quite similar to *Cereopsis*, with *Anhima*, *Cereopsis*, *Anser* forming a grade towards more rostral positioning and narrowly separated processes. Weber (1993) distinguished the condition in galloanseres (here 1-2), as a rostropterygoid articulation suggesting that this was non-homologous with the basipterygoid articulation of other birds e.g. palaeognaths. However, in all taxa, including palaeognaths, the processus occur on the os parasphenoidale and the differing states would be achieved by moving the position of the articulation zone rostrally, so I recognise three homologous states of one character, contra Weber (1993).

*29. Cranium, basipterygoid process, facet for articulation with pterygoid pedicellate (stalked): 0, yes, and elongate; 1, yes, short, ie facet is well raised above facies; 2, no.

30. Cranium, occipital region, fontanelles: 0, absent; 1, present. See Livezey (1986, char. 9), in part Ericson (1997, char. set A: 1), Mayr and Clarke (2003, char. 27), and Worthy and Lee (2008, char. 8 and notes). Taxa where fontanelles variably present are coded 1. *Dromornis planei* is 0.

31. Cranium, ventral view, paroccipital notch, i.e. between proc. paroccipitalis and the mamillar tuberosity, location of foramen n. vagi (vagus foramen for IX^th^ and X^th^ nerves): 0, in the notch; 1, caudad or mesad of the notch. From Parker (1895) and Pycraft (1900); Worthy and Scofield (2012, char. 10). In *Dromornis planei*, the vagus foramen is posterior to the paroccipitals and dorsal to the mamillar tuberosities within a common sulcus for the carotid canal, so is coded 1.

*32. Cranium, ventral view, location of external aperture of carotid canal: 0, posterior to paroccipital notch; 1, in paroccipital notch, e.g. *Leipoa*; 2, anterior to paroccipital notch. From Parker (1895) and Pycraft (1900). Modified from Worthy and Scofield (2012, char. 11) by combining the two states where the canal opens anterior to the paroccipital notch and adding a third state for the dromornithid condition where it opens posterior to the paroccipital.

33. Cranium, ventral view, position of proc. jugale of os maxillare relative to processus maxillopalatini (= processus palatus maxillaris Livezey and Zusi 2006) of maxilla: 0, dorsal or on same plane; 1, ventral. From Ericson (1997: char. 11). State 1 in *Anseranas*, Presbyornithidae, Anatidae; state 0 in *Megapodius*.

34. Cranium, os palatinum, angulus caudolateralis: 0, absent or very small; 1, present and prominent. Restricted from Mayr and Clarke (2003, char. 16) to the caudolateral process, as they used the pars lateralis and coded anhimids as 1, and anatids and galliforms as 0, but *Anser* and *Anseranas* have a markedly caudolaterally prominent angulus, while anhimids do not: galliforms lack caudolateral development.

35. Cranium, ossa palatina, development of crista ventralis: 0, poorly developed or absent; 1, strongly developed ventrally. See Ericson (1997, char. set A: 9), Mayr and Clarke (2003, char. 15). Poorly developed in anseriforms and galliforms and absent in Tinamidae and Presbyornithidae (Ericson 1997).

36. Cranium, ossa palatina completely fused along midline caudally, with or without vomer: 0, no; 1, yes. See Ericson (1997, char. set A: 8), Mayr and Clarke (2003, char. 17). Note: this separates anseriforms (1) from galliforms and palaeognaths, e.g. *Leipoa* is not fused (0). In dinornithiforms the palatines are separated by and fused to the vomer, but not to each other.

37. Cranium, vomer, caudal ends not fused, more or less deeply cleft: 0, yes; 1, no. From Mayr and Clarke (2003, fig. 5C, char. 19), separates palaeognaths and galliforms from anseriforms and *Anhima*.

38. Cranium, vomer, caudal end sleeves over (wraps around) rostrum parasphenoidale: 0, yes; 1, no, may or may not fuse to processus rostralis of ossa palatina.

39. Cranium, vomer mediolaterally wide: 0, yes; 1, no. From Mayr and Clarke (2003, fig. 5A), char. 20), separates palaeognaths (0) from galloanseres (1).

40. Cranium, vomer forms a narrow and dorsoventrally high lamella on the midline: 0, no; 1, yes. From Mayr and Clarke (2003, char. 21), separates palaeognaths (0) from anseriforms (1).

41. Cranium, os palatinum and os pterygoideum fused: 0, yes; 1, no. From Mayr and Clarke (2003, char. 22) and Ksepka (2009, char. 10), separates palaeognaths from neognaths.

42. Cranium, os pterygoideum: presence of a fossa on the dorsal facies caudal to the basipterygoid process: 0, absent or weakly developed (*D. planei*, *Ilbandornis*); 1, strongly developed fossa (*D. stirtoni*).

43. Cranium, os pterygoideum, articulation with palatine: 0, a simple abutment (*Dromornis, Anhima*); 1, a ball and socket joint with 1 main condyle (*Alectura, Megapodius*); 2, a ball and socket joint with prominent additional dorsal condyle (*Anseranas*, anatids); 3, pterygoid fused to palatine.

44. Cranium, os pterygoideum, location of basipterygoid facet: 0, no facet; 1, facet separated from rostral end by c. 1/2 length facet (*Anhima*); 2, facet closer to rostral end than (1) and may abut palatine articulation (*Dromornis*); 3, facet close to the caudal end.

45. Cranium, os exoccipitale, processus paroccipitalis, strongly protruding caudoventrally, caudally convex: 0, no; 1, yes. From Bourdon (2011, char. 56).

46. Cranium, processus postorbitalis stout and projecting obliquely rostrally beneath orbita: 0, no; 1, yes. From Ksepka (2009, char. 11) and Bourdon (2011, char. 57).

**Quadrate (Os quadratum)**

47. Quadrate, proc. mandibularis in lateral view, profile above cotyla quadratojugalis: 0, subparallel to plane across ventral margins of condyli lateralis et medialis, meeting ascending ramus of proc. oticus at about right angles (e.g. *Malacorhynchus*); 1, ascends to meet ascending ramus of proc. oticus in wide angle (e.g. *Anas*). Worthy and Lee (2008, char. 20).

48. Quadrate, pars mandibularis, processus lateralis pars quadratojugalis, prominentia submeatica on caudomedial facies: 0, absent; 1, present. Present in anseriforms but absent in most neornithines including Presbyornithidae (Ericson 1997, char. set A: 17). *Ortalis* has a small inflation interpreted by of Elzanowski and Stidham (2010) as a prominentia submeatica, and I take the process in this location in anhimids to be homologous to the prominentia in other anseriforms, so code them 1.*Tinamou* and *Lithornis* share a caudally directed prominence which is the caudal quadrate condyle and not equivalent to the prominence.

*49. Quadrate, capitulum squamosum and capitulum oticum: 0, form single elongate dumbbell shaped head, capituli with articular facets indistinct or essentially abutting each other; 1, have distinct articular facets widely separated by incisura intercapitulum; 2, one articular surface forming an oval head. Note: Overlaps with Ericson (1997, char. set A: 16), Mayr and Clarke (2003, char. 34), and Worthy and Scofield (2012, char. 51). When ordered, we assume that state 2 derives from state 1 by merging of the well-defined articular surfaces.

50. Quadrate, proc. oticus, tuberculum subcapitulare (=eminentia articularis): 0, absent; 1, present. Mayr and Clarke (2003: char. 35), with terminology of Elzanowski and Stidham (2010). While said to typify galloanseres (Elzanowski and Stidham 2010) the tuberculum is reduced to absent in some individuals. In *Anseranas*, there is no sign of the tuberculum: the feature labelled ‘proximal expansion of the subcapitular tubercle’ by Elzanowski and Stidham (2010) is part of the articular facet of capitulum oticum.

51. Quadrate, pneumatic foramen caudal to crista medialis on proc. oticus, foramen pneumaticum caudomediale, sensu Elzanowski and Stidham (2010): 0, no; 1, yes. Note: this character solely concerns presence or absence of a distinct foramen on the shaft, not whether there are foramina adjacent to the head. In *Anhima*, lateromedial compression of the quadrate results in this fossa lying on the medial facies, but as it is caudal to the crista medialis descending from the capitulum oticum, it is homologous with state (1). However, in *Cereopsis*, the only foramen is on the medial facies, which in the absence of a crista medialis, is interpreted as the foramen pneumaticum rostromediale and that the caudomedial foramen is absent, unlike other Anseriformes. Overlaps Mayr and Clarke (2003, char. 36), Worthy and Scofield (2012, char. 52).

52. Quadrate, pneumatic foramen on anteromesial surface of proc. oticus, equals foramen pneumaticum rostromediale (Elzanowski and Stidham 2010), rostral to the crista medialis: 0, no; 1, yes. Worthy and Scofield (2012, char. 50). Anseriforms are state 0, except for *Cereopsis*, see notes under character 51.

53. Quadrate, crista orbitalis proc. orbitalis (Elzanowski and Stidham 2010): 0, crista orbitalis weak and on bulge laterally on processus orbitalis; 1, crista orbitalis prominent and on bulge laterally on processus orbitalis; 2, crista mainly on tip of processus orbitalis, which has no lateral bulge. Elzanowski and Stidham (2010) list states 0 and 1 as an apomorphy of galloanseres. However, it is not angular in galliforms, more just a bulge, so the angular crest like expression seen in anseriforms is distinguished here.

54. Quadrate, foramen pneumaticum basiorbitale (see Elzanowski and Stidham 2010) ventrally on medial surface of processus oticus: 0, no; 1, yes. Elzanowski and Stidham (2010) list this as consistently present in galliforms (except *Phasianus*). Note: if the arcus pneumaticus separating the foramen pneumaticum basiorbitale from the foramen pneumaticum rostromediale is unossified as sometimes in *Leipoa* (see Elzanowski and Stidham 2010), then the state is coded (1). Contra Elzanowski and Stidham (2010) a small basiorbital foramen was observed in *Gallus*.

55. Quadrate, proc. oticus, capitulum oticum, pneumatic under capitulum: 0, no; 1, yes. Worthy and Scofield (2012, char. 53).

56. Quadrate, pterygoid articulations, condylus pterygoideus and facies artic. pterygoidea of the orbital process: 0, adjacent or fused to each other; 1, widely separated, distinct facet on orbital process. Elzanowski and Stidham (2010) argued on the basis that the articulations are adjacent in the majority of Neoaves, including *Anseranas*, anhimids, anatids and cracids, that the adjacent condition is likely the plesiomorphic condition. Widely separated articulations characterize *Presbyornis*, megapodiids, all phasianoid families.

57. Quadrate, the capitulum squamosum overhangs the lateral surface of the processus oticus: 0, yes (e.g. *Anseranas*); 1, no. Worthy and Lee (2008, char. 21).

58. Quadrate, the dorsolateral margin extending from processus oticus to the dorsal tip of the crista orbitalis: 0, forms straight line (e.g. *Dendrocygna*) or slightly concave; 1, markedly concave. This is Livezey (1986: char. 15), and see Raikow (1971), Worthy and Lee (2008, char. 22). For non galloanseres, a line from proc. oticus to tip of the orbital process is used, as this is where the ligamental attachment is.

59. Quadrate, pars quadratojugalis, fovea quadratojugalis, incisura caudalis (see Elzanowski and Stidham 2010): 0, absent; 1, present. A distinct caudoventral notch in the rim of the fovea is present in the anatids, most non-megapode galliforms (except Odontophoridae), and *Anhima*. The rim is complete in *Anseranas*, *Presbyornis* and most neornithines. We found the rim to be complete, albeit thin caudally, in *Leipoa*, contra Elzanowski and Stidham (2010) who reported it was half open in this genus and *Alectura*. *Megavitiornis* MNZ S37424 has an incisura, S37455 does not.

**Mandible**

60. Mandible – cotylae fossae articularis: 0, three cotylae, with cotyla medialis and cotyla lateralis separated by a shallow groove (sulcus intercotylaris), the crista intercotylaris restricted to the anterior part of the articulation area, caudal cotyla either separated from or merged with lateral cotyla; 1, two cotylae, cotyla medialis and cotyla lateralis large and separated by an anteroposteriorly oriented crista intercotylaris. Ericson (1997, char. set A: 18), overlaps Mayr and Clarke (2003: char. 38). This character is intrinsically related to the alignment of the quadrate condyles which feature is therefore not distinguished here as a separate character. In galloanseres, the long axis of the condylus medialis is parallel to that of condylus lateralis and the two condyli slightly overlap; in Neoaves the long axis of condylus medialis is roughly at right angles to the long axis of condylus lateralis.

61. Mandible, convex ventrally: 0, essentially lacking, < ½ depth os dentale (dentary) extends below middle of line linking the mandible tip and the cranial end of the dentary: 1, pronounced, > ½ depth below such line. This is Livezey (1996a: char. 4), Worthy and Lee (2008, char. 23). Mandibles of *Anhima* and megapodes are concave ventrally so are (0).

62. Mandible, deep groove in the ventral surface of the anterior portion of the mandibular rami: 0, absent; 1, present. Ericson (1997, char. set A: 20) and Ksepka (2009, char. 21).

63. Mandible, depth regio coronoidei: 0, shallower or slightly deeper than posterior end of dentary; 1, markedly deeper ( >1.5x depth) than that of the posterior dentary; 2, markedly reduced, depth much less than depth at angulus mandibulae at posterior end dentary, associated with rostral shift in ligamental insertions, e.g., *Dromornis*. Modified from Livezey (1996a: char. 5), Worthy and Lee (2008, char. 24), Ksepka (2009, char. 18).

*64. Mandible, proc. retroarticularis (retroarticular process), presence: 0, absent; 1, present, short, projection no more than depth at origin; 2, present, long, total projection > but <2x depth at origin adjacent cotylae; 3, present, very long, length greater than 2x depth at origin.

65. Mandible, impressio musculi depressor mandibulae, recessus conicalis: 0, absent; 1, present, shallow (e.g. *Anseranas*) or deep. Worthy and Lee (2008, char. 26). Note: the recessus is absent in galliforms and *Anhima*.

66. Mandible, proc. medialis mandibulae, foramen pneumaticum articulare: 0, present (e.g. *Anseranas*); 1, absent. Worthy and Lee (2008, char 27).

67. Mandible, splenial fused to dentary in adults: 0, yes; 1, no; weakly fused, only in part usually dorsal margin. Unfused condition typifies palaeognaths (Houde 1988), but we found megapodes to be unfused state and other galliforms to have a weakly fused condition.

68. Mandible, processus medialis mandibulae, long, narrow, and dorsally oriented: 0, no; 1, yes. A long, narrow, and dorsally oriented processus was listed as a synapomorphy of Galloanseres by Cracraft and Clarke (2001, char. 41), but Mayr and Clarke (2003, char. 45) found it more widely distributed, although it distinguishes palaeognaths from galloanseres.

69. Mandibular rami, lateral compression defining long, very narrow inter-ramal region: 0, absent; 1, present. See Livezey (1996b: char. 72), Worthy and Lee (2008, char. 128).

**Vertebrae**

*70. Vertebrae – total number of cervical and thoracic vertebrae: 0, 20 or fewer; 1, 21-22; 2, 23-25; 3, 26-30. Modified from Mayr and Clarke (2003, char. 55) and see Worthy and Lee (2008, char. 124) for data sources.

71. Thoracic vertebrae, pleurocoelous (with deep lateral depressions in the corpus) with or without pneumatisation: 0, no, lacks deep lateral fossae and not pneumatic; 1, yes, deep lateral fossae but not pneumatic; 2, corpus with large pneumatic foramen, but walls not compressed into fossa. From Ericson (1997, char. set A: 23) and Ksepka (2009, char. 24). Pleurocoelous vertebrae occur in Presbyornithidae, see Mayr and Clarke (2003, char. 58), but in Anhimidae the walls are highly pneumatic rather than compressed into a fossa and thus more simular to lithornithids and so a third state is recognised here.

72. Atlas, foramina transversaria: 0, absent; 1, present. Mayr and Clarke (2003, char. 47). *Dromaius* variably a foramen or well developed notch so coded 1: see Worthy and Scofield (2012: char. 66).

73. Axis, foramina transversaria: 0, present; 1, absent. Mayr and Clarke (2003, char. 49, fig. 6A) and Ksepka (2009, char. 22).

74. Axis, processus costales: 0, present; 1, absent. Mayr and Clarke (2003, char. 50, fig. 6B).

75. Axis, processus ventralis, size and shape: 0, low, not deeper than facies articularis of centrum; 1, present, pronounced, >depth facies articularis of centrum.

76. Third cervical vertebra, osseous bridge from processus transversus to processus articularis caudalis enclosing a foramen: 0, absent; 1, present. From Mayr and Clarke (2003, char. 52) who code galloanseres as invariant, with Anhimidae, Anatidae, and Galliforms all 1 - but in *Anhima* MV B12574, the foramen is open; see Ksepka (2009, char. 23).

77. Posterior caudal vertebrae with well-developed processus haemales ventrally, projects anteriorly off pygostyle: 0, no; 1, yes. From Mayr and Clarke (2003, char. 59, fig. 6H), where galliforms = 0 and Anatidae and Anhimidae = 1.

*78. Thoracic vertebrae fused/co-ossified forming a notarium: 0, no; 1, yes two vertebrae fuse, e.g., *Anseranas*; 2, yes, three or four vertebrae fuse forming notarium. Modified from Worthy and Lee (2008, char. 127) and Ksepka (2009, char. 25).

**Sternum**

79. Sternum, corpus sterni, presence of pneumatic foramina on sulcus medianus sterni (midline): 0, yes, multiple, no single large one, and extending more caudally (e.g. *Anseranas*); 1, single well-defined foramen anteriorly; 2, no foramen; 3, large well-defined foramen on midline centred on costal processes, may have smaller foramina associated with it, e.g. *Gallus*. Modified from Livezey (1986, char. 78; 1996a, char. 24) and Worthy and Lee (2008, char. 29).

*80. Sternum, corpus sterni, pars cardiaca, pori pneumatici: 0, widely scattered on it (e.g. *Anseranas*); 1, limited to caudal margin of pila coracoidea (e.g. *Cereopsis*); 2, essentially absent. From Livezey (1986, char. 89; 1996a, char. 23); Worthy and Lee (2008, char. 30).

*81. Sternum, corpus sterni, margo costalis, number of proc. costalis: 0, seven or eight; 1, five or six, e.g. *Anseranas*, *Cereopsis*; 2, three or four, e.g. galliforms. See Livezey (1996a: char. 25), Mayr and Clarke (2003, char. 71), and Worthy and Lee (2008, char. 31). *Anseranas* SAM B36790 has 6 processus, but both sides having a vestigial processus anterior of the first, which is not included in the total.

82. Sternum, corpus sterni, margo caudalis with thickened ridge: 0, present; 1, absent (e.g. *Cereopsis*, coincident with carina sterni not extending to caudal margin). Modified from Livezey (1996a, char. 26) and Worthy and Lee (2008, char. 32).

83. Sternum, rostrum sterni, spina interna rostri (dorsal manubrial spine): 0, absent, or a broad shallow medial notch bound by prominences on the labrum internum, includes situation where a broad notch has a medial prominence (e.g. *Anas*); 1, present, either a rectanguloid flange (e.g. *Malacorhynchus*) or triangular protuberance, e.g. galliforms. Modified from Livezey (1986: char. 82; 1996a, char. 28), Worthy and Lee (2008, char. 33), Ksepka (2009, char. 33).

84. Sternum, rostrum sterni, pila coracoidea: 0, thickened, robust ridge; 1, thin, not at all thickened (e.g. *Oxyura*). Worthy and Lee (2008, char. 34).

85. Sternum, rostrum sterni, spina externa rostri (ventral manubrial spine): 0, lacking (e.g. *Dendrocygna*); 1, present, robust, short to long, bifid or pointed, but not connecting to spina interna; 2, present and bladelike, connecting to spina interna. Mayr and Clarke (2003, char. 70), Worthy and Lee (2008, char. 35), Ksepka (2009, char. 34).

86. Sternum, margo costalis, location of distal-most costal process: 0, in distal half of corpus sterni (basin) measured from dorsal lip coracoidal sulcus and costal length >1/2 basin length (e.g. *Anseranas*); 1, in distal half of basin but costal length <1/2 basin length (e.g. *Dendrocygna*); 2, at or anterior of midpoint of basin length, costal length <1/2 basin length. Modified from Livezey (1986, char. 86), Worthy and Lee (2008, char 37).

87. Sternum, caudal margin, number of notches or fenestrae: 0, four; 1, two; 2, none. Mayr and Clarke (2003, char. 73), Ksepka (2009, char. 40). *Dromornis* is interpreted as having 0 notches caudally though in some specimens there perhaps are a pair of very shallow notches but this is compromised by preservation.

**Scapula**

88. Scapula, scapus scapulae (blade), dorsoventral height: 0, uniform or decreases over first 2/3 of length; 1, increases distally of collum scapulae to a maximum at about ½ to ¾ blade length. Modified from Livezey (1986, char. 108; 1996a, char. 40); Worthy and Lee (2008, char. 38).

89. Scapula, acromion, cranial extent with collum scapulae horizontal: 0, equal to or caudal to tuberculum coracoideum (e.g. *Anseranas*); 1, extends distinctly craniad of tuberculum coracoideum. See Livezey (1986, char. 109; 1996a, char. 38); Ericson (1997, char. set A: 48); Worthy and Lee (2008, char. 39).

90. Scapula, cranial end, foramen pneumaticum: 0, present laterally, variable in shape; 1, absent. See Livezey (1986, char. 111; 1996a, char. 37), Worthy and Lee (2008, char. 40), Ksepka (2009, char. 46).

91. Scapula, ventral facies under facies articularis humeralis, pneumatic fossa present: 0, no; 1, yes; this obvious feature in megapodiids appears to have not been used in character analyses previously.

92. Scapula, acromion, attachment for ligamentum acrocoraco-procoracoideum: 0, absent or poorly developed crista, e.g. *Anseranas*; 1, well developed tuberculum projecting costally, e.g. *Megapodius*. The derived state (1) may relate to the much reduced processus procoracoideus in galliforms and transfer of the primary insertion of this ligament to the scapula from the coracoid. The derived state causes the ‘hooked’ state in Ksepka (2009, char. 43).

**Coracoideum**

93. Coracoid, presence of foramen/incisura nervi supracoracoidei: 0, yes, foramen opens into corpus; 1, yes, but not opening into corpus; 2, no foramen. Modified from Livezey (1986, char. 92; 1996a, char. 43), Ericson (1997: char. set A: 37), Mayr and Clarke (2003, char. 65), Livezey and Zusi (2006, char. 1286); Worthy and Lee (2008, char. 42), Ksepka (2009, char. 49). Galliforms are coded as lacking foramen even though they lack a procoracoid, because in tinamous and lithornithids the foramen enters the shaft and is not related to the procoracoid.

94. Coracoid, procoracoid, presence: 0, absent or present with minimal projection medially; 1, an obvious process extending medially of corpus. Ericson (1997, char. set A: 41) identified Tinamidae and Galliformes as lacking a processus. Tinamou coded as having a procoracoid that is short and robust with foramen lateral to it and which partly has scapula cotyla on it. Ksepka (2009, char. 48).

*95. Coracoid, omal end, facies articularis scapularis: 0, deep cotyla present, e.g. *Cereopsis*; 1, shallow cotyla, e.g. *Anseranas*; 2, forms a subplanar to convex subcondylar articulation. Ericson (1997, char. set A: 40); Livezey and Zusi (2006, char. 1281), Ksepka (2009, char. 47). This character complex is intrinsically linked to the presence or absence of a tuberculum coracoideum on the scapula, which therefore is not described as a separate character.

96. Coracoid, omal end, processus acrocoracoideus (acrocoracoid) with pneumatic foramina under facies artic. clavicularis (clavicle facet): 0, lacking; 1, present, in well-defined fossa below dorsal part of clavicle facet only; 2, present, in a broad area under clavicle facet (e.g., *Cereopsis*); 3, foramina present under ventral part of clavicle facet only. Modified from Livezey (1986, char. 95; 1996a, char. 42); Worthy and Lee (2008, char. 43).

97. Coracoid, omal end, ventral facies, processus acrocoracoideus, depth of sulcus medial to facies artic. humeralis and sternal to impressio ligamentum acrocoracohumeralis: 0, shallow groove, e.g. *Alectura*, *Anseranas*; 1, deep groove, e.g. *Gallus, Anhima*.

*98. Coracoid, omal end, processus acrocoracoideus, facies artic. clavicularis, dorsal and ventral lobes: 0, not projected over sulcus m. supracoracoidei; 1, not projected equally, only dorsal lobe overhangs sulcus; 2, both lobes slightly overhang supracoracoidal sulcus; 3, pronounced overhang of both lobes over supracoracoidal sulcus (e.g. *M. membranaceus*). Modified from Livezey (1986, char. 97), Worthy and Lee (2008, char. 44). For flightless taxa with reduced coracoids this character is coded as inapplicable.

99. Coracoid, omal end, processus acrocoracoideus, orientation relative to facies articularis humeralis in dorsal view: 0, acrocoracoid directed primarily cranially forming wide angle with humeral facet; 1, directed primarily medially forming near right angle with medial margin of humeral facet, e.g. galliforms.

100. Coracoid, omal end, facies articularis humeralis: 0, concave articular surface in dorsoventral plane, e.g., *Cereopsis*; 1, flat or convex, e.g., *Gallus*. This does not relate to the omal-sternal plane.

101. Coracoid, omal end, processus acrocoracoideus, projection of cranial end over medial margin of sulcus m. supracoracoidei in dorsal aspect: 0, little or none (e.g. *Anseranas*, *Tadorna*); 1, significantly projected (e.g. *Gallus*). In part Worthy and Lee (2008, char. 45).

102. Coracoid, omal end, sulcus m. supracoracoidei, excavated under facies artic. humeralis: 0, absent; 1, present. Worthy and Lee (2008, char. 46). For flightless taxa with reduced coracoids this character is coded as missing data.

103. Coracoid, omal end, sulcus m. supracoracoidei, corpus at ventromedial margin: 0, rounded, relatively thick, e.g. megapodes; 1, compressed, keeled, e.g., *Gallus*.

104. Coracoid, corpus, facies dorsalis, foramen pneumaticum in impressio m. sternocoracoidei: 0, present; 1, absent. After Livezey (1986, char. 93; 1996a, char. 44); Mayr and Clarke (2003, char. 67), Worthy and Lee (2008, char. 47), Ksepka (2009, char. 51).

105. Coracoid, corpus, facies dorsalis, with several striae of muscle scars diagonally traversing it: 0, no; 1, yes. Ericson (1997, char. set A: 38) found such striae typify Anatidae, Anseranatidae, and Presbyornithidae, and are absent in galliforms, however, diagonal striae are seen in threskiornithids.

*106. Coracoid, corpus, facies ventralis, a well-defined impressio m. supracoracoidei bound laterally by a linea muscularis and caudally by the facies artic. sternalis: 0, flat or convex ventrally, e.g., *Gallus*; 1, present, distinct but shallow (e.g. *Oxyura*, *Malacorhynchus*); 2, present deep, typified by *Stictonetta* and *Dendrocygna*. Modified from Livezey (1986, char. 96; 1996a, char. 45); Worthy and Lee (2008, char. 48).

107. Coracoid, ventral facies artic. sternalis: 0, ventral facet distinct with rounded cranial margin, may or may not be elevated with respect to adjacent facies, separated from dorsal facet by distinct crest (e.g. *Anseranas*); 1, ventral facet not prominent ventrally, directed sternally, continuous via rounded ridge to dorsal facet; 2, ventral facet absent or indistinct. Modified from Livezey (1986, char. 100), Worthy and Lee (2008, char. 49), Ksepka (2009, char. 52).

108. Coracoid, corpus, orientation, line linking proc. acrocoracoideus and angulus medialis forms angle with line linking lateral and medial extremes of sternal facet: 0, markedly greater than 90-100º; 1, approximates 90-100º. Worthy and Lee (2008, char. 50). For flightless taxa with reduced coracoids this character is coded as inapplicable.

109. Coracoid fused with scapula: 0, no; 1, yes. Mayr and Clarke (2003, char. 68).

**Furculum**

110. Furcula, processus acromialis: 0, broadly rounded; 1, pointed. Ericson (1997, char. set A: 45).

111. Furcula, claviculae, lateromedial curvature: 0, little; 1, significant curvature.

*112. Furcula, apophysis furculae (furcular process or hypocleideum) projecting dorsocaudally: 0, obsolete, no structure visible; 1, present as low ridge, either single (e.g. *Tadorna*) or paired (e.g. *Cereopsis*); 2, present as a prominent ridge or lobe, e.g., galliforms. Modified from Livezey (1986, char. 102; 1996a, char. 33), Ericson (1997, char. set A: 46), Worthy and Lee (2008, char. 121), and Ksepka (2009, char. 32).

113. Furcula, scapus claviculae, facies lateralis: 0, lacking foramina pneumatica; 1, with foramina pneumatica, e.g., *Cereopsis*. See Livezey (1986, char. 105; 1996a, char. 35), Worthy and Lee (2008, char. 122).

114. Furcula, overall form apophysis furculae: 0, robust, tending towards V-shaped with furculae divergent dorsally; 1, robust, U-shaped; 2, slender, broadly U-shaped; 3, slender, V-shaped. Modified from Ericson (1997, char. set A: 44), Worthy and Lee (2008, char. 123), and Ksepka (2009, char. 29).

**Humerus**

115. Humerus, margo caudalis, capital shaft ridge present and extends alongside and proximal to fossa pneumotricipitalis: 0, yes; 1, no. Worthy and Lee (2008, char. 51).

*116. Humerus, capital shaft ridge, when present and prominent: 0, directed towards caput (head) (e.g. *Anseranas*); 1, directed towards the zone between the head and the tuber. dorsale (dorsal tuberosity); 2, directed/extends to dorsal tuberosity. Modified from Livezey (1986: char. 22; 1996a: char. 51) and Worthy and Lee (2008, char. 52).

117. Humerus, margo caudalis, area adjacent and distal to end of crista deltopectoralis compressed into a ridge that does not extend further proximally: 0, yes, e.g. megapodes; 1, no, e.g., *Gallus*. This ridge lies more distad than the capital shaft ridge of characters 115 and 116. Anseriforms in which the capital shaft ridge extends distad of the crista bicipitalis lack the angularity of the ridge as seen in e.g. *Leipoa* and are coded 1.

118. Humerus, proximal end, fossa pneumotricipitalis dorsalis (dorsal or secondary pneumotricipital fossa) between incisura capitis (capital groove) and tuberculum dorsale: 0, absent (e.g. *Anseranas*, *Cereopsis*); 1, dorsal head of M. humerotriceps inserts in a shallow flat fossa extending to base of head whose width < fossa pneumotricipitalis ventralis; 2, wide, shallow fossa ≥ width ventral pneumotricipital fossa; 3, Dorsal pneumotricipital fossa deep. Modified from Livezey (1986, char. 23 & 24), Worthy and Lee (2008, char. 53), Ksepka (2009, char. 55).

119. Humerus, fossa pneumotricipitalis dorsalis (dorsal pneumotricipital fossa) excavated below caput humeri: 0, no; 1, yes. Worthy (2009, char. 134). Taxa lacking said fossa are non-comparable.

120. Humerus, relationship of incisura capitis humeri to facies at its dorsal end: 0, opens dorsally at same level; 1, groove is elevated above (more caudal) the facies; 2, separated from the caudal facies by a distinct crista incisurae capitis distalis that closes the groove. Modified from Ksepka (2009, char. 58) and Worthy (2009, char. 135).

121. Humerus, proximal end, crista deltopectoralis (deltoid crest): 0, caudally (anconally) concave, profile of crista rounded over length; 1, caudally flat or convex, profile angular. From Livezey (1986, char. 25), Ericson (1997, char. set A: 55) and Worthy and Lee (2008, char. 54). Note that anatids are polymorphic with more basal taxa having a caudally concave crista deltopectoralis (Worthy and Lee 2008). All species of *Oxyura* are here considered to have a flat deltoid crest (1) that is convergent with the condition in anatines.

*122. Humerus, proximal end, crista deltopectoralis, in cranial/palmar view, length relative to junction of crista bicipitalis (bicipital crest) with shaft: 0, about 50% of length of deltoid crest extends distad of bicipital crest; 1, about 30-40% of length of deltoid crest extends distad of bicipital crest; 2, significantly less than 30% of deltoid crest extends distad of bicipital crest. Worthy and Lee (2008, char. 55).

123. Humerus, proximal end, crista deltopectoralis, in cranial/palmar view, apex of crista markedly deflected ventrally to overhang cranial facies: 0, no; 1, yes, e.g., *Gallus*, but not megapodes.

124. Humerus, proximal end, tuberculum dorsale (dorsal tuberculum): 0, prominent, buttressed, elevated from surface of shaft; 1, essentially coplanar with shaft. Modified from Livezey (1986, char. 32) and Worthy and Lee (2008, char. 56).

125. Humerus, tuberculum dorsale shape: 0, width roughly equals length; 1, elongate ovate; 2, elongate, narrow lenticular scar merging with proximolateral facies of deltoid crest. Worthy (2009, char. 136). The scar in state 2 (galliforms) is proximal to the insertion of the principle part of the tendon of M. supracoracoideus. In State 2, the insertion area forms this elongate scar on a rounded elevated tuberosity in cracids, so coded 2. In anatids, the insertion area may be broad or long ie 0 or 1.

126. Humerus, proximal end, insertion of the principle part of the tendon of M. supracoracoideus: 0, restricted to the tuberculum dorsale; 1, forms elongate scar extending distal to tuberculum dorsale, e.g., galliforms. Reinterprets Mayr and Clarke (2003, char. 76) and overlaps Ksepka (2009, char. 57).

127. Humerus, proximal end, attachment site of M. scapulohumeralis cranialis, see Hiroshige and Yoshikazu (2007), location relative to crus dorsal fossae: 0, partly on or dorsally to crus; 1, ventral to crus. In *Gallus* and *Leipoa*, the insertion lies distal to the fossa pneumotricipitalis and ventral to the crus, whereas in *Anseranas* and *Cereopsis*, it extends dorsal to the crus. Replaces Worthy (2009, char. 138).

128. Humerus, proximal end, tuber. ventrale, in caudal view: 0, directed proximally so does not overhang the fossa pneumotricipitalis ventralis; 1, directed caudo-cranially so its distal margin either is directed at right angles to the fossorial plane or overhangs the fossa pneumotricipitalis ventralis. Modified from Livezey (1986, char. 27) and Worthy and Lee (2008, char. 57).

129. Humerus, proximal end, fossa pneumotricipitalis ventralis: 0, open, highly pneumatic, cavity with bony struts extends under margo caudalis (shallow in *Anseranas*, deep in e.g. *Tadorna*); 1, closed by bony wall internally, forming a conical-shaped pocket or fossa with the apex extending under the head and in caudal view the medial margin of this fossa forming a planar surface extending from the apex of the fossa to the junction of the bicipital crest and shaft. Modified from Livezey (1986, char. 28) and see Worthy and Lee (2008, char. 58) for notes on the distribution of states in this character related to diving.

*130. Humerus, proximal end, incisura capitis, either caudal or cranial view: 0, proximal profile not or barely interrupted by incisura; 1. proximal profile with very shallow notch, e.g., tadornines; 2, proximal profile with distinct notch created by incisura. Worthy and Lee (2008, char. 59).

*131. Humerus, proximal end, crista bicipitalis (bicipital crest), shape in caudal view: 0, width across the fossa pneumotricipitalis ventralis from crus dorsale fossae to crus ventrale fossae distinctly less than length from tuberculum ventralis to junction of crista bicipitalis and shaft (e.g. *Anseranas*); 1, width approximately equals length; 2, width distinctly greater than length e.g. *Anas*. Worthy and Lee (2008, char. 60). For *Cnemiornis* this character is coded as missing data as reduction of the humerus coincident with flightlessness precludes determining the homologous extent of the bicipital crest.

132. Humerus, insertion for M. coracobrachialis caudalis: 0, situated on tuberculum ventrale and separated from caput by incisura capitis; 1, forms distinct depressio insertii m. coracobrachialis caudalis (see Livezey and Zusi 2006, char. 1361) at dorsal side of incisura capitis indenting the crista incisurae capitis distalis. In most birds this insertion is on the tuberculum ventralis, but in galliforms it is situated at the dorsal side of the incisura capitis. See Ericson (1997, char. set A: 52, 53).

133. Humerus, insertion for M. coracobrachialis caudalis when at dorsal side of incisura capitis indenting the crista incisurae capitis distalis: 0, abuts a small distal projection (tuberculum intermedium) on the caput, e.g. megapodes; 1, is bound dorsally by marked tuberculum intermedium projecting from caput, e.g. *Gallus*; 2, no project from caput bounding it dorsally. Anseriforms are inapplicable.

134. Humerus, attachment of m. latissimus dorsi pars cranialis (=latissimus dorsi anterioris) is: 0, located dorsad of the margo caudalis, e.g. megapodes and anseriforms; 1, ventrad to the margo caudalis, e.g. all other galliforms (Mourer-Chauviré 1992).

135. Humerus, shaft with essentially parallel sides in caudal or cranial views: 0, yes; 1, no, narrows distally (at least 10% reduction on mid-length width), narrowest point in distal third; 2, no, narrowest near midshaft point, e.g. galliforms. Modified from Worthy and Lee (2008, char. 61).

136. Humerus, proximal end, attachment scar of m. latissimus dorsi pars cranialis (=anterioris see Howard 1929: fig 20) (as distinct from attachment for m. latissimus dorsi posterioris), location, caudal view: 0, adjacent to and proximally overlaps with distal end of deltoid crest; 1, markedly caudad of and distal to crista deltopectoralis, e.g. galliforms; 2, markedly caudad of and broadly overlaps in proximodistal plane crista deltopectoralis. Modified from Worthy and Lee (2008, char. 62).

137. Humerus, distal end, relative distal extent of proc. flexorius (= entepicondyle) to line drawn across distal extreme of condyli ventralis et dorsalis, in cranial view: 0, short, ends markedly proximad to condyli, e.g. *Anseranas*; 1, long, distal extent roughly equal to that of the condyli, e.g. tadornines and anatines. Modified from Worthy (2009, char. 63). This is another way of assessing distal extent of condylus ventralis Ksepka (2009, char. 59).

138. Humerus, distal end, tuber. supracondylare dorsale (ectepicondylar prominence), in cranial view: 0, present, a distinct caudo-cranially thickened prominence on dorsal margin at proximal end of the dorsal condyle (e.g. *Anseranas*); 1, No prominence distinct from epicondylus dorsalis, which usually forms a low short proximally directed ridge. Note: In most birds, the tuber. supracondylare dorsale supports the origin of M. extensor carpi radialis (see Baumel and Witmer 1993), but in galloanseres, the two scars for the origin of M. extensor carpi radialis lie immediately proximal to the tuberculum and are less prominent dorsally. The scar for the insertion of M. ectepicondyloulnaris lies on the dorsal facies of the tuber. supracondylare dorsale, but the presence of this scar is not correlated with tuberculum size in anseriforms. All galloanseres have a tuberculum very much smaller than in taxa such as charadriiforms and procellariiforms.

139. Humerus, distal end, tuber. supracondylare ventrale (attachment of the anterior artic. ligament): 0, attachment facet parallel to shaft, not buttressed proximally (e.g. *Anseranas*); 1, facet buttressed proximally, tilted distally and/or medially (e.g. *Anas*). Modified from Livezey (1986, char. 26) and Worthy and Lee (2008, char. 65). Note, the facet is medially rotated in diving waterfowl (Worthy and Lee 2008), so rotation is not factored into this character. Presence/absence of a buttress is considered the primary feature here, so for *Anser* in which the tubercle has little distal tilt but is clearly buttressed, is assigned state ‘1’.

*140. Humerus, distal end, sulcus scapulotricipitalis (external tricipital groove): 0, absent or barely defined (e.g. *Anseranas*); 1, present on caudal face, but not extending around distal end of epicondylus dorsalis (e.g. *Dendrocygna*); 2, present, extends distally around epicondylus dorsalis, forms distal notch in caudal view. In part, Mayr and Clarke (2003, char. 81), Worthy and Lee (2008, char. 66).

141. Humerus, distal end, attachment of pronator brevis, *sensu* (Howard 1929), origin of the proximal head of the M. pronator superficialis: 0, pit on ventral facies, separated from facet on tuber. supracondylare ventrale; 1, pit incorporated into ventral margin of facet on tuber. supracondylare ventrale. Worthy and Lee (2008, char. 67).

142. Humerus, proximal end, tuberculum ventrale, projection caudally, medial or proximal view: 0, projects more caudad than caput; 1, little projection about same as caput.

143. Humerus, distal end, ventral facies, scar for M. flexor carpi ulnaris on processus flexorius: 0, one large scar; 1, two scars of approximately equal depth; 2, two scars, caudal one shallow, cranial one deep. Ericson (1997, char. set A: 60) noted only a single scar in Tinamidae, Galliformes and a few other taxa; Anseriformes have a double one.

144. Humerus, width of space between the facet on the tuber. supracondylare ventrale and the proximoventral apex of the dorsal condyle: 0, narrow, gap equal to or narrower than width facet; 1, wide, gap wider than facet. Derived from Woolfenden (1961) and Worthy (2009, char. 137).

145. Humerus, fossa olecrani (olecranal fossa): 0, absent or shallow; 1, deep, well defined. Modified from Worthy (2009, char. 139) to incorporate galliforms.

**Ulna**

146. Ulna, proximal end, cranial (palmar) view, width: 0, dorsoventral width greater than breadth from olecranon to cranial margin of cotyla ventralis, e.g. *Cereopsis*; 1, dorsoventral width less than breadth from olecranon to cranial margin of cotyla ventralis, e.g. *Gallus*. This character captures the more significant of six features contributing to Bourdon’s (2011, char. 64) and distinguishes galliforms from anseriforms.

147. Ulna, proximal end, ventral facies, tuberculum lig. collateralis ventralis and insertion for trochlea humeroulnaris on side of olecranon: 0, tuberculum lig. collateralis ventralis large, ventrally convex, separated by a sulcus from insertion for trochlea humeroulnaris which is aligned craniocaudally across olecranon, e.g. *Tadorna*, *Anseranas*; 1, tuberculum lig. collateralis ventralis flat on ventral facies, with no sulcus between it and the insertion for trochlea humeroulnaris which is aligned proximodistally on the olecranon, e.g. *Leipoa*, *Gallus*; 2, tuberculum lig. collateralis ventralis flat on ventral facies, distinctly separated distally from the insertion for trochlea humeroulnaris which is aligned craniocaudally across olecranon..

148. Ulna, proximal end, cranial (palmar) view, tuber. bicipitale ulnae (for insertion of *M. biceps brachii*): 0, forms single prominent elongate tuberculum extending from just distal to the cotyla ventralis diagonally and distally towards a point distad of the processus cotylaris dorsalis, although two insertions may form abutting scars (e.g. *Anseranas*); 1, two separated insertions, one starts adjacent to cotyla ventralis and extending disto-ventrally to a level distad of the cotyla dorsalis; the second lies further distally, distal to the dorsal cotylar process (e.g. *Malacorhynchus*, *Anhima*); 2, two separated insertions: one starts adjacent to cotyla ventralis and extends across incisura radialis ending proximal to distal end of cotyla dorsalis; the second lies distal to cotyla dorsalis, e.g. *Anser*; 3, forms single scar extending from distal to cotyla ventralis to a ridge extending distoventrally from cotyla dorsalis enclosing a marked incisura radialis. Modified from Worthy and Lee (2008, char. 69). Tinamou has a short ridge extending from the ventral condyle towards a ridge projecting from dorsal cotyla, although they do not meet, but as this is like e.g. *Presbyornis*, although the ventral ridge is shorter, it is coded the same.

149. Ulna, proximal end, fossa or pneumatic foramen under processus cotylaris dorsalis: 0, absent; 1, present (e.g. *Malacorhynchus*). Worthy and Lee (2008, char. 70).

150. Ulna, length: 0, approximately equal to or greater than length humerus; 1, significantly (>5%) shorter than humerus. Modified from Mayr and Clarke (2003), Worthy and Lee (2008, char. 71) and Ksepka (2009, char. 60). Flightless taxa, or those with reduced flight ability, are scored as inapplicable.

151. Ulna, shaft compressed dorsoventrally with dorsoventral width markedly less than craniocaudal depth and ventral side flattened adjacent to the distal end of the brachial fossa: 0, yes; 1, no. From Ericson (1997, char. set A: 61) and Mayr and Clarke (2003, char. 83).

152. Ulna, distal end, condylus ventralis low, not prominent ventrally, with sulcus intercondylaris wide and shallow: 0, yes, e.g. megapodes; 1, no. Ericson (1997: char set A 62).

153. Ulna, distal end with marked depressio radialis: 0, no; 1, yes e.g., *Anseranas*. From Mayr and Clarke (2003, char. 84).

154. Ulna, distal end, tuberculum carpale, cranioventral projection: 0, tuberculum carpale large, projects more than half the craniocaudal width of the dorsal and ventral condyles, e.g. anseriforms; 1, tuberculum carpale small, projects less than half the craniocaudal width of the dorsal and ventral condyles, e.g. *Leipoa*, *Gallus*.

**Carpometacarpus**

155. Carpometacarpus, proximal end, dorsal and caudal aspects, external rim trochlea carpalis with marked notch caudally: 0, no, rim extensive with even convex profile caudally extending to fovea carpalis caudalis (e.g. *Anseranas*); 1, yes, rim extensive with marked notch caudally in rim that extends to fovea carpalis caudalis; 2, rim short, ends distally at position of notch in previous character state (e.g. galliforms). Modified from Livezey (1986, char. 37+38; 1996a, char. 55), Worthy and Lee (2008, char. 72), and from Ksepka (2009, char. 61).

*156. Carpometacarpus, proximal end, caudal view, distal extent of dorsal rim trochlea carpalis: 0, ending considerably short of ventral rim; 1, falling only slightly short of ventral rim; 2, equals or exceeds ventral rim. Ericson (1997, char. set A: 64).

157. Carpometacarpus, proximal end, fovea carpalis cranialis (anterior carpal fossa): 0, absent, cranial margin of trochlea carpalis flat or slightly convex (e.g. *Anseranas*); 1, present, cranial margin of trochlea carpalis distinctly concave, but lacking pneumatic foramen; 2, present and contains pneumatic foramen. Worthy and Lee (2008, char. 74).

158. Carpometacarpus, proximal end, fovea carpalis caudalis (cuniform fossa): 0, present, and pneumatic; 1, present and not pneumatic, deep, high dorsal margin bounding it, fossa extending markedly below plane of os metacarpale majus; 2, present and not pneumatic, shallow, not strongly emarginated dorsally. Modified from Livezey (1986, char. 46) and Worthy and Lee (2008, char. 75). A deep fossa appears to be correlated with diving, as it is uniformly present in diving taxa.

159. Carpometacarpus, proximal end, caudal view, position of ventral rim of trochlea carpalis relative to area of synostosis of os metacarpale minus on os metacarpale majus: 0, minor metacarpal extends dorsad of ventral rim; 1, os metacarpale minus entirely ventral to ventral rim of trochlea carpalis, e.g., galliforms.

*160. Carpometacarpus, proximal end, ventral facies, fossa infratrochlearis (internal ligamental fossa): 0, absent, region flat or convex, e.g. *Gallus*; 1, shallow, (e.g. *Anseranas*); 2, deep, extends to at or below level of ventral facies of processus extensorius (e.g. *Dendrocygna*). Worthy and Lee (2008, char. 76). In taxa where the ventral surface of trochlea carpalis is roughly coplanar with the ventral surface of the processus extensorius, e.g. anserines, depth is coded as shallow if depth is c. < 25% width of fossa.

*161. Carpometacarpus, proximal end, ventral facies, ridge linking ventral rim of trochlea carpalis and processus pisiformis, relationship to ventral facies of processus extensorius: 0, rounded profile, little elevated (e.g. *Dendrocygna*); 1, sharp drop-off; 2, overhangs ventral facies of processus extensorius with resultant fovea under ledge (e.g. *Malacorhynchus*). Worthy and Lee (2008, char. 77). *Anhima* is not comparable so treated as missing data. In cracids the pisiform process markedly overhangs the extensor process but the character is restricted to the area on the proximal side of the pisiform process.

162. Carpometacarpus, proximal end, processus extensorius (process metacarpal I, extensor process), proximal margin: 0, perpendicular to, or proximally directed relative to shaft; 1, distinctly distally directed (e.g. *Cygnus*). Livezey (1986, char. 41) and Worthy and Lee (2008, char. 78).

163. Carpometacarpus, proximal end, craniocaudal length processus extensorius in ventral view: 0, short, less than width trochlea carpalis; 1, elongate, equal to or greater than width trochlea carpalis. Modified from Livezey (1986, char. 42; 1996a: char. 56), Worthy and Lee (2008, char. 79). The presence of a rugosity on the processus extensorius is related to fighting behaviour so is not characterised. The spur on carpometacarpi of *Anhima* is an autapomorphy not related to primary length of metacarpal 1 so *Anhinga* is coded as non-comparable.

164. Carpometacarpus, proximal end, caudal facies, os metacarpale minus (metacarpal III): 0, rounded or flattened adjacent to fornix with metacarpal II; 1, distinctly grooved. Modified from Livezey (1986, char. 44; 1996a, char. 59) and Worthy and Lee (2008, char. 80).

165. Carpometacarpus, proximal end, length of metacarpal II from processus alularis (pollical facet) to start of spatium intermetacarpale relative to craniocaudal width (ventral view) of the fused metacarpals II and III: 0, long, ≥ width; 1, short, < width. Worthy and Lee (2008, char. 81).

166. Carpometacarpus, proximal end, facies dorsalis, ligament attachments on trochlea carpalis: 0, with a single distinct scar proximally for the insertion of lig. ulnocarpo-metacarpale dorsale (external ligamental) (e.g. *Anseranas*); 1, with a distinct scar for the external ligament and a second scar for the external scapholunar ligament (Woolfenden 1961: 25) located more distally. From Worthy and Lee (2008, char. 82). This character needs to be assessed with use of a microscope and is difficult or impossible to assess on greasy specimens.

167. Carpometacarpus, proximal end, facies dorsalis, ligament attachment on trochlea carpalis for the insertion of lig. ulnocarpo-metacarpale dorsale (external ligamental), location relative to proximal margin of processus extensorius: 0, primarily lies more proximal, e.g. *Anseranas*; 1, more distal, e.g. *Gallus*.

168. Carpometacarpus, proximal end, dorsal view, M. extensor metacarpi ulnaris or flexor attachment (*sensu* Howard 1929): 0, two distinct rugosities, one adjacent to fornix os metacarpale minus et majus (metacarpal II and III), the other more proximad (e.g. *Anseranas, Biziura*); 1, one rugosity, approximately adjacent to fornix metacarpals II and III; 2, one rugosity, distal to fornix. Modified from Livezey (1986, char. 43; 1996a, char. 57), Worthy and Lee (2008, char. 83). The processus intermetacarpalis of *Gallus* is treated as ‘2’.

169. Carpometacarpus, proximal end, dorsal view, distinct processus intermetacarpalis for M. extensor metacarpi ulnaris: 0, absent; 1, present, e.g. *Gallus*. Ksepka (2009, char. 63). This is lacking in all anseriforms and megapodes. In *Crax* and *Ortalis*, the processus is low more like a tuberculum, not as big as in *Gallus* and could be coded intermediate between absent and present. *Eupiloa* also has a prominent attachment site, an incipient processus.

170. Carpometacarpus, proximal end, ventral view, insertion for lig. ulnocarpo-metacarpale ventralis: 0, not prominent; 1, a prominent tubercle.

171. Carpometacarpus, distal end, synostosis metacarpals II and III, maximum length, measured from distal end of spatium intermetacarpale to facies artic. digitalis minoris (facet for digit III): 0, short, length less than width measured just distad of spatium intermetacarpale (e.g. *Anseranas*, *Malacorhynchus*); 1, long, length ≥ width of synostosis, e.g. *Dendrocygna*. Worthy and Lee (2008, char. 84). In part Bourdon (2011, char. 68).

*172. Carpometacarpus, distal end, facies artic. digitalis minoris et major (facets for digits II and III): 0, facet for digit III extends farther distad than that for digit II, e.g. *Gallus*; 1, facets for digits III and II of equal distal extent; 2, facet for digit III ends proximad to facet for digit II, e.g. *Cereopsis*. See Livezey (1986, char. 45; 1996a, char. 61), Worthy and Lee (2008, char. 85). Contra Ericson (1997), but as per Livezey (1986, 1996a), *Anseranas* is state ‘0’. Flightless taxa coded noncomparable, e.g. *Sylviornis*. In *Eupiloa*, digit 3 extends only a little more distal than digit 2, less so than in other galliforms.

**Pelvis**

173. Pelvis, relative length preacetabular region of synsacrum: 0, long, preacetabular length > 40% synsacral length; 1, short, preacetabular length < 40% synsacral length. Modified from Worthy (2009, char. 141).

174. Pelvis, corpus of the first synsacro-thoracic vertebra: 0, about equally compressed mediolaterally as corpi of the following vertebra; 1, considerably more compressed than following vertebra. Ericson (1997, char set A: 27). Dromornithids compressed (Murray and Vickers-Rich 2004: 153).

175. Pelvis, ilia synostosed to synsacrum in adults: 0, not or only weakly fused, and then only anteriorly; 1, yes, extensively fused. From Ksepka (2009, char. 71).

*176. Pelvis, distinct fenestrae intertransversariae caudad of acetabulum: 0, absent; 1, fenestrae only in distal half of length, e.g. *Cereopsis*; 2, fenestrae present over whole length caudal to acetabulum. Worthy (2009, char. 142).

*177. Pelvis canalis iliosynsacralis: 0, absent, ilia entirely fused with processus spinosus of synsacral vertebrae and each other dorsally; 1, partial fusion of ilia leaving small, narrow paired openings directed caudally; 2, large paired openings caudally. See Ericson (1997, char. set A: 29), Ksepka (2009, char. 69) and Worthy (2009, char. 143). Large openings characterise Tinamidae, Cracidae, Megapodiidae, and Phasianidae. Note that Ericson (1997: Table 1) coded this character the inverse of the states described. *Burhinus* has unfused ilia but the articulated ilia and synsacrum lack the canalis.

178. Pelvis, lateral facies corpus ischia, with pneumatic foramen below the antitrochanter at junction of ischium and acetabulum: 0, no; 1, yes, e.g. *Cereopsis*. Worthy *et al.* (1997, char. 121) and Worthy (2009, char. 144).

179. Pelvis, antitrochanter: 0, with pneumatic openings medially to fossa renalis, or posteriorly into foramen ilioischiadicum; 1, none. Worthy (2009, char. 145).

*180. Pelvis, recessus caudalis fossae (=recessus iliacus): 0, deep, e.g. *Gallus*; 1, shallow and pneumatic; 2, absent. Livezey (1986, char. 120), Ericson (1997, char. set A: 31), Mayr and Clarke (2003, char. 95), Ksepka (2009, char. 75), Worthy (2009, char. 146). Ericson (1997) interpreting the fossa present in *Anseranas* and *Anhima* as a secondary effect of pneumatisation and so coded the recessus as lacking in these taxa, however, they are here treated as shallow and pneumatic.

181. Pelvis, spatium ischiopubicum: 0, dorsoventrally broad, pubis only approaching ischium immediately caudal of foramen obturatum and at processus terminalis ischii; 1, narrow, pubis often partly fused to ischium. From Ericson (1997, char. set A: 30), Ksepka (2009, char. 77).

*182. Pelvis, foramen ilioischiadicum: 0, very short, much <1/2 length ischium from foramen acetabulum; 1, approximately 1/2 length ischium from foramen acetabuli; 2, long, >1/2 length ischium from foramen acetabuli. Worthy (2009, char. 147).

183. Pelvis, foramen ilioischiadicum caudally closed: 0, no; 1, yes. From Mayr and Clarke (2003, char. 94), Ksepka (2009, char. 74). State 1 is characteristic of palaeognaths except *Casuarius*.

184. Pelvis, tuberculum preacetabulare: 0, prominent, creates notch between it and ilium, e.g. *Gallus*; 1, absent or small, with no notch above it. Overlaps Mayr and Clarke (2003, char. 93), Ksepka (2009, char. 72), Worthy (2009, char. 148).

185. Pelvis, pubis: 0, dorsally concave over length; 1, straight or flat. Livezey (1986, char. 115), Worthy (2009, char. 149).

186. Pelvis, pubis, section distad of articulation with distal ischium with flattened caudal expansion, typically rounded in lateral view and with diameter significantly greater than area immediately craniad of it: 0, pubis lacks such caudal expansion; 1, expansion present, e.g. *Cygnus*. Livezey (1986, char. 117), Worthy (2009, char. 150).

**Femora**

187. Femur, constriction of collum femoris in caudal view: 0, not or slight; 1, constricted, neck narrower than ball. Modified from Bourdon et al. (2009, char. 81); Worthy and Scofield (2012, char. 115).

188. Femur, proximal end, facies articularis antitrochanterica, lateromedial plane: 0, surface concave (e.g. *Anseranas*); 1, surface convex. Worthy and Lee (2008, char. 87); Worthy and Scofield (2012, char. 114).

189. Femur, presence of a distinct fossa trochanteris: 0, yes; 1, no. In most galliforms, the trochanter femoris proximally overhangs the facies articularis antitrochanterica enclosing a distinct fossa.

190. Femur, proximal end, crista trochanteris and adjacent cranial facies: 0, cranial facies adjacent to crista flat or shallowly concave; 1, cranial facies deeply concave (e.g. *Anseranas*). Modified from Worthy and Lee (2008, char. 88). The presence of a pneumatic foramen is not considered here, so in *Anhima* where a pneumatic foramen penetrates a flat surface, the characters is coded 0.

191. Femur, proximal end, cranial facies, crista trochanteris, penetrated by pneumatic foramina: 0, no; 1, yes. Mayr and Clarke (2003, char. 98).

192. Femur, cranial facies, pneumatic foramina/fossa adjacent to facies articularis antitrochanterica at base of collum femoris in adults: 0, no; 1, yes. Note: often such fossa are evident in juveniles and disappear in adults – here the character codes its persistence in adults only. Modified Worthy and Holdaway (2002: Appendix 3, char. 49). This feature is distinguished from pneumatism under the crista trochanteris, as seen e.g. in *Leipoa*. Worthy and Scofield (2012, char. 129).

193. Femur, caudal facies, pneumatic openings adjacent to facies articularis antitrochanterica: 0, absent or small; 1, present and large. Modified Worthy and Holdaway (2002: Appendix 3, char. 50), Worthy and Scofield (2012, char. 128).

194. Femur, proximal end, caudal facies, impressiones obturatoriae: 0, a single large scar close to the facies articularis antitrochanterica extending from lateral onto caudal facies; 1, two rugose impressions, the smaller one close to the facies articularis antitrochanterica and a larger one more distally and separated from the former by a sulcus as broad as the impression. This overlaps Bourdon et al. (2009, char. 83).

195. Femur, proximomedial part caudal facies, scar for insertion of M. puboischiofemoralis pars medialis: 0, either absent or a weak crest; 1, a strongly developed, elevated, rugose crest, round or elongate. Note: *Anhima* has a round and prominent insertion of M. puboischiofemoralis pars medialis, here coded (1), because prominence is considered more significant than whether or not it is elongate. Worthy and Scofield (2012, char. 131).

196. Femur, proximal end, cranial view, linea intermuscularis cranialis (cranial intermuscular line) in proximal half: 0, line merges with distal end of cristra trochantris; 1, line passes distal end of cristra trochantris and is discrete and parallel to crista on pretrochanteric surface.

197. Femur, proximal end, cranial facies, trochanter: 0, elongate, extends distally past the level of the caput femoralis a distance exceeding the proximodistal width of the caput femoralis; 1, short, terminates distally at a point only slightly distad of the caput.

198. Femur, proximal end, cranial facies, impression of m. iliofemoralis internus: 0, not or weakly marked; 1, well-marked rugosity.

199. Femur, corpus femoris in caudal view, relative length, lateral and medial margins and least width: 0, shaft short, least width at or proximal of mid length, margins not parallel; 1, elongate, least width at mid length, margins parallel for about middle third of length. Modified from Bourdon et al. (2009, char. 84), Worthy and Scofield (2012, char. 118).

*200. Femur, dorsoventral curvature of shaft, lateral view: 0, straight or slight; 1, moderate curvature of distal third; 2, strong curvature. From Livezey (1986, char. 55), Worthy and Lee (2008, char. 93), and Worthy and Scofield (2012, char. 130).

201. Femur, profile of medial facies in caudal aspect: 0, relatively straight or slight curvature; 1, markedly concave.

*202. Femur, proximal end, lateral facies, insertion area of m. iliotrochantericus caudalis: 0, located at mid depth; 1, in dorsal half of depth, but separated from dorsal margin; 2, on dorsal margin. See Zinoviev (2013) for moas.

203. Femur, caudal facies, presence of tuberosity in area of convergence of crista supracondylaris medialis and linea intermuscularis caudalis, usually distal to mid-length, not insertion of M. caudofemoralis more proximally: 0, none or small, not distinguished from crista, included here taxa where lateral and medial crista do not converge; 1, yes, a distinct tuberosity, may be complex. None in *Dromaius*, large in moas and *Dinornis* autapomorphically has a pair of tuberosities separated by a smooth groove parallel to the axis.

204. Femur, caudal facies, distal half, location of tuberosity or most prominent part crista medialis: 0, centred on shaft; 1, on medial margin of shaft e.g. *Genyornis*; 2, on lateral margin of shaft, e.g. *Paracathartes howardae*. Not Worthy and Scofield (2012, char. 119).

205. Femur, distal end, cranial aspect, orientation of condylus lateralis: 0, not divergent, or only slightly divergent, from axis; 1, markedly divergent.

206. Femur, proximal end, lateral facies, area for the insertion of the m. obturatorius lateralis (=m. obturatorius medialis in Zinoviev 2013) proximally and insertion area of the m. ischiofemoralis more distally: 0, widely separated with gap wider than length of insertion of the m. obturatorius lateralis; 1, adjacent or narrowly separated with gap less than length of insertion of the m. obturatorius lateralis.

207. Femur, distal end, tuber. m. gastrocnemialis lateralis, form: 0, round scar, close to or abutting trochlea fibularis on caudal facies; 1, rounded scar, well separated from trochlea fibularis on caudal facies; 2, an elongate scar/ridge with distinct medial bend, or oriented medially, on caudal facies, may extend proximad of patella sulcus, e.g. most anatids; 3, elongate rugose scar in deep or shallow fossa, traversing caudal facies from lateral edge of trochlea fibularis to proximal end of crista tibiofibularis, merged with ansa m. iliofibularis caudalis; 4, a rugose scar or shallow fossa on the lateral facies proximocranial to the cond. fibularis.

Modified from Worthy and Lee (2008, char. 89) and Bourdon et al. (2009, char. 92) and Worthy and Scofield (2012, char. 127).

208. Femur, distal end, form impressio ansa m. iliofibularis caudalis, usually distal and lateral to tuberculum m. gastrocnemialis lateralis: 0, scar entirely on lateral facies dorsal to trochlea fibularis, e.g. megapodes; 1, scar on lateral facies but also wraps around the caudolateral margin at the proximal side of the trochlea fibularis, e.g. *Porphyrio*; 2, scar on caudal facies above trochlea fibularis and merged with tuberculum m. gastrocnemialis, e.g. dinornithiforms, dromornithids.

The impressio ansa m. iliofibularis caudalis merges with the tuber. m. gastrocnemialis lateralis in dinornithiforms and dromornithids to form a deep scar that extends transversely across the caudal facies just proximal to the trochlea fibularis, referred to as the epicondylar fossa by Worthy and Holdaway (2002: 578) for moas.

209. Femur, caudal aspect, trochlea fibularis with distinct depression immediately proximal of the articular surface: 0, not so; 1, yes. Worthy and Lee (2008, char. 125).

210. Femur, distal extent in caudal view of condylus medialis: 0, approximately equal to that of condylus lateralis, e.g. *Anseranas*; 1, distinctly less than that of external condyle. From Livezey (1986, char. 53), Worthy and Lee (2008, char. 91), Worthy and Scofield (2012, char. 120).

211. Femur, cond. medialis, proportion of maximum distal width: 0, approximately half; 1, clearly greater than half. Note: Assessing the relative size of the distal condyles in this way, avoids issues of using the relative distal prominence of the cond. lateralis, which is compromised by orientation of the condyles to the axis of the shaft and or to reduction of the cond. medialis, as seen in for example *Dromaius* and *Struthio*. Overlaps Lee et al. (1997, char. 44), Worthy and Scofield (2012, char. 121).

212. Femur, cond. medialis, profile in medial aspect: 0, evenly rounded, e.g. *Alectura*; 1, subangular between articular surface of condyle and its cranial surface; 2, dorsoventrally much deeper than long, so proximodistally flattened.

213. Femur, distal end, width sulcus patellaris in cranial view, taken at half the depth of the bounding condyles: 0, broad and flat, wider than condylus lateralis; 1, narrow and deep, less than width condylus lateralis plus trochlea fibularis.

214. Femur, the notch, or fovea tendineus m. tibialis, on the distal end of cond. lateralis, in distal view: 0, notch present; 1, notch absent. From Lee et al. (1997: char. 50) and modified form Worthy and Scofield (2012, char. 133).

215. Femur, distal end, fossa poplitea: 0, shallow, less than half depth medial condyle; 1, deep. See Livezey (1986, char. 56), in part Lee et al. (1997, char. 51), Worthy and Lee (2008, char. 94), Ksepka (2009, char. 79) and Worthy and Scofield (2012, char. 124).

216. Femur, distal end, fossa poplitea, pneumatic: 0, no; 1, yes, large pneumatic foramina are present. Dromornithids are highly pneumatic.

*217. Femur, crista supracondylaris medialis, distinct from its proximal continuation as linea intermuscularis ventralis along the shaft, length: 0, long, greater than width of condylus medialis; 1, short, less than width condylus medialis; 2, crista supracondylaris medialis lacking.

218. Femur, caudal facies, medial view, internal edge of distal end of shaft: 0, smoothly curving, continuous to condyle, e.g. *Leipoa*; 1, interrupted by caudally prominent crista supracondylaris medialis; 2, crista supracondylaris medialis short, and medial profile notched. Modified from Livezey (1986, char. 59), and Worthy and Lee (2008, char. 96).

219. Femur, distal end, caudal view, trochlea fibularis, distal extent: 0, equal with condylus lateralis; 1, shorter, merges distally with the lateral side of the condyle proximal to the distal end of the condyle forming a notch.

220. Femur, trochlea fibularis, form and orientation of articular surface: 0, directed caudally, roughly parallel to shaft, with lateral margin proximally merging with lateral facies of shaft smoothly cranial to the impressio ansa m. iliofibularis caudalis; 1, proximal part rotated cranially and facet directed proximally at low angle to shaft, and forms a prominence markedly offset from the lateral facies. Modified from Bourdon et al. (2009, char. 88), Worthy and Scofield (2012, char. 122).

221. Femur, extent impressio ligamenti cruciati cranialis: 0, well marked and excavated into caudodistal facies of cond. lateralis; 1, poorly defined and not extending onto cond. lateralis. Modified from Bourdon et al. (2009, char. 89) and Worthy and Scofield (2012, char. 123).

**Tibiotarsus**

222. Tibiotarsus, proximal end, proximal projection of crista cnemialis cranialis: 0, equal with or extending slightly proximal of crista patellaris; 1, extending well proximal of crista patellaris. In part Ericson (1997, char. set A: 66). No attempt is made here to segregate moderate and marked projection which can often relate to diving ability (Worthy and Lee 2008).

223. Tibiotarsus, proximal end, impressio lig. collateralis medialis: 0, low, not prominent (e.g. *Anseranas*); 1, prominent on facies (e.g. *Gallus*). From Worthy and Lee (2008, char. 97).

224. Tibiotarsus, proximal end, medial facies in medial view, linea extensoria (intermuscular line) extending from crista cnemialis cranialis, position relative to impressio lig. collateralis medialis: 0, intermuscular line straight from crista cnemialis cranialis to near mid length, separates lateral and cranial facies, is well separated from impressio (e.g. *Anseranas*, *Gallus*); 1, proximally the intermuscular line is shifted caudally to be narrowly separated from/abuts impressio, so that cranial facies is visible in medial view and anterior facies between ligament attachment and fibular crest is markedly convex. After Worthy and Lee (2008, char. 98).

225. Tibiotarsus, proximal end, alignment base of crista cnemialis cranialis: 0, aligned with axis of shaft; 1, deflected laterally from axis. See Livezey (1986, char. 63; 1996a, char. 70). After Worthy and Lee (2008, char. 102). This character refers to the base of the crista not its cranially projecting part, which is the subject of character 223.

226. Tibiotarsus, crista cnemialis cranialis, proximal tip: 0, rolled or bent laterally relative to rest of crista; 1, straight. Note: *Apteryx* has a low crista cnemialis cranialis, but the lateral inflection of it is taken to be homologous to (0). *Rhea* and *Struthio* are non comparable. Modified from Worthy and Holdaway (2002, appendix 3, char. 56). Worthy and Scofield (2012, char 140).

*227. Tibiotarsus, proximal end, lateromedial constriction between cnemial crests and articular facets, breadth of crista patellaris: 0, not constricted, crista patellaris broad; 1, somewhat constricted, crista patellaris short; 2, markedly constricted, crista patellaris indistinct. These features are part of a single complex so are treated here as a single character. Modified from Bourdon et al. (2009, char. 93), Lee et al. (1997, char. 33), and Worthy and Scofield (2012, char. 137).

*228. Tibiotarsus, cranial aspect, angle formed by the lateral and ventral margins of the crista cnemialis lateralis: 0, angle >100° ie widely obtuse; 1, angle <100° and approximately right angle, e.g. Gallus; 2, angle < 60 degrees and clearly acute. See Worthy and Holdaway (2002: 588, char. 57). *Rhea* and *Struthio* are inapplicable because of constriction of the cnemial crests associated with character 227, state 2. Worthy and Scofield (2012, char. 139).

*229. Tibiotarsus, crista cnemialis cranialis (as distinct from the intermuscular line that may continue from its end), distal extent and location on shaft: 0, crista extends distal to the proximal end of the crista fibularis; 1, crista ends level with or just proximal to the proximal end of the crista fibularis, e.g. *Gallus*, *Anseranas*; 2, crista is well separated proximally from crista fibularis. Worthy and Scofield (2012, char. 141).

*230. Tibiotarsus, length of crista fibularis: 0, less than <20% of tibiotarsus length; 1, 20-25% of tibiotarsus length; 2, > 25% of tibiotarsus length. See Worthy and Holdaway (2002: 588, char. 59). Modified from Worthy and Scofield (2012, char. 142).

*231. Tibiotarsus, distal end, epicondylus medialis with internal ligamental prominence: 0, pronounced, visible in anterior view (e.g. *Cereopsis*); 1, present, not pronounced, occluded by rim of condylus medialis in anterior view (e.g. *Gallus*); 2, absent. Worthy and Lee (2008, char. 99).

232. Tibiotarsus, epicondylus medialis, form: 0, rounded prominence with no sharp margins; 1, enlarged and plate-like, bounded distally by deep depressio epicondylaris medialis (modified from Cracraft 1974: 501, 506; Lee et al. 1997, char. 39). From Bourdon et al. (2009, char. 105), Worthy and Scofield (2012, char. 155).

233. Tibiotarsus, cond. medialis, presence of ligamental pit cranially on the external facies: 0, none or shallow; 1, yes and deep, e.g. *Dromaius*. Modified from Bourdon et al. (2009, char. 102), see Cracraft (1974, char. 7), Lee et al. (1997, char. 39) and Worthy and Scofield (2012, char. 152).

234. Tibiotarsus, lateral view, cranial side cond. lateralis: 0, merges with the shaft smoothly in a wide angle; 1, abruptly joins shaft, may form notch. Modified from Bourdon et al. (2009, char. 100), Lee et al. (1997, char. 36), and Worthy and Scofield (2012, char. 150).

235. Tibiotarsus, distal view, cranial projection of condyles: 0, condylus medialis projects markedly relative to condylus lateralis; 1, medial condyle has roughly similar projection to lateral one, i.e. = or <10% taller. See Livezey (1986, char. 64), Lee et al. (1997, char. 38), Livezey & Zusi (2006, char. 2144), Worthy and Lee (2008, char. 103), Bourdon et al. (2009, char. 101), and Worthy and Scofield (2012, char. 151).

*236. Tibiotarsus, conspicuous scar for lig. collaterale medialis proximocaudal to epicondylus medialis: 0, absent; 1, present short; 2, present and elongate. From Bourdon et al. (2009, char. 103) and Worthy and Scofield (2012, char. 153).

237. Tibiotarsus, distal end, junction of crista trochlea cartilaginous tibialis and rim of condylus medialis marked by distinct shallow notch, usually at mid-depth: 0, no (e.g. *Anseranas*); 1, yes (e.g. *Gallus*, *Alectura*). See Livezey (1986, char. 62) and Worthy and Lee (2008, char. 100).

238. Tibiotarsus, cranial view, form junction of medial shaft facies and cond. medialis: 0, shaft with medial profile straight or concave immediately proximal to cond. medialis; 1, shaft medially convex just proximal to the condylus, due to a flange like projection, e.g. *Leipoa*, emu. From Bourdon et al. (2009, char. 104), see Worthy and Holdaway (2002, fig. A.3, appendix 3, char. 61), and Worthy and Scofield (2012, char. 154).

239. Tibiotarsus, distal end, area intercondylaris, presence of impressio ligamenti intercondylaris (= impressio for lig. anticum): 0, absent, e.g. *Dromaius*, *Genyornis*; 1, present of variable size, e.g. *Gallus*, *Anseranas*. This ligament is absent in moas (Zinoviev 2013).

*240. Tibiotarsus, distal end, area intercondylaris, lateral to the pons supratendineus, impressio ligamentum meniscotibiale, = lig. meniscotibiale intertarsi of Zinoviev (2013), not impressio ligamenti intercondylaris, contra Worthy and Scofield (2012): 0, forms prominent facet proximal to fossa intercondylaris; 1, distinct low facet abutting fossa intercondylaris, e.g. *Dromaius*, *Gallus*; 2, insertion does not form facet or is indistinct. From Bourdon et al. (2009, char. 97). Note: this is the tuberculum supratrochlearis of Mourer-Chauviré (2008) and is the lig. meniscotibiale intertarsi of Zinoviev (2013), not impressio ligamenti intercondylaris, contra Worthy and Scofield (2012). From Worthy and Scofield (2012, char. 144).

241. Tibiotarsus, distal end, area intercondylaris, breadth impressio ligamenti intercondylaris (for lig. tibiometatarsale intercondylare): 0, confined between condyles, either essentially centrally located e.g. *Gallus* or slightly offset medially; 1, extends medially by excavation caudal to the cond. medialis e.g. *Cereopsis*. See Lee et al. (1997, char. 40), Livezey and Zusi (2006, char. 2168), Bourdon et al. (2009, char. 106) and Worthy and Scofield (2012, char. 145). Where the impressio ligamenti intercondylaris is absent, e.g. *Dromaius*, the state is coded as non-applicable (-).

242. Tibiotarsus, pons supratendineus: 0, present; 1, absent. After Lee et al. (1997, char. 35), Mayr and Clarke (2003, char. 73) and Worthy and Scofield (2012, char. 146).

*243. Tibiotarsus, distal end, distal opening of canalis extensorius opens towards impressio ligamenti intercondylaris between condylus lateralis and condylus medialis: 0, no, directed towards and broadly overlaps in lateromedial plane with condylus medialis (e.g. ratites); 1, no, partly overlaps in lateromedial plane with condylus medialis (e.g. galliforms); 2, yes, no overlap in lateromedial plane with condylus medialis. From Ericson (1997, char. set A: 69) and in part Worthy and Scofield (2012, char. 147).

244. Tibiotarsus, medial displacement of cond. medialis relative to facies medialis of shaft: 0, slight; 1, pronounced. Equals Livezey and Zusi (2006, char. 2150) and Worthy and Scofield (2012, char. 149).

245. Tibiotarsus, distal end, width incisura intercondylaris: 0, broad; 1, narrow, width subequal to canalis extensorius, e.g. *Gallus*; 2, incisura absent, e.g. *Dromaius*. Ericson (1997, char. set A: 69). This is in part Bourdon’s (2011, char. 69) identified as an apomorphy of Odontoanseres. Other components of Bourdon’s (2011, char. 69) are addressed in char 241.

246. Tibiotarsus, distal end, sulcus m. fibularis (groove for peroneus profundus): 0, faces cranially; 1, faces laterally, e.g. galliforms.

247. Tibiotarsus, distal end, lateral insertion of the transverse ligament (= retinaculum extensorium tibiotarsi) adjacent to pons supratendineus/canalis extensorius: 0, yes, e.g. *Leipoa*; 1, no, located more laterad, and aligned more transversely e.g. *Dromaius*.

248. Tibiotarsus, distal end, distal opening of canalis extensorius, plane of long axis: 0, aligned across shaft, e.g. anseriforms; 1, aligned transversely, e.g. *Leiopoa*, *Gallus*.

**Tarsometatarsus**

*249. Tarsometatarsus, relative length: 0, less than 95% femur length; 1, approximately equal to femur (~ ±5%); 2, more than 105% femur length. From Worthy and Holdaway (2002); in part Bourdon et al. (2009, char. 121 & 123), Worthy and Lee (2008, char. 118), Worthy and Scofield (2012, char. 167).

250. Tarsometatarsus, cotyla medialis dorsoplantarly elongated, protruding dorsal to cotyla lateralis: 0, no; 1, yes. From Bourdon et al. (2009, char. 111). Worthy and Scofield (2012, char. 159).

251. Tarsometatarsus, facies dorsalis and cotyla medialis: 0, dorsal margin is a crest that separates the cotyla from the dorsal facies; 1, dorsally the cotyla laps onto the dorsal facies, forming a facet that articulates with an opposing facet on the tibiotarsus when the bird is sitting, with a distinct groove between this facet and the eminentia intercotylaris. In part Bourdon et al. (2009, char. 112), Worthy and Scofield (2012, char. 160).

252. Tarsometatarsus, distinct flange-like processus on plantarmedial edge of cotyla medialis: 0, no; 1, yes. From Bourdon et al. (2009, char. 113), Worthy and Scofield (2012, char. 161).

253. Tarsometatarsus, proximal end, plantarolateral side of cotyla medialis: 0, rim elevated proximally; 1, no proximal elevation of rim at this point. Note: the elevation of the rim appears to be the salient point of Bourdon’s (2011, char. 70), which identified an elevated rim and the location of crista medialis hypotarsi in line with plantaromedial corner of cotyla medialis as an apomorphy of Odontoanseres, as the crista location mentioned also occurs in galliforms, which were coded as lacking this apomorphy.

254. Tarsometatarsus, eminentia intercotylaris, proximal view, relative to adjacent cotylae is prominent dorsally: 0, yes (*Anseranas*); 1, no (*Alectura*). Modified from Cracraft (1974, 503), Lee et al*.* (1997, char. 28), Bourdon et al. (2009, char. 114) and Worthy and Scofield (2012, char. 162).

255. Tarsometatarsus, eminentia intercotylaris, lateral view, relative to adjacent area intercotylaris is prominent proximally: 0, yes (*Anseranas*); 1, no (megapodes). Dorsal prominence is not correlated with proximal prominence as *Gallus* has its eminentia proximally prominent but not dorsally.

256. Tarsometatarsus, proximal end, crista medialis hypotarsi, or deepest one, caudal extent in proximal view: 0, depth of medial side of crista ≥ depth of cotyla medialis (e.g. *Anseranas*); 1, depth noticeably < depth of cotyla medialis. Modified from Livezey (1986, char. 70), Worthy and Lee (2008, char. 106).

*257. Tarsometatarsus, proximal end, hypotarsus, width adjacent to cotylae: 0, distinctly less than ½ proximal width; 1, approximately ½ of proximal width; 2, distinctly more than ½ proximal width. Worthy and Lee (2008, char. 107).

258. Tarsometatarsus, hypotarsus, major hypotarsal ridge, distal end: 0, markedly hooked caudodistally forming notch; 1, ridge terminates abruptly, drops steeply to shaft; 2, ridge terminates by gradually lowering to shaft. Worthy and Lee (2008, char. 117).

259. Tarsometatarsus, hypotarsus, number of hypotarsal ridges: 0, four (*Anseranas* and anhimids have two vestigial inner ridges); 1, three ridges (galliforms); 2, two ridges; 3, one centrally located ridge. Modified from Cracraft (1974, 502), Lee et al*.* (1997, char. 27), Worthy and Scofield (2012, char. 163). The lateral ridge is much reduced in *Gallus*. *Megavitiornis* considered to have a vestigial lateral ridge on the side of the robust medial ridge. *Burhinus* has two main ridges plus a reduced lateral one. This character is not necessarily a morphocline, as fusion of numerous close ridges could directly produce a single ridge.

***260. Tarsometatarsus, hypotarsus, number of hypotarsal canals: 0, none; 1, one canal, for m. flexor digitorum longus (fdl); 2, two canals, i.e. for fdl and one for tendons for m. flexor perforatus digiti II and m. flexor perforans et perforatus digiti II (pII–ppII), located plantarlateral to fdl. Note: Overlaps** Ksepka (2009, char. 81). **In *Gallus*, near enclosure of canal for pII–ppII is intermediate between the broadly open state in megapodes and the closed condition in Odontophoridae and some Tetraonini (*Bonasa*, *Tympanuchus*), among phasianids (Holman 1964).**

*261. Tarsometatarsus, proximal end, fossa parahypotarsalis medialis: 0, very large deep and broad, >1/2 width at distal end of hypotarsus (e.g. *Leipoa*); 1, deep but narrow, <1/2 width at distal end hypotarsus, (e.g. *Anseranas*); 2, shallow, surface from medial calcaneal ridge to anterior margin of medial shaft concave (e.g. *Cereopsis*); 3, absent, surface from medial calcaneal ridge to anterior margin of medial facies shaft flat or convex. From Worthy and Lee (2008, char. 108). State 0 correlates with a dorsoventrally thin shaft typical of megapodes.

*262. Tarsometatarsus, corpus tarsometatarsi, sulcus extensorius: 0, absent e.g. *Alectura*, *Leipoa*; 1, shallow and broad proximally, flattens out distally, e.g. *Gallus*, *Cereopsis*; 2, deep, well defined at mid-length, extending into distal half. See Lee et al*.* (1997, char. 30), Bourdon et al. (2009, char. 122), Worthy and Lee (2008, char. 110), Worthy and Scofield (2012, char. 168).

*263. Tarsometatarsus, tuberositas M. tibialis cranialis; 0, a single tuberosity distal to medial foramen which may have indications of the more lateral tendon as a very much smaller scar on its edge; 1, two distinct tuberosities; 2, two distinct tuberosities fused as one in the base of the extensor sulcus, with foramen passing through it, e.g. *Dromornis*. Worthy and Scofield (2012, char. 172).

264. Tarsometatarsus, tuberositas M. tibialis cranialis; 0, confined within extensor sulcus; 1, dorsally prominent.

265. Tarsometatarsus, foramina vascularis proximalis: 0, medial and lateral foramina of roughly equal size, e.g. *Anseranas*, *Anhima*; 1, medial foramen considerably larger than lateral one (e.g. *Alectura*, *Leipoa*); 2, a single fossa with foramina in it.

266. Tarsometatarsus, impressiones retinaculi extensorii: 0, form two short crests proximal to the foramina vascularis proximalis; 1, two elongated crests with impressio retinaculum extensorii medialis extending distally on the shaft distad of the medial foramen vascularis proximalis.

267. Tarsometatarsus, corpus tarsometatarsi, dorsal view: 0, shaft long, sides essentially parallel over middle third of length; 1, shaft relatively shorter for bone width, narrowest in distal third of length and widen proximally. Not Character 165 of Worthy and Scofield (2012).

268. Tarsometatarsus, plantar facies, development of crista plantares medialis et lateralis: 0, little or no development; 1, well developed especially laterally. From Lee et al*.* (1997, char. 31) and Bourdon et al. (2009, char. 118 & 121), Worthy and Scofield (2012, char. 166).

*269. Tarsometatarsus, shaft, width at mid-length: 0, wider than deep; 1, width approximately equals depth; 2, depth exceeds width. Worthy and Lee (2008, char. 116).

270. Tarsometatarsus, dorsal facies, distal shaft: 0, convex dorsally; 1, flat or concave, e.g. *Gallus*. Note: this separates megapodes from phasianids.

271. Tarsometatarsus, fossa metatarsi I: 0, present, well-marked; 1, absent or obsolete. See Livezey (1986, char. 71). Worthy and Lee (2008, char. 113).

*272. Tarsometatarsus, fossa metatarsi I, metatarsal articular facet: 0, absent; 1, present, does not protrude mesad of shaft; 2, present, large and protrudes mesad of shaft.

*273. Tarsometatarsus, dorsal view, trochlea metatarsi II, distal extent: 0, equal or greater than trochlea metatarsi IV (e.g. *Leipoa*); 1, proximal to trochlea metatarsi IV, but overlaps incisura intertrochlearis lateralis (e.g. *Anseranas*, *Cereopsis*); 2, proximal to incisura intertrochlearis lateralis (e.g. *Anser*, most anatids). Modified from Livezey (1986, char. 68; 1996a, char. 75); see also Worthy et al. (1997), Worthy and Lee (2008, char. 105), and Ksepka (2009, char. 84).

274. Tarsometatarsus, distal end, trochlea metatarsi II, central groove dorsally and distally: 0, groove absent (e.g. *Anseranas*); 1, groove present, so distal margin notched. See Livezey (1986, char. 74; 1996a, char. 76), Worthy and Lee (2008, char. 109), Worthy and Scofield (2012, char. 173). Note: In the megapodes and *Gallus*, development of a flange plantarly from the medial edge of the trochlea creates a notch in distal view, but the trochlea lacks a central groove so these taxa are coded (0).

275. Tarsometatarsus, anterior end of canalis interosseus distalis: 0, roofed over entirely in bone, not visible in dorsal view (e.g. *Anseranas*); 1, largely or completely exposed dorsally by reduction in bony covering. Livezey (1986, char. 69). Worthy and Lee (2008, char. 112).

276. Tarsometatarsus, distal end, trochlea metatarsi II outer rim: 0, expanded medially and/or caudally as a flange (e.g. *Anseranas*, *Leipoa*); 1, flattened medial facies, lacking flange. Modified from Livezey (1986, char. 73), Worthy and Lee (2008, char. 114).

277. Tarsometatarsus, distal end, plantar opening of foramen vasculare distale: 0, opens flush onto plantar surface; 1, directed distoplantarly, so partially recessed into incisura intertrochlearis lateralis. Livezey (1986, char. 77), Worthy and Lee (2008, char. 115). The recessed incisura is an apomorphy of Anatidae. Also, this is partly Bourdon’s (2011, char. 71) in which the described state was identified as an synapomorphy of Odontoanseres, however, her character conflated [?ventral] prominence of the trochlea III, elongation and plantar shape of Trochlea III, with the character considered here.

*278. Tarsometatarsus, trochlea metatarsi II, dorsal view, point of maximum medial projection exclusive of plantar flange: 0, distal to maximum proximal extent of incisura intertrochlearis medialis; 1, level with maximum proximal extent of incisura; 2, proximal to incisura. See Worthy and Holdaway (2002, char. 67), after Worthy and Scofield (2012, char. 170).

*279. Tarsometatarsus, foramen vasculare distale: 0, large; 1, small and distinct; 2, tiny and indistinct; 3, absent. Worthy and Scofield (2012, char. 171).

*280. Pes, number of digits: 0, four digits; 1, three digits, II, III and IV; 2, two digits (III & IV). Derived from Bourdon et al. (2009, char. 125). Worthy and Scofield (2012, char. 174).

281. Ossa digiti IV, intermediate phalanges gradually shorten towards phalanx ungualis, so that phalanx just proximal to the latter is either wider than long or nearly square in shape: 0, no; 1, yes. From Bourdon et al. (2009, char. 126). Worthy and Scofield (2012, char. 175).

282. Digitus IV pedis, number of phalanges: 0, five; 1, four. See Worthy and Holdaway (2002, Appendix 3, char. 72), and modified from Bourdon et al. (2009, char. 127), Worthy and Scofield (2012, char. 176).

*283. Ungual phalanges, digit 3, width and depth at midlength: 0, depth > width; 1, depth approximately = to depth (+-5%); 2, depth < width.

284. Incubation type: 0, Endothermic, body heat used to incubate eggs; 1, ectothermic, environmental heat used to incubate eggs.

*285. Webbing between toes (tela interdigitalis), excluding hallux: 0, lacking; 1, rudimentary; 2, semipalmate; 3, palmate. From Livezey (1997). We note that the cracids we sampled have rudimentary webbing.

**References**

Baumel, J.J. and Witmer, L.M. 1993. Osteologia. In *Handbook of Avian Anatomy: Nomina Anatomica Avium*, 2^nd^ Edition, ed. J.J. Baumel, A.S. King, J.E. Breazile, H.E. Evans & J.C. Vanden Berge. *Publications of the Nuttall Ornithological Club* 23: 45–132.

Bourdon, E. 2011. The pseudo-toothed birds (Aves, Odontopterygiformes) and their bearing on the early evolution of modern birds; pp. 209–234 in Dyke G.J. and Kaiser G.W. (eds.), Living Dinosaurs: The Evolutionary History of Modern Birds. John Wiley and Sons, Ltd., Chichester, United Kingdom.

Bourdon, E., de Ricqlès, A. and Cubo, J. 2009. A new transantarctic relationship: morphological evidence for a Rheidae-Dromaiidae-Casuariidae clade (Aves: Palaeognathae, Ratitae). *Zoological Journal of the Linnean Society* 156: 641–663. doi: 10.1111/j.1096-3642.2008.00509.x

Cracraft, J. 1974. Phylogeny and evolution of the ratite birds. *Ibis 116*: 494–521.

Cracraft, J. and Clarke, J. 2001. The basal clades of modern birds. In: Gauthier, J., Gall, L.F. eds. *New perspectives on the origin and early evolution of birds*. New Haven, CT: Yale University Press, 143-156.

Elzanowski, A. and Stidham, T.A. 2010. Morphology of the quadrate in the Eocene anseriform *Presbyornis* and the extant Galloanserine birds. *Journal of Morphology* 271: 305–323. doi: 10.1002/jmor.10799

Ericson, P.G.P. 1996. The skeletal evidence for a sister-group relationship of anseriform and galliform birds – a critical evaluation. *Journal of Avian Biology* 27: 195–202.

Ericson, P.G.P. 1997. Systematic relationships of the Palaeogene family Presbyornithidae (Aves: Anseriformes). *Zoological Journal of the Linnean Society* 121: 429–483.

Hiroshige, M. and Yoshikazu, H. 2007. Myology and osteology of the Whooper Swan *Cygnus cygnus* (Aves: Anataidae). Part 1. Muscles attached to the sternum, coracoid, clavicle, scapula and humerus. *Bulletin of the Gunma Museum of Natural History* 11: 7–14.

Holman, J. A. 1964. Osteology of gallinaceous birds. *Quarterly Journal of the Florida Academy of Sciences* 27: 230–252.

Houde, P.W. 1988. Paleognathous birds from the early Tertiary of the Northern Hemisphere. Publications of the Nuttall Ornithological Club 22: 1–148.

Howard, H. 1929. The avifauna of Emeryville Shellmound. *University of California, Publications in Zoology* 32: 301–394.

Ksepka, D.T. 2009. Broken gears in the avian molecular clock: new phylogenetic analyses support stem galliform status for *Gallinuloides wyomingensis* and rallid affinities for *Amitabha urbsinterdictensis*. Cladistics 25: 173–197.

Lee, K., Feinstein, J. and Cracraft, J. 1997. The phylogeny of ratite birds: resolving conflicts between molecular data and morphological data sets. In: Mindell D ed. Avian molecular evolution and molecular systematics. New York, Academic Press. Pp. 173–211.

Livezey, B.C. 1986. A phylogenetic analysis of Recent anseriform genera using morphological characters. *Auk* 105: 681–698.

Livezey B.C. 1996a. A phylogenetic analysis of geese and swans (Anseriformes: Anserinae), including selected fossil species. *Systematic Biology* 45: 415–450.

Livezey B.C. 1996b. A phylogenetic reassessment of the tadornine-anatine divergence (Aves: Anseriformes: Anatidae). *Annals of the Carnegie Museum* 65: 27–88.

Livezey, B.C. and Zusi, R.L. 2006. Higher-order phylogeny of modern birds (Theropoda, Aves: Neornithes) based on comparative anatomy: I. – Methods and characters. *Bulletin of the Carnegie Museum of Natural History* 37: 1–556.

Mayr, G. and Clarke, J. 2003. The deep divergences of neornithine birds: a phylogenetic analysis of morphological characters. *Cladistics* 19: 527–553.doi: 10.1111/j.1096-0031.2003.tb00387.x

Mourer-Chauviré, C. 1992. The Galliformes (Aves) from the Phosphorites du Quercy (France): Systematics and Biostratigraphy. *Natural History Museum of Los Angeles County, Science Series* 36: 67–95.

Mourer-Chauviré, C. 2008. Birds (Aves) from the Early Miocene of the Northern Sperrgebiet, Namibia. *Memoir Geol. Surv. Namibia* 20: 147–167.

Murray, P.F. and Megirian, D. 1998. The skull of dromornithid birds: anatomical evidence for their relationship to Anseriformes. *Records of the South Australian Museum* 31:51–97.

Murray, P.F. and Vickers-Rich, P. 2004. Magnificent mihirungs: the colossal flightless birds of the Australian dreamtime. Bloomington: Indiana University Press, 1–410 pp.

Olson, S.L. and Feduccia, A. 1980. *Presbyornis* and the origin of the Anseriformes (Aves: Charadriomorphae). *Smithsonian Contributions to Zoology* 323: 1–24.

Parker, T.J. 1895. On the cranial osteology, classification, and phylogeny of the Dinornithidae. *Transactions of the Zoological Society of London* 13: 373–428. doi: 10.1111/j.1096-3642.1895.tb00044.x

Parkes, K.C. and Clark, G.A. 1966. An additional character linking ratites and tinamous, and an interpretation of their monophyly. *Condor* 68: 459–471.

Pycraft, W.P. 1900. On the morphology and phylogeny of the Palaeognathae (Ratitae and Crypturi) and Neognathae (Carinatae). Transactions of the Zoological Society of London 15: 149–290. doi: 10.1111/j.1096-3642.1900.tb00023.x

Raikow, R.J. 1971. The osteology and taxonomic position of the white backed duck, *Thalassornis leuconotus*. *Wilson Bulletin* 83: 270–277.

Weber, Von E. 1993. Zur evolution basicranialer Gelenke bei Vögeln, insbesondere bei Hühner- und Entenvögeln (Galloanseres). *Zeitschrift fur Zoologische Systematik und Evolutionsforschung* 31: 300–317.

Woolfenden, G.E. 1961. Postcranial osteology of the waterfowl. *Bulletin of the Florida State Museum (Biological Sciences)* 6: 1–129.

Worthy, T.H. and Holdaway, R.N. 2002. The lost world of the moa: prehistoric life of New Zealand. Bloomington, IN, Indiana University Press. Xxxiii, 718 pp.

Worthy, T.H. and Lee, M.S.Y. 2008. Affinities of Miocene (19–16 Ma) waterfowl (Anatidae: *Manuherikia*, *Dunstanetta* and *Miotadorna*) from the St Bathans Fauna, New Zealand. *Palaeontology* 51: 677–708.

Worthy, T.H. and Scofield, R.P. 2012. Twenty-first century advances in knowledge of the biology of moa (Aves: Dinornithiformes): a new morphological analysis and diagnoses revised. *New Zealand Journal of Zoology* 39: 87–153.

Worthy, T.H. 2009. Descriptions and phylogenetic relationships of two new genera and four new species of Oligo-Miocene waterfowl (Aves: Anatidae) from Australia. *Zoological Journal of the Linnean Society* 156: 411–454.

Worthy, T.H., Holdaway, R.N. Sorenson, M.D. and Cooper, A.C. 1997. Description of the first complete skeleton of the extinct New Zealand goose *Cnemiornis calcitrans* Owen, (Aves: Anatidae), and a reassessment of the relationships of *Cnemiornis*. *Journal of Zoology, London* 243: 695–723.

Zinoviev, A.V. 2013. Notes on the pelvic and hindlimb myology and syndesmology of *Emeus crassus* and *Dinornis robustus* (Aves: Dinornithiformes). Pp. 253–278. *In*: Göhlich, U.B. & Kroh, A. (eds) *Proceedings of the Eighth International Meeting of the Society of Avian Paleontology and Evolution*, *Vienna, 11–16 June, 2012*. (Naturhistorisches Museum Wien: Vienna).

Zusi, R.L. and Livezey, B.C. 2000. Homologies and phylogenetic implications of some enigmatic cranial features in galliform and anseriform birds. *Annals of Carnegie Museum* 69: 157–193.
