# Supplementary material for: Osteology Supports a Stem-Galliform Affinity for the Giant Extinct Flightless Bird Sylviornis neocaledoniae (Sylviornithidae, Galloanseres)
Source: PLoS One. 2016 Mar 30;11(3):e0150871. doi: 10.1371/journal.pone.0150871 (PMC4814122; doi:10.1371/journal.pone.0150871)
Supplement: S4 File — (DOCX) [file pone.0150871.s004.docx]

Supplementary data for PCA analyses


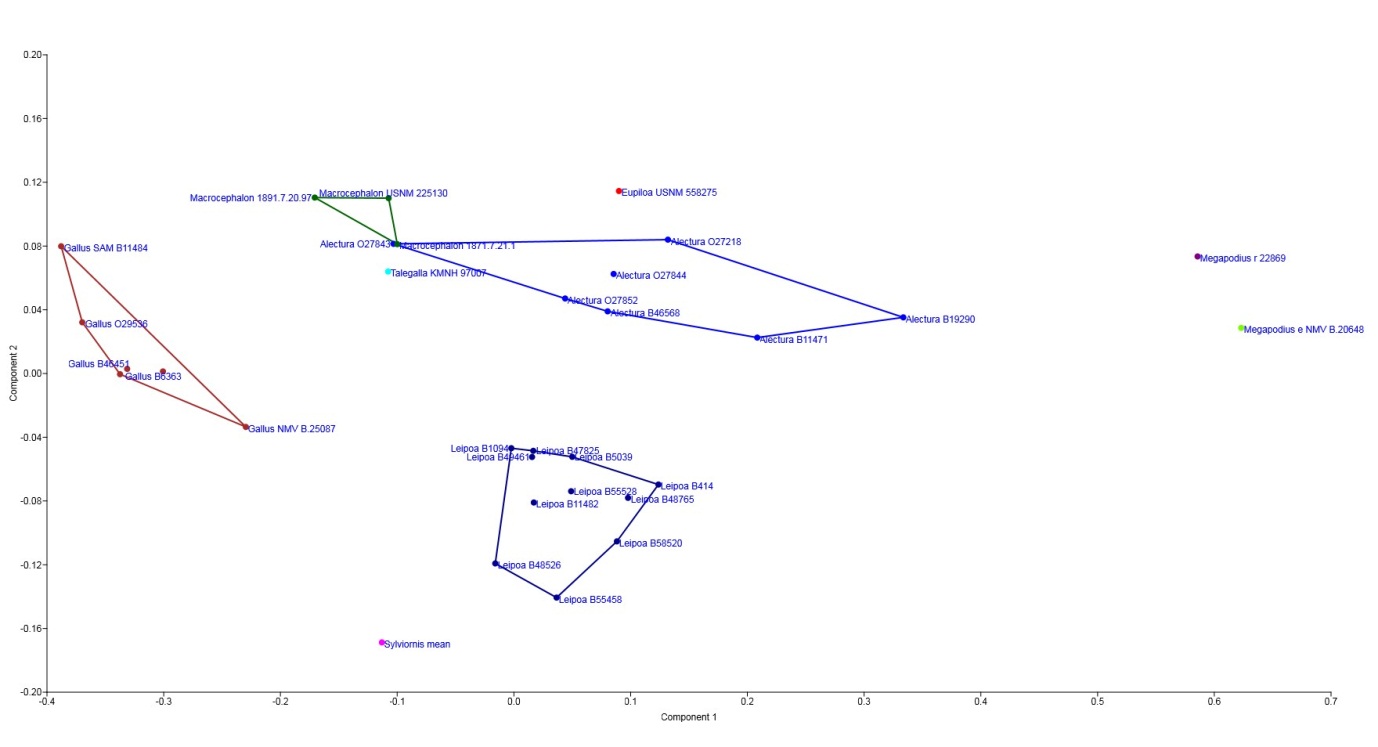


SI 4: Figure 1. The PCA with specimen identifiers


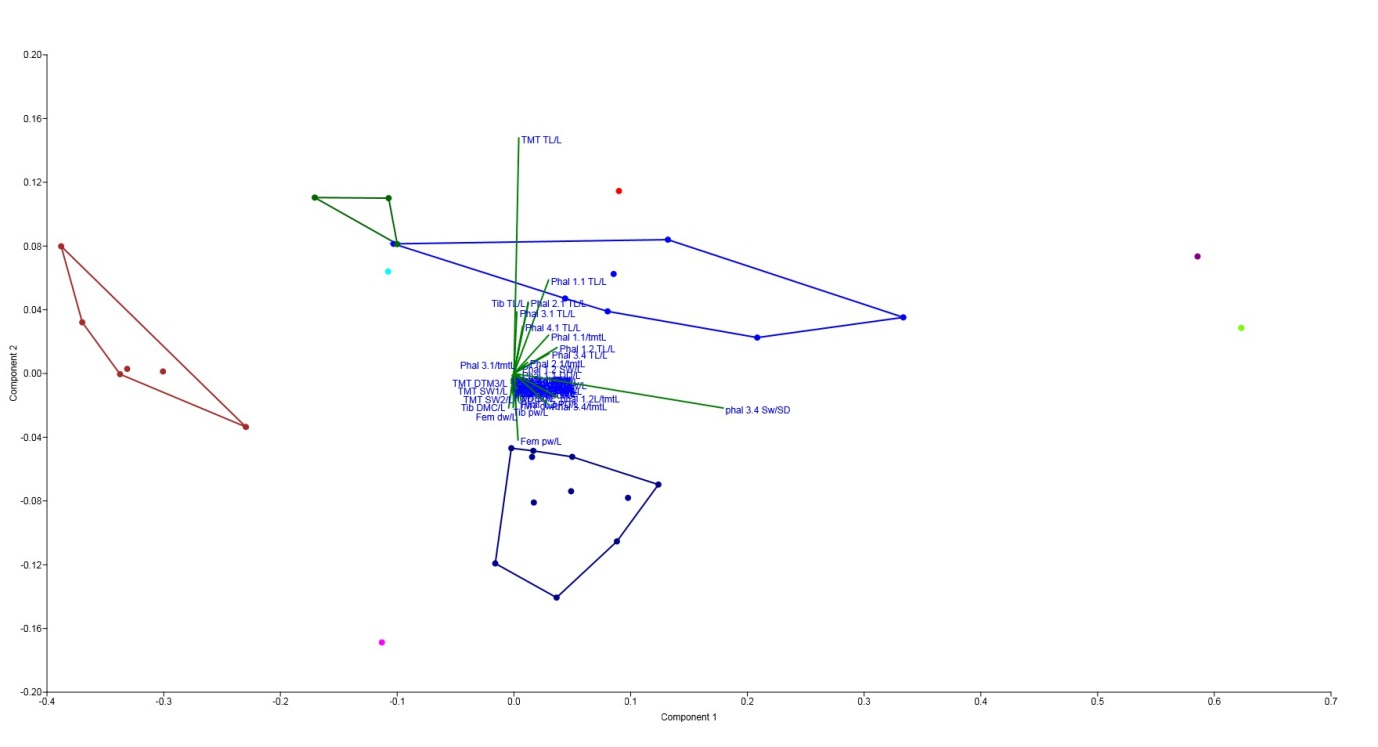


SI 4: Figure 2. A biplot showing the impact of individual variables on the PCA.


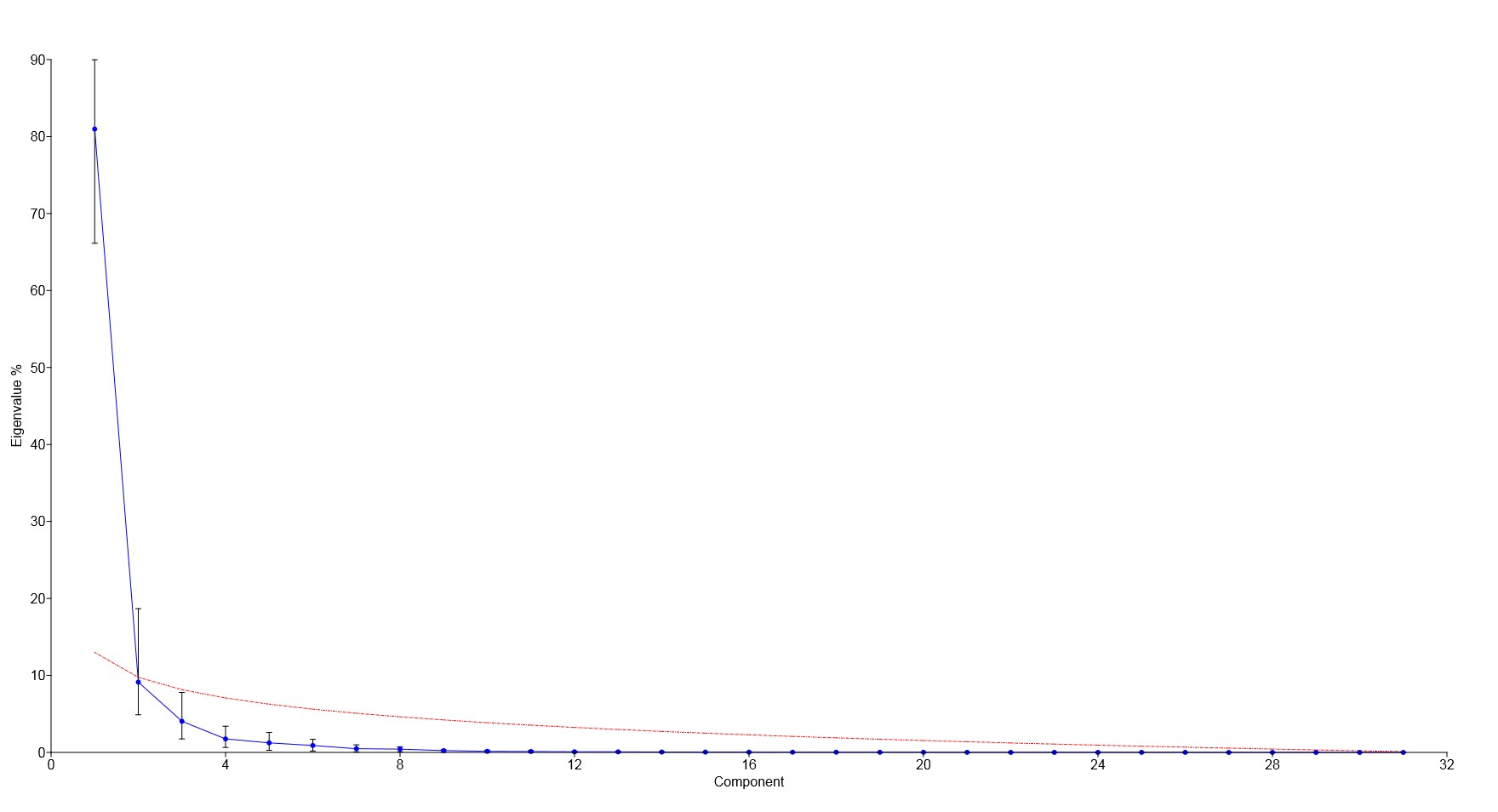


SI 4: Figure 3. A Scree plot showing the percentage of variation each principal component contributes: PC1 and PC2 account for most of the variation.


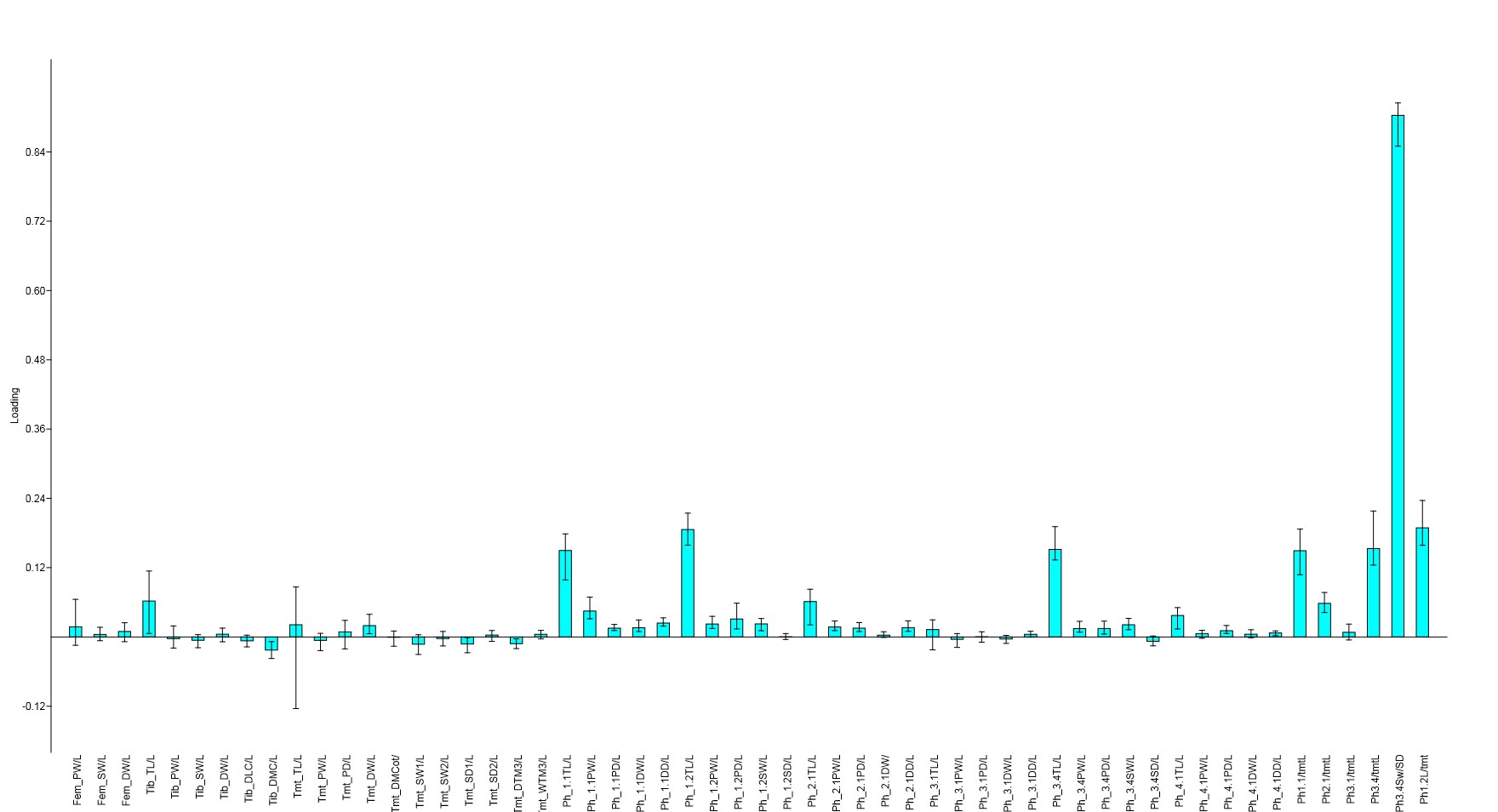


SI 4: Figure 4. A Loadings plot showing the impact of individual variables on the PCA.
